# SeSAM: software for automatic construction of order-robust linkage maps

**DOI:** 10.1186/s12859-022-05045-7

**Published:** 2022-11-19

**Authors:** Adrien Vidal, Franck Gauthier, Willy Rodrigez, Nadège Guiglielmoni, Damien Leroux, Nicolas Chevrolier, Sylvain Jasson, Elise Tourrette, Olivier C. Martin, Matthieu Falque

**Affiliations:** 1 Université Paris-Saclay, INRAE, CNRS, AgroParisTech, GQE - Le Moulon, 91190 Gif-sur-Yvette, France; 2 INRAE, Unité de Mathématiques et Informatique Appliquées - Toulouse, Toulouse, France; 3 Université Paris-Saclay, CNRS, INRAE, Université Evry, Institute of Plant Sciences Paris-Saclay (IPS2), 91190 Gif-sur-Yvette, France; 4 Université Paris Cité, CNRS, INRAE, Univ Evry, Institute of Plant Sciences Paris-Saclay (IPS2), 91190 Gif-sur-Yvette, France

**Keywords:** Genetic mapping, Linkage, Automated software, Seriation, Marker order robustness

## Abstract

**Background:**

Genotyping and sequencing technologies produce increasingly large numbers of genetic markers with potentially high rates of missing or erroneous data. Therefore, the construction of linkage maps is more and more complex. Moreover, the size of segregating populations remains constrained by cost issues and is less and less commensurate with the numbers of SNPs available. Thus, guaranteeing a statistically robust marker order requires that maps include only a carefully selected subset of SNPs.

**Results:**

In this context, the SeSAM software allows automatic genetic map construction using seriation and placement approaches, to produce (1) a high-robustness *framework* map which includes as many markers as possible while keeping the order robustness beyond a given statistical threshold, and (2) a high-density *total* map including the framework plus almost all polymorphic markers. During this process, care is taken to limit the impact of genotyping errors and of missing data on mapping quality. SeSAM can be used with a wide range of biparental populations including from outcrossing species for which phases are inferred on-the-fly by maximum-likelihood during map elongation. The package also includes functions to simulate data sets, convert data formats, detect putative genotyping errors, visualize data and map quality (including graphical genotypes), and merge several maps into a consensus. SeSAM is also suitable for interactive map construction, by providing lower-level functions for 2-point and multipoint EM analyses. The software is implemented in a R package including functions in C++.

**Conclusions:**

SeSAM is a fully automatic linkage mapping software designed to (1) produce a framework map as robust as desired by optimizing the selection of a subset of markers, and (2) produce a high-density map including almost all polymorphic markers. The software can be used with a wide range of biparental mapping populations including cases from outcrossing. SeSAM is freely available under a GNU GPL v3 license and works on Linux, Windows, and macOS platforms. It can be downloaded together with its user-manual and quick-start tutorial from ForgeMIA (SeSAM project) at https://forgemia.inra.fr/gqe-acep/sesam/-/releases

**Supplementary Information:**

The online version contains supplementary material available at 10.1186/s12859-022-05045-7.

## Background

Genetic linkage maps are representations of positions of polymorphic genetic elements along chromosomes, based on allele co-segregation patterns. Map distances are calculated from the frequency of meiotic crossovers between two linked loci; in the first historical maps, such frequencies were inferred from the segregation of phenotypes determined by two linked genes [1]. With the development of DNA technologies, the number of genetic markers increased, allowing genetic maps to become saturated, which means that any locus on the genome is significantly linked with at least one marker of the map [2, 3]. Linkage maps initially played an important role in unraveling the general organization of genomes [4], and in spite of genome sequencing becoming more and more accessible for structural genomics, they are still of great use e.g. for QTL detection via linkage or association studies, to help the orienting and placing of sequence contigs during genome assembly [5], or to detect errors a posteriori in assembled genomes [6].

In practice, genetic maps are built from observing the allelic segregation of polymorphic markers in mapping populations produced by different types of crossing schemes [7]. Biparental populations are the most frequently used, typically based on either two homozygous parents or two (partly) heterozygous parents as in the case of Cross-Pollinated (CP) populations of many forest or fruit trees. The latter case involves more complex algorithms because current genotyping technologies do not directly provide long haplotype information, so the phase between multi-locus allelic configurations is unknown and must be inferred [8, 9]. Populations obtained from homozygous parents can be backcross (BC) or Doubled-Haploids (DH) which are very similar to BC with regards to map estimation, F2–F*n*, Recombinant Inbred Lines (RIL) [10], or Intermated Recombinant Inbred lines (IRIL) populations. IRILs include some generations of random intermating between the F2 and the inbreeding generations, thereby increasing the number of crossovers captured and thus the resolution of the map for a given population size [11, 12].

The usual process for genetic map construction involves three successive steps [13] corresponding to (1) determination of linkage groups (when the map is saturated, linkage groups correspond to chromosomes), (2) ordering of markers in each linkage group, and (3) estimation of genetic distances between adjacent ordered markers. A lot of algorithmic effort has been made in particular for the ordering step, because as soon as the number of markers is not very small, it becomes unfeasible to evaluate an objective function for each possible order (*m*!/2 orders if *m* is the number of markers). This problem, which is very similar to the Traveling Salesman Problem [14], is usually addressed in mapping softwares via different heuristics to escape this combinatorial explosion (see some examples in [13, 15–18]; non-exhaustive list shown as Additional file 1: Table S1). The ordering algorithmic problem obviously becomes more difficult with recent genotyping technologies (including genotyping-by-sequencing) which can produce millions of SNPs. But with such technologies, an even more limiting issue is that whatever the algorithm, the information allowing ordering lies in the crossovers arising in the population, and thus scales up only with population size, which is generally much more expensive to increase than marker number. A consequence is that if one wants to fix a minimum threshold for a robustness statistical criterion (for instance the minimum logarithm of odds (LOD) between the best order found and any other order), the number of markers in the map will be limited for a given population type and size: the higher the threshold, the lower the number of markers which can be included in the map. For usual levels of threshold (e.g. LOD = 3) and large data sets, the maximum number of markers in the map will most often represent only a fraction of the SNPs available. The problem then translates into chosing the largest subset of markers which allows the order to be statistically robust at a given threshold. Here we propose the SeSAM (Seriation-based Suite for Automatic Mapping) package as a way to address the genetic mapping problem from this perspective.

Another consequence of the evolution of genotyping technologies is the number of missing data and/or genotyping errors, which can vary a lot depending on the approach used. For example, genotyping using low-coverage NGS sequencing [19–21] can produce many missing data which, depending on the protocol used for library preparation, can be distributed differently in the genome in different individuals of the mapping population. This is of particular concern for linkage analysis because detecting crossovers between two markers requires valid data in both markers. In multipoint estimations however, it is possible to impute part of the missing information for instance through Expectation–Maximization (EM) [22] algorithms, and it is possible to make use of data for genotype likelihoods [23], but beyond a certain level of missing data, map estimation always becomes challenging. The problem of genotyping errors is even more important when the number of markers becomes very large: each miscalled allele can produce a singleton interpreted as the result of two crossovers, thus for a given rate of genotyping errors, the more markers in the map, the more dramatically map length will be artificially inflated, and marker ordering altered [24]. A number of algorithms identify singletons and putative erroneous data; replacing them by missing data limits their effect on the mapping outcome [25–28]. Conversely, it is also possible to identify markers that have a very low probability of displaying genotyping errors based on redundancy ("twins" approach [29–31]).

**Fig. 1:**
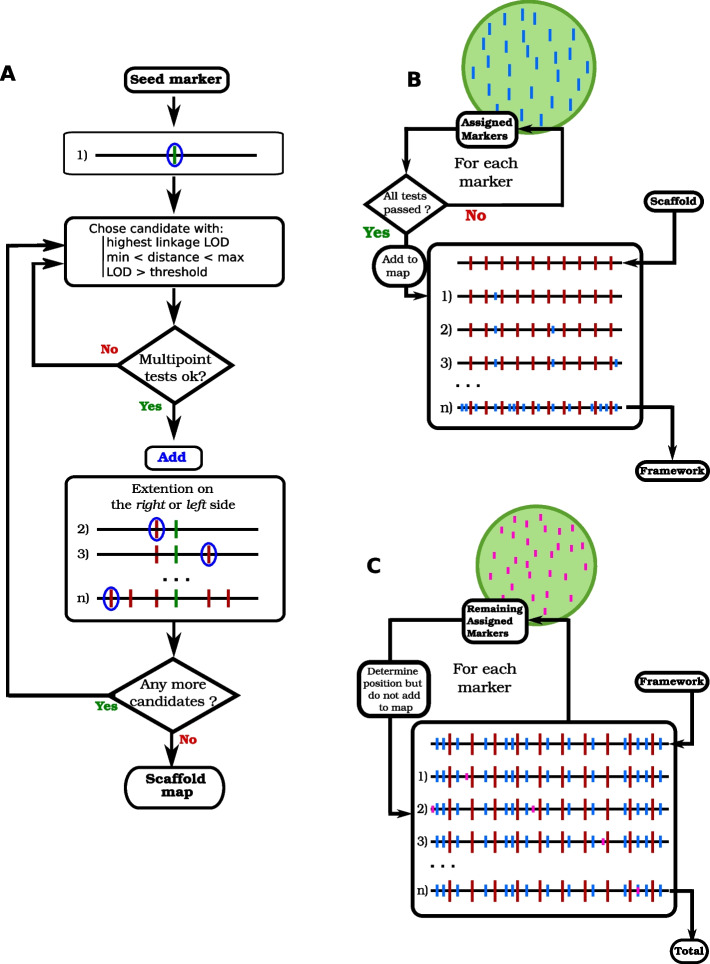
General algorithm of automatic map construction in SeSAM. **A**: Construction of the *scaffold* map by seriation. **B**: Densification of the scaffold to produce the high-robustness *framework* map. **C**: Placement of all polymorphic markers to produce the high-density *total* map

Numerous software tools have been developed for genetic mapping (see non-exhaustive list as Additional file 1: Table S1). Many of them feature sophisticated algorithms for marker ordering, some even include several different algorithms which can be compared to each other to assess the robustness of their outcome (see for instance [15]). Most of the time, the main goal is to achieve optimal performances for finding the best order between all markers of a given linkage group (sometimes the 2nd, 3rd, etc.… best maps are also provided). In some cases however, particularly (but not only) when population size is limited, an interesting alternative is the "bin-mapping" strategy [32–34]. In that approach, (1) a *framework* core skeletal map including only a subset of selected markers is produced to ensure an order robustness statistically supported at a desired threshold. The larger the mapping population, the more markers are included in this framework map. (2) Then all remaining polymorphic markers are *placed* within one of the bins delimited by the framework markers and their relative map position is calculated within the bin. Thus even though the order between close "placed" markers is not statistically supported, the order between each placed marker and the framework markers is. This strategy has several advantages: (1) the position of all markers can still be estimated precisely, while escaping the challenging computational problem of ordering too many markers, (2) the number of markers may become as high as desired, the computation time will remain close to linear with that number, (3) the uncertainty on the order of very close placed markers has no consequence on the estimated map length, and thus that uncertainty is no longer a problem for many applications. In practice, such a bin-mapping strategy is usually carried out through an interactive process between an expert user and computer programs. Tools have been developed to automate the placing step [33], but to our knowledge, there is today no integrated software able to carry out a complete automated mapping process based on the bin-mapping strategy. So here we propose the SeSAM package, which automatically chains all steps necessary for genetic map construction based on this approach (Fig. 1), the two main steps being: (1) producing a *framework* map by selecting an optimal subset of markers from the initial data set, so order robustness can be statistically supported, and (2) producing a *total* map by placing all remaining polymorphic markers one by one into that framework. In the first step, the determination of linkage groups is done during the elongation of an ordered low-density high-robustness map (*scaffold* map) through a seriation-based algorithm (Fig. 1A) [35, 36], after which iterative densification of that scaffold leads to adding as many markers as possible while keeping the order robust at the desired threshold, which produces the *framework* map (Fig. 1B). During this process, putative genotyping errors are detected and put aside to limit artifactual inflation of map length and ordering flaws. Finally, the remaining markers are *placed* on the framework (Fig. 1C).

## Implementation

SeSAM is implemented as a R package, which allows to easily chain map construction to any other input data formatting (or map output exploiting) R script. The package includes C++ functions for its most computation-intensive parts (e.g. likelihood computations, EM algorithms). A detailed description of all algorithms and functions of SeSAM is provided in the user manual available as Additional file 2, and also from ForgeMIA at https://forgemia.inra.fr/gqe-acep/sesam/-/releases. The main functionality of SeSAM lies in the function *autoMap()*, which is a completely automated pipeline going through different main steps carried out by the following functions: *loadData()*, which reads and checks segregation data, *generateSeeds()*, which draws the seed markers used to initiate the seriation process, *buildScaffold()*, which extends a highly robust sparse map by seriation from the seed markers, *assignment()*, which assigns all polymorphic markers to a linkage group, *buildFramework()*, which densifies the scaffolds with the maximum possible number of markers while keeping a given statistical level of order robustness to the framework map, and *placement()*, which adds all remaining markers to the framework map without ensuring a statistical value for order robustness. Missing data are imputed in all multipoint calculations via an EM algorithm, and putative genotyping errors are detected and taken into account via two different methods. Finally, the SeSAM package includes a toolbox of functions to perform various types of format conversions on data files, interactive step-by-step custom mapping processes, and assessment of map and data quality through different types of graphs. More detailed information about these different functions is available as Additional file 1: Text S1, and in the reference user manual available as Additional file 2 and containing a quick-start guide.

## Results and discussion

**Fig. 2:**
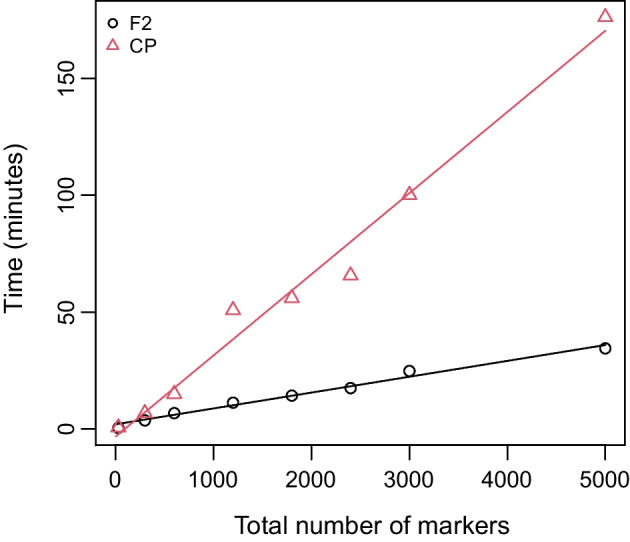
SeSAM computation time for automatic *total* map construction, as a function of the density of markers in a F2 (black circles and line) and in a CP (red triangles and line) population of 100 individuals. Data were simulated using the SeSAM function *simulatePop()* for two chromosomes (100 and 200 cM) with markers regularly spaced. Lines were obtained from linear regression y = a.x + b (a = 0.007, b = 1.9 for F2 and a = 0.035, b = 3.4 for CP)

**Fig. 3:**
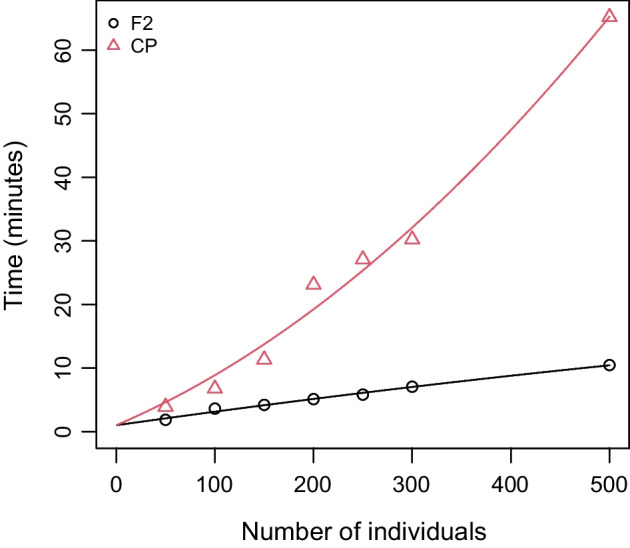
SeSAM computation time for automatic *total* map construction, as a function of the number of individuals in a F2 (black circles and line) and in a CP (red triangles and line) population. Data were simulated using the SeSAM function *simulatePop()* for two chromosomes (100 and 200 cM) with markers regularly spaced at a density of 1 marker/cM. For F2, the line was obtained from linear regression y = a.x + b (a = 0.022, b = 0.99). For CP, the line was obtained from non-linear regression y = a.x^2^ + b.x + c (a = 0, b = 0.066, c = 0.99)

**Fig. 4:**
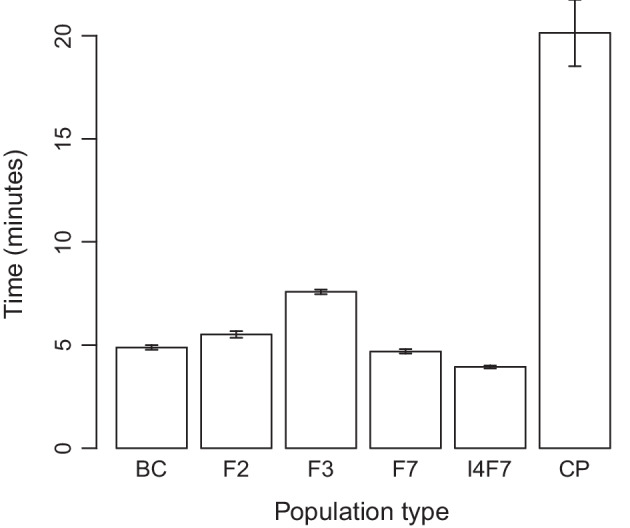
SeSAM computation times for automatic *total* map construction for different types of mapping populations of 200 individuals. Data were simulated using the SeSAM function *simulatePop()* for two chromosomes (100 and 200 cM) with markers regularly spaced at a density of 1 marker/cM. Error bars indicate 95% confidence intervals based on nine independent replicates with different seeds for the random number generator used to simulate the data

*Behavior with number of markers and population size* was assessed by simulating data sets for different population types, numbers of markers, and numbers of individuals. SeSAM was run on a desktop computer using 4 cores Intel(R) Core(TM) i7-4790 CPU @ 3.60 GHz (2 threads per core) under the Debian 11 Linux OS, using SeSAM default parameters. The scripts and data used to produce these benchmarking results are available in Additional file 3. Computation time for total maps construction was more or less linear with the number of markers for F2 and CP populations (Fig. 2). It was also close to linear with the number of individuals of the F2 population, but close to quadratic with the number of individuals for the CP population (Fig. 3). Finally, Fig. 4 shows that CP data sets necessitate substantially longer computation time than other population types, which is expected due to the extra phasing process required for such data.

To assess the quality of the maps produced by SeSAM, we generated arbitrary reference maps with different marker densities and used them to simulate segregation data. Then, for different numbers of individuals or markers (same maps as for Figs. 2 and 3), we measured the deviation from colinearity (through Spearman’s rank correlation) and the map length ratio between the *framework* (or *total)* map computed from these segregation data and the initial reference map. Finally, we measured the inclusion rate, that is the proportion of markers in the data set that could be included in the map (see Additional file 1: Tables S2 and S3). We see that in F2 or CP populations, the framework maps were always perfectly colinear to the reference map. The *total* maps were also perfectly colinear to their reference map, except for the F2 population with only 50 individuals. The map length ratios were always between 0.88 and 1.03 for the framework map except for very small F2 and CP populations (50 individuals) for which the scaffold could not meet the robustness criteria up to the extremities of the chromosomes and thus dropped some terminal regions. Similar behaviours were observed for the total maps. All markers of the data sets could be included into the total maps except in the case of the F2 population of 50 individuals for which the framework did not cover the whole of the chromosomes as seen before. On the other hand, when looking at the framework maps, we see as expected that their inclusion rate increases with population size, and decreases with the number of markers.

**Fig. 5:**
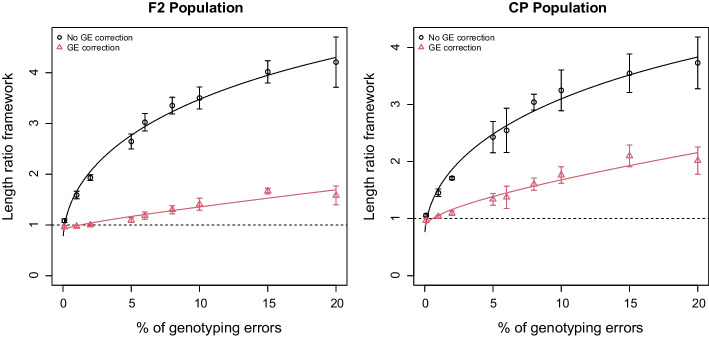
Map length ratio between the *framework* map and the simulated reference map after SeSAM automatic map construction, as a function of the percentage of genotyping errors in a F2 (left panel) or CP (right panel) population of 200 individuals, without and with activating the genotyping error correction option of SeSAM (black circles and red triangles respectively). Data were simulated using the SeSAM function *simulatePop()* for two chromosomes (100 and 200 cM) with markers regularly spaced at a density of 1 marker/cM, with increasing proportions of genotyping errors uniformly distributed along chromosomes. Lines were obtained by non-linear regression y = a*sqrt(x) + b*x + c (values of (a,b,c) without and with genotyping error correction, leading to respectively (1.09, − 0.06, 0.64) and (0.07, 0.025, 0.89) for F2, and respectively (0.93, − 0.049, 0.64) and (0.20, 0.022, 0.84) for CP). Error bars indicate 95% confidence intervals based on five independent replicates with different seeds for the random number generator used to simulate the data. Dotted lines indicate the theoretical outcome of a perfect genotyping error correction (y = 1)

*Sensitivity to data quality* was assessed by simulating data sets with increasing rates of genotyping errors up to extremely high rates (20%). We chose to distribute false data rates uniformly across markers and individuals, although the *simulatePop()* function of SeSAM is able to use Gamma distributions which allow to slide continuously from L-shaped to almost symmetrical distributions. The effect of increasing rates of erroneous data on map quality is shown in Fig. 5 for F2 and CP populations without and with activating the error correction option of SeSAM. In both populations, the artefactual inflation of the map due to the genotyping errors is strongly reduced by the error correction algorithm, although high rates of errors cannot be completely corrected, particularly in CP populations. However, in most real data sets, error rates are generally expected to be under 5%, so in such cases, SeSAM correction mostly avoids significant map inflation due to such errors.

*Comparison with other existing softwares* To assess how the level of map quality achieved by SeSAM compares with that of other mapping softwares currently available, we generated simulated data sets with different numbers of individuals and markers, and ran them with SeSAM as well as with four different programs: IciMapping, ASMap, MapDisto, and TSPmap (see Additional file 1: Table S4). We also tried to use HighMap, but we could not obtain the software from the address mentioned in the paper. All tools tested excepted TSPmap produced high-quality *total* maps, showing high colinearity and similar lengths when compared to the theoretical map used to simulate the segregation data. However, with increasing numbers of markers (> = 10,000), we couldn't get some of the softwares to complete the mapping (see Additional file 1: Table S4). Computation times varied a lot between programs, with Lep-map performing much faster than all others, and SeSAM being in the average of the remaining ones. Overall, SeSAM produces maps with at least similar quality as the other softwares tested. Using SeSAM thus allows to have a fully automatic tool to produce *total* maps with a level of quality similar to most other software currently available, but contrary to those other programs, in addition to producing a total map with all polymorphic markers, SeSAM also automatically selects an optimally large subset of markers to produce a *framework* map statistically robust at any desired LOD threshold.

*Examples with biological data* To illustrate how SeSAM can perform with real biological data, mapping results obtained from five anonymized experimental data sets from agricultural plant species are presented in Additional file 1: Table S5 and Fig. S1–S5. The corresponding anonymized data sets are available as Additional file 4. The number of markers included in the *framework* map was always lower than the total number of polymorphic markers, because no more markers could be included without losing the order robustness at the given default LOD threshold (3.0). In BC_ano, RIL1_ano, and CP_ano data sets, which have small population sizes, the frameworks include less markers than in F2_ano and RIL2_ano, which have larger populations (see Additional file 1: Table S5). This is expected because there are more informative crossovers contributing to the order information in the latter. Moreover, the backcross-derived BC_ano data contain less crossovers (only one effective meiosis) than the other populations, which contributes to the fact that relatively few markers could be incorporated to the BC_ano framework map. Finally, with similar population sizes, the CP_ano map included less markers in its framework map than RIL1_ano. This is partly due to the fact that not all 2-point marker configurations are informative in CP populations (e.g. there is no linkage information between one male pseudo-backcross marker and one female pseudo-backcross marker). As expected, the number of markers in the framework map is thus commensurate with population size and population type since this ensures statistically supported marker orders. Considering now the *total* maps obtained after placement, they include almost all polymorphic markers for all data sets, the few non-mapped markers being unlinked to any linkage group, or linked to several linkage groups with similar LODs.

To visualy assess the quality of maps produced, SeSAM generates heat maps of pairwise 2-point LOD matrices. If the quality of the map is good, such heat maps should display a smooth decreasing gradient when going away from the diagonal (see left panels of Additional file 1: Figs. S1–S5). Another useful graph generated by SeSAM to assess map quality is the Marey map, which represents the genetic positions *vs* the physical positions of the markers. The derivative of the Marey map curve gives the local values of recombination rate along the chromosomes (called recombination landscape). If the quality of both physical and genetic maps is high, Marey maps are supposed to be smooth and always increasing (see right panels of Additional file 1: Figs. S2–S5). The large flat regions observed with BC_ano, F2_ano, RIL2_ano, and CP_ano typically correspond to the low peri-centromeric recombination rates. For the BC_ano data set however (see Additional file 1: Fig. S1), the Marey map is non-monotonic. Since the 2-point linkage matrix indicates a high-quality genetic mapping, the quality of the physical map may be questionable here. Elsewhere, the case of the RIL1_ano data set illustrates the possibilty of using a previously existing genetic map to guide the choice of the seed markers to initiate the seriation process, when no physical map is available. In such cases, the ‘phyMap.txt’ file actually contains a genetic map, so the ‘Marey map’ obtained has an almost constant slope, but it may also be used to compare recombination landscapes between different crosses.

Finally, using the same general algorithm as SeSAM, but with earlier generations of codes, we already produced genetic maps used in several published studies on Maize [6, 34, 37–42], Pea [43–45], and Faba bean [46].

## Conclusions

Compared to existing mapping software, SeSAM is to our knowledge the only one to carry out a completely automatic bin-mapping procedure producing first a mid-density *framework* map from an optimized subset of markers which allow the order to be statistically supported at the desired statistical threshold, and then a high-density *total* map including nearly all polymorphic markers, but preserving the global structure and length of the framework map. SeSAM is freely available to all users, including the source code, and is compatible with Linux, macOS, or Windows platforms.

## Availabillity and requirements

Project name: SeSAM.

Project home page: https://forgemia.inra.fr/gqe-acep/sesam

Operating systems: GNU Linux, macOS (> = 10.13), Windows10.

Programming language: R, C++

Other requirements: the following C++ libraries are required when compiling the package from source: gmp, boost-dev, boost-math (> = 1.56).

License: GNU GPL v3.

Any restrictions to use by non-academics: none.

## Supplementary Information

**Additional file 1**: **Text S1**. Description of the main functions used in SeSAM. **Table S1**. Non-exhaustive list of software tools available for linkage mapping. **Table S2**. Quality assessment of the maps produced using SeSAM in the benchmarks presented in Fig. 2. **Table S3**. Quality assessment of the maps produced using SeSAM in the benchmarks presented in Fig. 3. **Table S4**. Comparison of the quality of maps produced by SeSAM and four of the other mapping softwares listed in Additional file 1: Table S1. **Table S5**. Summary of maps obtained with SeSAM using experimental data sets from three agricultural plant species. **Fig. S1**. Map quality assessment graphs for SeSAM when using the BC_ano experimental data set (BC1 population). **Fig. S2**. Map quality assessment graphs for SeSAM when using the F2_ano experimental data set (F2 population). **Fig. S3**. Map quality assessment graphs for SeSAM when using the RIL1_ano experimental data set (RIL population). **Fig. S4**. Map quality assessment graphs for SeSAM when using the RIL2_ano experimental data set (RIL population). **Fig. S5**. Map quality assessment graphs for SeSAM when using the CP_ano experimental data set (CP population from heterozygous outbred parents).

**Additional file 2**: Reference User Manual of SeSAM, including a quick-start tutorial.

**Additional file 3**: Data and scripts used to carry out the benchmarking of SeSAM based on simulated data sets and to produce Figures 2, 3, 4, 5, and Supplementary Tables S2 and S3.

**Additional file 4**: Anonymized experimental mapping data sets from agricultural plants, used to produce the maps presented in Supplementary Table S5 and Supplementary Figures S1 to S5: BC_ano_segData.raw. BC_ano_phyMap.txt. F2_ano_segData.raw. F2_ano_phyMap.txt. RIL1_ano_segData.raw. RIL1_ano_phyMap.txt. RIL2_ano_segData.raw. RIL2_ano_phyMap.txt. CP_ano_segData.gen. CP_ano_phyMap.txt.

## Data Availability

All data and codes generated or analysed during this study are included in this published article and its supplementary information files.

## Abbreviations

CP: Cross-pollinated
BC: Back-cross
DH: Doubled-haploid
EM: Expectation–maximization
IRIL: Intermated recombinant inbred line
LOD: Logarithm of odds
RIL: Recombinant inbred line
SNP: Single-nucleotide polymorphism
