# Supplementary material for: SeSAM: software for automatic construction of order-robust linkage maps
Source: BMC Bioinformatics. 2022 Nov 19;23:499. doi: 10.1186/s12859-022-05045-7 (PMC9675223; doi:10.1186/s12859-022-05045-7)
Supplement: Supplementary file 1 — Additional file 1: Text S1. Description of the main functions used in SeSAM. Table S1. Non-exhaustive list of software tools available for linkage mapping. Table S2. Quality assessment of the maps produced using SeSAM in the benchmarks presented in Fig. 2. Table S3. Quality assessment of the maps produced using SeSAM in the benchmarks presented in Fig. 3. Table S4. Comparison of the quality of maps produced by SeSAM and four of the other mapping softwares listed in Additional file 1: Table S1. Table S5. Summary of maps obtained with SeSAM using experimental data sets from three agricultural plant species. Fig. S1. Map quality assessment graphs for SeSAM when using the BC_ano experimental data set (BC1 population). Fig. S2. Map quality assessment graphs for SeSAM when using the F2_ano experimental data set (F2 population). Fig. S3. Map quality assessment graphs for SeSAM when using the RIL1_ano experimental data set (RIL population). Fig. S4. Map quality assessment graphs for SeSAM when using the RIL2_ano experimental data set (RIL population). Fig. S5. Map quality assessment graphs for SeSAM when using the CP_ano experimental data set (CP population from heterozygous outbred parents). [file 12859_2022_5045_MOESM1_ESM.pdf]

Supplementary Data

Supplementary Text S1. Description of the main functions used in SeSAM.

The *loadData()* function reads input data files and checks for data format consistency, then selects a *strict* set of markers based on their low missing data rate and low-bias segregation. This strict set is then analyzed for redundancy (two markers are considered redundant if the data indicate no crossover between them) and a *reduced* core set of strict markers without redundancy is produced. This reduction step is particularly relevant to map very large numbers of markers (e.g. from genotyping-by-sequencing), because the number of markers in the reduced core set is limited by the number of crossovers captured, and thus by the size of the mapping population, which for practical reasons can never be very large, contrary to the number of markers. Moreover, markers taken as representatives of a group of at least two co-segregating markers ("twins") will be preferentially used in further mapping processes because genotyping errors in such twins are very unlikely. In the case of CP populations from heterozygous parents, all possible alternative phases of a given marker are initially deployed as different markers, which will be put in competition with each other in further two-point linkage analysis steps to determine the correct phase by Maximum-Likelihood (see User Manual in Additional file 2). Once this phasing process is completed for one marker, the wrong phases of that marker will be then removed from the data set for the rest of the analyses.

The *generateSeeds ()* function then selects *seed* markers to be used to initiate the seriation processes; seeds are randomly drawn for each chromosome based on a previous (e.g. physical) map provided as input. In case no such previous map is available, the function *putativeLGs()* builds a set of linkage groups (LGs) by clustering markers based on a seriation approach, which uses some of the functions used for mapping, where scaffold maps (see *buildScaffold* below) are constructed iteratively until no markers remain unassigned (see *assignment* below). From each of these LGs, one or more seed markers are then picked randomly after checking that there is no cross-linkage between different chromosomes.

The *buildScaffold()* function is then called several times to elongate by seriation one low-density highly robust *scaffold* map from each seed marker. The seriation process ensures a very high level of marker order robustness in that scaffold by successively extending a map extremity with the marker having the *strongest linkage* to that extremity, while keeping a *minimum distance* between these two markers to force the presence of enough crossovers to faithfully discriminate order hypotheses (Figure 1A). Different seeds from the same chromosome can be used as replicates to control the dependency of the seriation process on the initial marker. All scaffold replicates are then cleaned from possible cross-linking between different chromosomes, and the replicate of each chromosome with the highest number of markers is selected for subsequent steps, so the procedure ends with one scaffold map per chromosome.

The *assignment()* function then uses the scaffold maps to test their linkage with all remaining polymorphic markers of the data set, and assigns each marker to a chromosome by comparing its two highest linkages to the chromosome scaffolds.

The *buildFramework()* function then sequentially tries to add each remaining polymorphic marker into the map of the chromosome it was assigned to, starting from the scaffold map and taking into account every previously added marker in all subsequent steps. A marker is only incorporated if the local order robustness is still statistically supported at the desired threshold once the marker is inserted (Figure 1B). This step produces the *framework* map, which will be used as the core skeletal map for subsequent bin mapping. This map is highly robust and includes a number of markers optimally commensurate with the size of the mapping population. For whatever application where the order of markers is critical (e.g. for QTL interval mapping or whenever map length matters), this framework map can be used as the ultimate output of the mapping process, although the automated part of SeSAM implemented in the function *autoMap()* proceeds further to the placement step.

The *placement()* function then determines the position of the remaining markers one by one on the framework map, but does not incorporate them into the map (Figure 1C). In the previous steps, only the

strict (high-quality) markers from the reduced set were used to build the scaffold and framework maps, whereas now all remaining polymorphic markers are placed. Indeed, possible low-quality markers (e.g. with high level of missing or erroneous data, or segregation distortion) here do not alter the mapping of any other marker, contrary to standard mapping procedures. The resulting *total* map has practically almost no limit in marker number since its computation time is linear with the number of placed markers, and the software proceeds by loading successive batches of markers, so memory size is not an issue.

Putative genotyping errors are identified *via* the occurrence of singletons, that is when in a triplet of ordered markers belonging to the framework, the genotype of the middle marker is different from the shared genotype of its two neighbors in a given individual of the population. Singletons can be produced by genotyping errors or by having two close crossovers in the same individual. Genetic interference strongly lowers the probability that two close crossovers occur in the same meiosis, but most types of mapping populations involve several independent meioses. The only exception is the case of BC populations, these involving a single effective meiosis; nevertheless, very close crossovers can occur there even if only relatively rarely. However, even if two close crossovers occur on the bivalent in the same meiosis, they do not always affect the same chromatids, and in practice, singletons caused by true double crossovers are rare, while genotyping errors almost always produce singletons. Thus, in SeSAM, putative genotyping errors are detected based on singletons in the framework map, and replaced by the corresponding EM-imputed allele. This may introduce a small bias tending to reduce map length, but strongly reduces the consequences of genotyping errors on the map (see Results).

Interactive mode: As an alternative to the automated pipeline *autoMap()*, SeSAM also provides lower-level functions that can be used for a more interactive process. Examples include computing two-point LOD or distance matrices, *try()* that provides the best interval for one marker to be placed on a previous map, or computing multipoint distances and likelihoods of a given map using EM. Finally,

SeSAM also features a toolkit of functions for wider use, for instance to simulate maps and segregation data sets of different population types with various levels and distributions of missing data and genotyping errors, to merge *a posteriori* different maps into a single consensus map, or to assess the quality of data and maps through a variety of graphical displays including: graphical genotypes (which can indicate outlier individuals putatively obtained through pollen contamination), Marey maps, and linkage matrix heat maps. All such functions are listed and explained in the User Manual (Additional file 2), and their use is also explained in the embedded R documentation of the functions.

Supplementary Table S1. Non-exhaustive list of software tools available for linkage mapping

Software	Supported populations	Reference
MapMaker	F2, BC, RIL, DH	(Lander *et al.*, 1987)
MapManager QTX	F2, BC, RIL, AIC, ABC	(Manly, Cudmore and Meer, 2001)
CarthaGene	BC, F2, RIL, phase-known CP	(de Givry *et al.*, 2004)
RECORD	F2, F3, BC, RIL	(Os *et al.*, 2005)
MadMapper	RIL	(Kozik, 2006)
AntMap	F2, BC, RIL, DH	(Iwata and Ninomiya, 2006)
OneMap	F2, BC, RIL, DH, CP	(Margarido, Souza and Garcia, 2007)
MSTMap	BC, RIL, DH,	(Wu *et al.*, 2008)
THREaD Mapper	F2, BC, RIL, DH	(Cheema, Ellis and Dicks, 2010)
JoinMap	F2, BC, RIL, DH, BCxFy, IxFy, CP	(Ooijen, 2011)
HighMap	F2, BC, DH, RIL, CP	(Liu *et al.*, 2014)
FsLinkageMap	F2, BC, DH, CP	(Tong, 2014)
IciMapping	F2, BC, DH, RIL, Multiparental	(Meng *et al.*, 2015)
MapDisto	F2, BC, RIL, DH	(Heffelfinger, Fragoso and Lorieux, 2017)
Lep-Map	F2, CP	(Rastas, 2013, 2017)
ASMap	F2, BC, RIL, DH	(Taylor and Butler, 2017)
TSPMap	Requires recomb matrix	(Monroe *et al.*, 2017, 2017)
BatchMap	CP parallelization of OneMap	(Schiffthaler *et al.*, 2017)

Cheema, J., Ellis, T. H. N. and Dicks, J. (2010) 'THREaD Mapper Studio: a novel, visual web server for the estimation of genetic linkage maps', *Nucleic Acids Research*, 38(suppl_2), pp. W188–W193. doi: 10.1093/nar/gkq430.

de Givry, S. *et al.* (2004) 'CarthaGene: multipopulation integrated genetic and radiation hybrid mapping', *Bioinformatics*, 21(8), pp. 1703–1704. doi: 10.1093/bioinformatics/bti222.

Heffelfinger, C., Fragoso, C. A. and Lorieux, M. (2017) 'Constructing linkage maps in the genomics era with MapDisto 2.0', *Bioinformatics*, 33(14), pp. 2224–2225. doi: 10.1093/bioinformatics/btx177.

Iwata, H. and Ninomiya, S. (2006) 'AntMap: Constructing Genetic Linkage Maps Using an Ant Colony Optimization Algorithm', *Breeding Science*, 56(4), pp. 371–377. doi: 10.1270/jsbbs.56.371.

Kozik, A. (2006) *Suite of Python MadMapper scripts for quality control of genetic markers, group analysis and inference of linear order of markers on linkage groups.* Available at: https://cgpdb.ucdavis.edu/XLinkage/MadMapper/.

Lander, E. S. *et al.* (1987) 'MAPMAKER: An interactive computer package for constructing primary genetic linkage maps of experimental and natural populations', *Genomics*, 1(2), pp. 174–181. doi: 10.1016/0888-7543(87)90010-3.

Liu, D. *et al.* (2014) 'Construction and Analysis of High-Density Linkage Map Using High-Throughput Sequencing Data', *PLOS ONE*, 9(6), p. e98855. doi: 10.1371/journal.pone.0098855.

Manly, K. F., Cudmore, Jr., Robert H. and Meer, J. M. (2001) 'Map Manager QTX, cross-platform software for genetic mapping', *Mammalian Genome*, 12(12), pp. 930–932. doi: 10.1007/s00335-001-1016-3.

Margarido, G. R. A., Souza, A. P. and Garcia, A. a. F. (2007) ‘OneMap: software for genetic mapping in outcrossing species’, *Hereditas*, 144(3), pp. 78–79. doi: 10.1111/j.2007.0018-0661.02000.x.

Meng, L. *et al.* (2015) ‘QTL IciMapping: Integrated software for genetic linkage map construction and quantitative trait locus mapping in biparental populations’, *The Crop Journal*, 3(3), pp. 269–283. doi: 10.1016/j.cj.2015.01.001.

Monroe, J. G. *et al.* (2017) ‘TSPmap, a tool making use of traveling salesperson problem solvers in the efficient and accurate construction of high-density genetic linkage maps’, *BioData Mining*, 10(1), p. 38. doi: 10.1186/s13040-017-0158-0.

Ooijen, J. W. V. (2011) ‘Multipoint maximum likelihood mapping in a full-sib family of an outbreeding species’, *Genetics Research*, 93(5), pp. 343–349. doi: 10.1017/S0016672311000279.

Os, H. V. *et al.* (2005) ‘RECORD: a novel method for ordering loci on a genetic linkage map’, *Theoretical and Applied Genetics*, 112(1), pp. 30–40. doi: 10.1007/s00122-005-0097-x.

Rastas, P. *et al.* (2013) ‘Lep-MAP: fast and accurate linkage map construction for large SNP datasets’, *Bioinformatics*, 29(24), pp. 3128–3134. doi: 10.1093/bioinformatics/btt563.

Rastas, P. (2017) ‘Lep-MAP3: robust linkage mapping even for low-coverage whole genome sequencing data’, *Bioinformatics*, 33(23), pp. 3726–3732. doi: 10.1093/bioinformatics/btx494.

Schiffthaler, B. *et al.* (2017) ‘BatchMap: A parallel implementation of the OneMap R package for fast computation of F1 linkage maps in outcrossing species’, *PLOS ONE*, 12(12), p. e0189256. doi: 10.1371/journal.pone.0189256.

Taylor, J. and Butler, D. (2017) ‘R Package ASMap: Efficient Genetic Linkage Map Construction and Diagnosis’, *Journal of Statistical Software*, 79(6). doi: 10.18637/jss.v079.i06.

Tong, C. (2014) *Manual of FsLinkageMap 2.1*. Available at: http://www.bioseqdata.com/FsLinkageMap/FsLinkageMap.pdf.

Wu, Y. *et al.* (2008) ‘Efficient and Accurate Construction of Genetic Linkage Maps from the Minimum Spanning Tree of a Graph’, *PLoS Genetics*. Edited by L. Kruglyak, 4(10), p. e1000212. doi: 10.1371/journal.pgen.1000212.

Supplemetary Table S2. Quality assessment of the maps produced using SeSAM in the benchmarks presented in Figure 2. The maps used to simulate the segregation data were used as reference maps. No linkage group error was observed. NbMrk chr1 (resp. NbMrk chr2): number of markers on chromosome 1 (resp. on chromosome 2). NonCol Frame (resp. NonCol Total): divergence from colinearity, calculated as one minus the Spearman rank correlation coefficient between the *framework* map (resp. the *total* map) and the reference map. LenRatio Frame (resp. LenRatio Total): map length ratio between the *framework* map (resp. the *total* map) and the reference map. InclRate Frame (resp. InclRate Total): inclusion rate: proportion of markers of the reference map present in the *framework* map (resp. in the *total* map).

Type	NbInd	NbMrk chr1	NbMrk chr2	NonCol Frame	NonCol Total	LenRatio Frame	LenRatio Total	InclRate Frame	InclRate Total
F2	100	10	20	0.00	0.00	0.91	0.93	0.97	1.00
F2	100	100	200	0.00	0.00	0.92	0.93	0.48	1.00
F2	100	200	400	0.00	0.00	0.94	0.94	0.32	1.00
F2	100	400	800	0.00	0.00	0.97	0.97	0.18	1.00
F2	100	600	1200	0.00	0.00	0.92	0.94	0.11	1.00
F2	100	800	1600	0.00	0.00	0.95	0.96	0.09	1.00
F2	100	1000	2000	0.00	0.00	0.91	0.92	0.07	1.00
F2	100	1667	3333	0.00	0.00	0.98	0.99	0.04	1.00
CP	100	10	20	0.00	0.00	0.91	0.94	0.93	1.00
CP	100	100	200	0.00	0.00	0.91	0.96	0.40	1.00
CP	100	200	400	0.00	0.00	0.95	0.96	0.25	1.00
CP	100	400	800	0.00	0.00	0.90	0.92	0.15	1.00
CP	100	600	1200	0.00	0.00	1.03	1.05	0.12	1.00
CP	100	800	1600	0.00	0.00	0.88	0.90	0.08	1.00
CP	100	1000	2000	0.00	0.00	0.94	0.95	0.07	1.00
CP	100	1667	3333	0.00	0.00	0.99	1.01	0.04	1,00

SupplementaryTable S3. Quality assessment of the maps produced using SeSAM in the benchmarks presented in Figure 3. Details of columns are the same as in Supplementary Table S2.

Type	NbInd	NbMrk chr1	NbMrk chr2	NonCol Frame	NonCol Total	LenRatio Frame	LenRatio Total	InclRate Frame	InclRate Total
F2	50	100	200	0.00	0.33	0.23	0.55	0.04	0.57
F2	100	100	200	0.00	0.00	0.92	0.93	0.48	1.00
F2	150	100	200	0.00	0.00	0.96	0.96	0.72	1.00
F2	200	100	200	0.00	0.00	0.96	0.97	0.87	1.00
F2	250	100	200	0.00	0.00	0.99	0.99	0.96	1.00
F2	300	100	200	0.00	0.00	0.98	0.98	0.96	1.00
F2	500	100	200	0.00	0.00	0.98	0.98	1.00	1.00
CP	50	100	200	0.00	0.00	0.73	0.85	0.16	1.00
CP	100	100	200	0.00	0.00	0.91	0.96	0.40	1.00
CP	150	100	200	0.00	0.00	0.90	0.92	0.46	1.00
CP	200	100	200	0.00	0.00	0.94	0.95	0.59	1.00
CP	250	100	200	0.00	0.00	0.96	1.20	0.62	1.00
CP	300	100	200	0.00	0.00	0.93	0.95	0.68	1.00
CP	500	100	200	0.00	0.00	0.98	0.98	0.81	1.00

SupplementaryTable S4. Comparison of the quality of maps produced by SeSAM and four of the other mapping softwares listed in Supplementary Table S1, on four different simulated data sets produced with the *simulatePop()* function of SeSAM: Sim1 (80 F7 individuals, 2 chromosomes: 100 and 200 cM, 500 and 100 markers), Sim2 (80 F7 individuals, 2 chromosomes: 100 and 200 cM, 200 and 1000 markers), Sim3 (80 F7 individuals, 1 chromosome: 100 cM, 10,000 markers), and Sim4 (70 F7 individuals, 1 chromosome: 100 cM, 50,000 markers). NonCol Total: divergence from colinearity, calculated as one minus the Spearman rank correlation coefficient between the total map obtained and the reference map. LenRatio Total: map length ratio between the total map obtained and the reference map. All programs were run with Kosambi mapping function and otherwise default parameters, without any optimization. SeSAM, ASMap, and TSPmap were run on a desktop computer with 8 cores Intel(R) Xeon (R) CPU E5-2623 v3 @ 3.00GHz under the Debian 11 Linux OS. MapDisto and IciMapping were run on a desktop computer with 4 cores Intel(R) Core(TM) i7-4790 CPU @ 3.60GHz under Windows 10 Professional. Runs longer than 24h without any change in the display were considered frozen and stopped.

**Software**	**Data**	**Run completed**	**Correct nb of LGs**	**NonCol Total**	**LenRatio Total**
ASMap	Sim1	yes	yes	$1.80 \cdot 10^{-4}$	0.854
IciMapping	Sim1	yes	yes	$1.70 \cdot 10^{-4}$	0.853
MapDisto	Sim1	yes	yes	$2.16 \cdot 10^{-4}$	0.841
SeSAM	Sim1	yes	yes	$0.00 \cdot 10^{-4}$	0.894
TSPmap	Sim1	yes	no (5 instead of 2)	NA	NA
ASMap	Sim2	yes	yes	$1.07 \cdot 10^{-4}$	0.888
IciMapping	Sim2	yes	yes	$1.09 \cdot 10^{-4}$	0.875
MapDisto	Sim2	yes	yes	$1.11 \cdot 10^{-4}$	0.866
SeSAM	Sim2	yes	yes	$1.36 \cdot 10^{-4}$	0.938
TSPmap	Sim2	yes	no (5 instead of 2)	NA	NA
ASMap	Sim3	yes	yes	$1.55 \cdot 10^{-4}$	0.877
IciMapping	Sim3	no (process frozen)	NA	NA	NA
MapDisto	Sim3	no (process frozen)	NA	NA	NA
SeSAM	Sim3	yes	yes	$1.37 \cdot 10^{-4}$	0.871
TSPmap	Sim3	no (process frozen)	NA	NA	NA
ASMap	Sim4	yes	yes	$1.88 \cdot 10^{-4}$	0.804
IciMapping	Sim4	no (runtime error)	NA	NA	NA
MapDisto	Sim4	no (out of memory)	NA	NA	NA
SeSAM	Sim4	yes	yes	$2.56 \cdot 10^{-4}$	0.792
TSPmap	Sim4	no (segfault)	NA	NA	NA

SupplementaryTable S5. Summary of maps obtained with SeSAM using experimental data sets from three agricultural plant species. NbInd: number of individuals in the population, NbPolym: number of polymorphic markers, NbFramework: number of markers robustly ordered in the framework map, NbTotal: number of markers included in the total map. The data sets used to compute these maps are available in Additional file 4. The data were obtained from plant breeding programs, they were anonymized by recoding marker names, normalizing physical positions to 0-10^6, and selecting a subset of randomly chosen chromosomes.

Data set	Type	NbInd	NbPolym	NbReduced	NbFramework	NbTotal
BC_ano	BC1	169	1371	529	222	1324
F2_ano	F2	355	5376	2057	1377	5352
RIL1_ano	RIL	184	641	641	448	641
RIL2_ano	RIL	480	2517	1475	856	2507
CP_ano	CP	182	2001	1171	268	1974

Supplementary Figure S1. Map quality assessment graphs for SeSAM when using the BC_ano experimental data set (BC1 population).

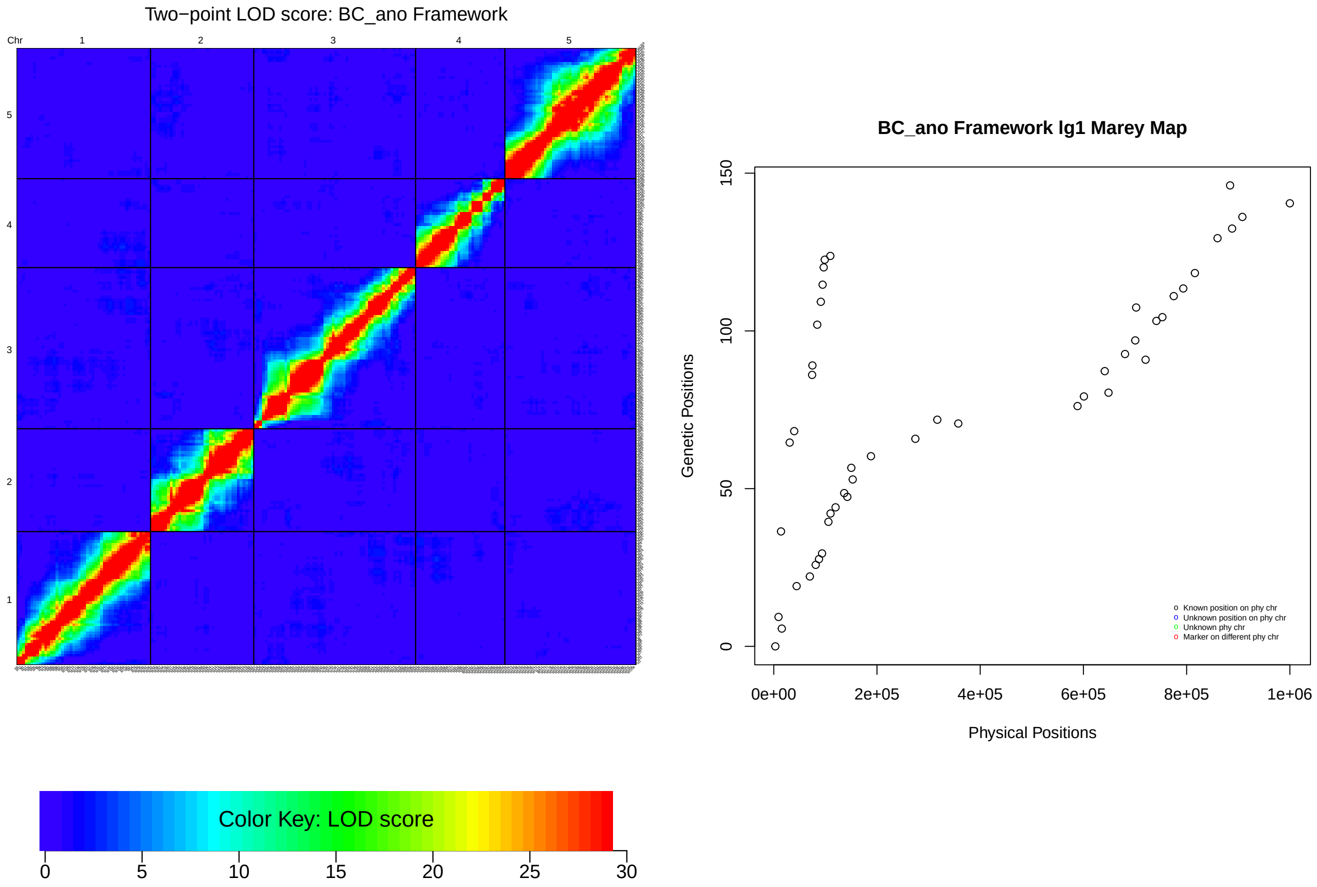

Left panel: pairwise 2-pt linkage LOD between markers of the framework map (all chromosomes). Right panel: Marey map of the framework map for chromosome 1. X-axis: relative physical positions (normalized to 0-10^6) of the markers given in the phyMap file. Y-axis: genetic position of the markers.

Supplementary Figure S2. Map quality assessment graphs for SeSAM when using the F2_ano experimental data set (F2 population).

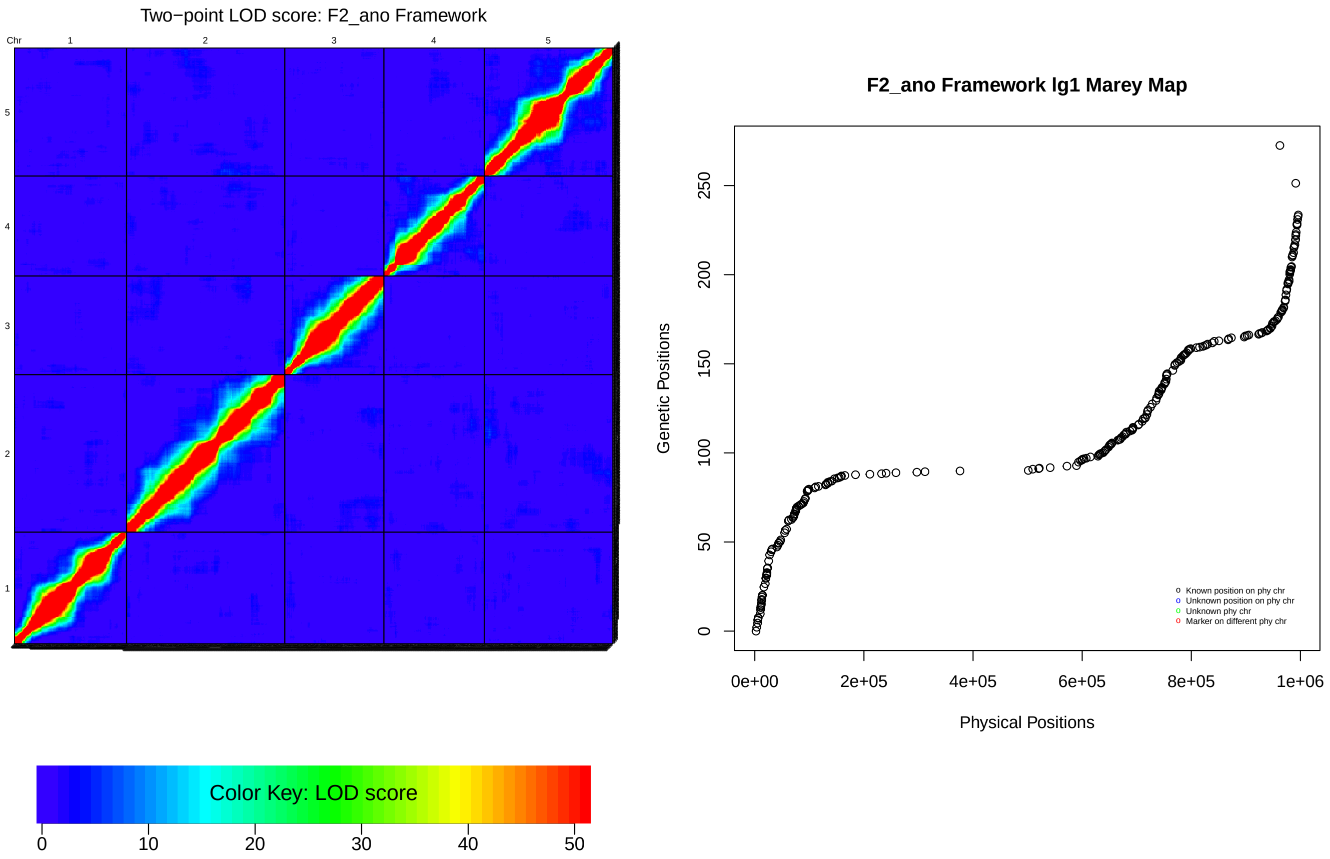

Left panel: pairwise 2-pt linkage LOD between markers of the framework map (all chromosomes). Right panel: Marey map of the framework map for chromosome 1. X-axis: relative physical positions (normalized to 0-10^6) of the markers given in the phyMap file. Y-axis: genetic position of the markers.

Supplementary Figure S3. Map quality assessment graphs for SeSAM when using the RIL1_ano experimental data set (RIL population).

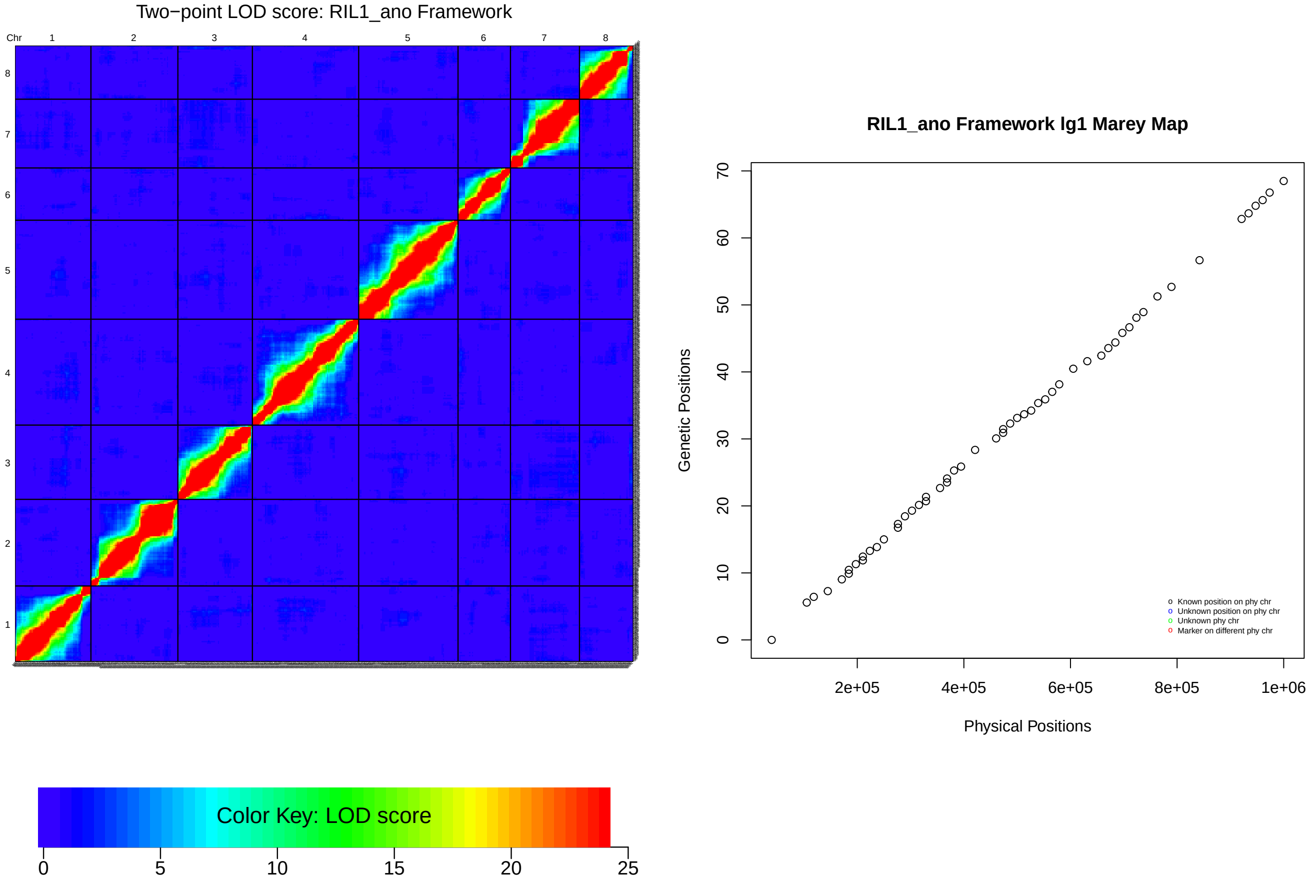

Left panel: pairwise 2-pt linkage LOD between markers of the framework map (all chromosomes). Right panel: Marey map of the framework map for chromosome 1. X-axis: relative positions (normalized to 0-10^6) of the markers given in the phyMap file. These positions are usualy physical positions, but here, in the absence of a physical map, an already available genetic map containing some of the markers was used as phyMap file to draw seed markers from different chromosomes. Y-axis: genetic position of the markers.

Supplementary Figure S4. Map quality assessment graphs for SeSAM when using the RIL2_ano experimental data set (RIL population).

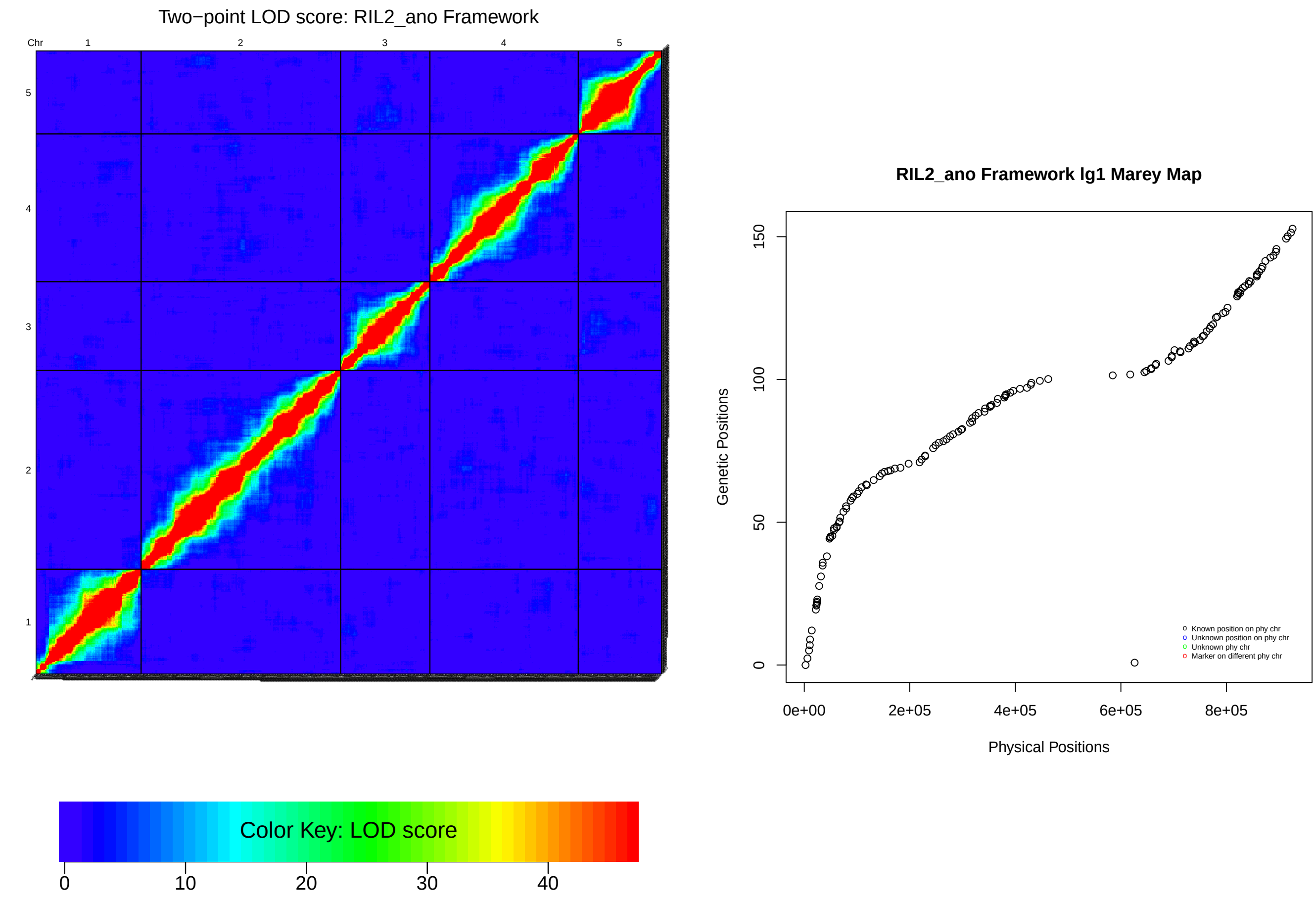

Left panel: pairwise 2-pt linkage LOD between markers of the framework map (all chromosomes). Right panel: Marey map of the framework map for chromosome 1. X-axis: relative physical positions (normalized to 0-10^6) of the markers given in the phyMap file. Y-axis: genetic position of the markers.

Supplementary Figure S5. Map quality assessment graphs for SeSAM when using the CP_ano experimental data set (CP population from heterozygous outbred parents).

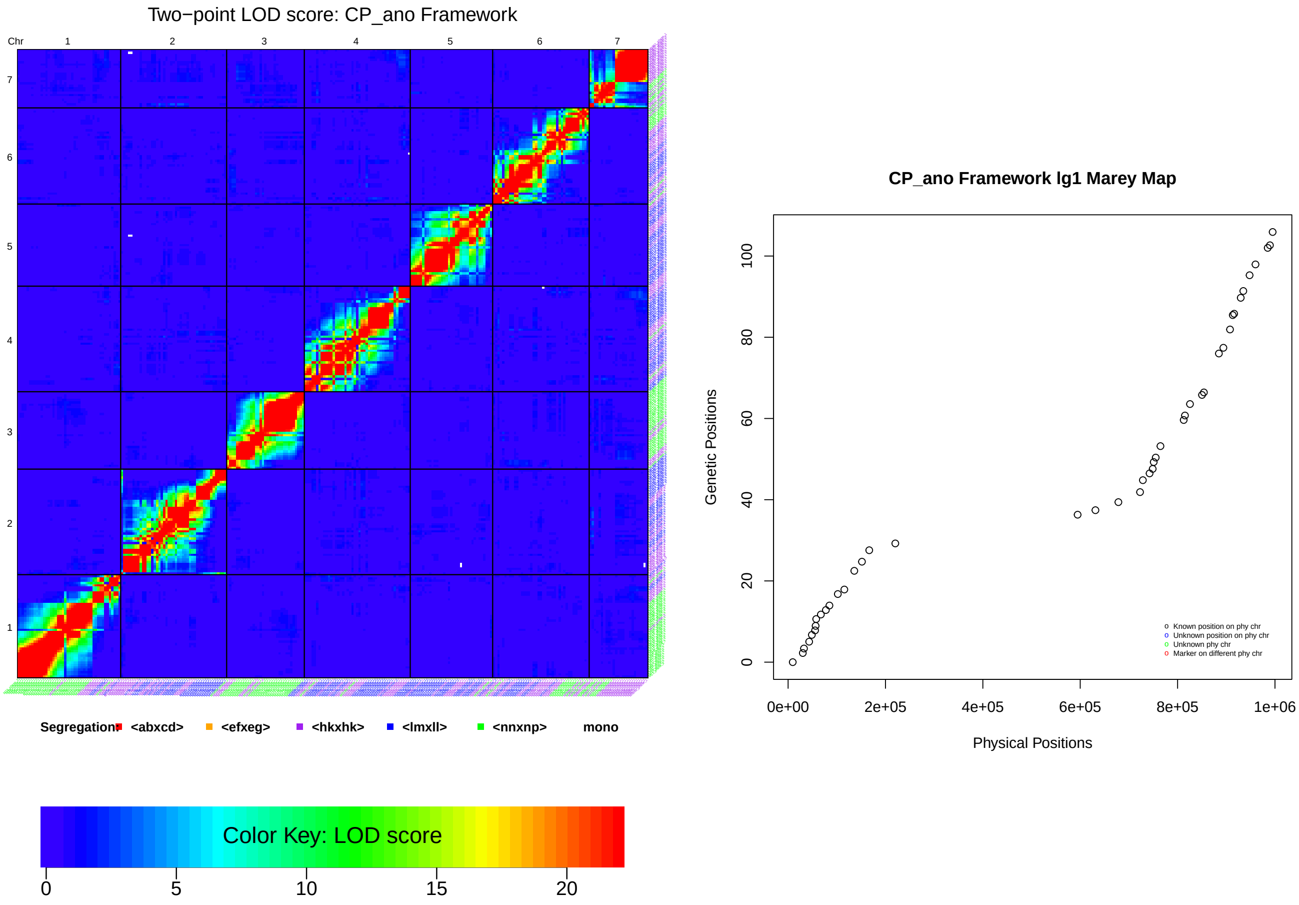

Left panel: pairwise 2-pt linkage LOD between markers of the framework map (all chromosomes). Right panel: Marey map of the framework map for chromosome 1. X-axis: relative physical positions (normalized to 0-10^6) of the markers given in the phyMap file. Y-axis: genetic position of the markers.
