# Supplementary material for: SeSAM: software for automatic construction of order-robust linkage maps
Source: BMC Bioinformatics. 2022 Nov 19;23:499. doi: 10.1186/s12859-022-05045-7 (PMC9675223; doi:10.1186/s12859-022-05045-7)
Supplement: Supplementary file 2 — Additional file 2: Reference User Manual of SeSAM, including a quick-start tutorial. [file 12859_2022_5045_MOESM2_ESM.pdf]

SeSAM
Seriation-based Suite for Automatic genetic Mapping
User Manual

Adrien Vidal & Matthieu Falque

October 26, 2022

Contents

1 Introduction **2**

2 Input and output data file formats **2**

2.1 Marker Segregation Data . . . 3
2.1.1 Population types . . . 3
2.1.2 Genotypes and encoding . . . 3
2.1.3 Segregation data dataframe . . . 4
2.1.4 .raw format . . . 5
2.1.5 .loc format . . . 7
2.1.6 .gen format . . . 11
2.1.7 Segregation Data Input/Output wrappers . . . 13
2.2 Map Data . . . 16
2.3 Marker Quality Data . . . 19
2.4 Marker Placement Information Data . . . 20
2.5 Log files . . . 21
2.6 Mapping Saves (RData) . . . 21
2.7 SpellMapTools Session Cache . . . 21

3 Graphical Outputs **22**

3.1 Data quality visualization . . . 22
3.2 Assignment Barplot . . . 22
3.3 2-pt linkage heatmaps . . . 22
3.4 Marey maps . . . 22
3.5 Graphical genotypes . . . 22
3.6 Colinearity plots . . . 22

4 Standard pipeline for automated map construction **23**

4.0.1 Marker information data dataframe . . . 26
4.0.2 Mapping status . . . 28
4.1 Launching an automated map construction: `autoMap()` . . . 28
4.2 Stopping and restarting . . . 33
4.3 Main pipeline functions . . . 33
4.3.1 Data reading and pre-processing: `loadData()` . . . 33
4.3.2 Initializing a SpellMapTools Session: `SPELL_cast()` . . . 36
4.3.3 Drawing seed markers: `generateSeeds()` . . . 37
4.3.4 Scaffold construction: `buildScaffold()` . . . 38
4.3.5 Assigning markers to linkage groups: `assignment()` . . . 41
4.3.6 Building frameworks from scaffolds: `buildFramework()` . . . 43

4.3.7 Placing all remaining markers on the framework: `placement()` 45

5 Creating *de novo* Linkage Groups **47**

6 Filtering outlier individuals with high crossover counts **51**

7 Simulating segregation data sets **55**
7.1 Simulating a genetic map 56
7.2 Simulating segregation data 57
7.3 Complete Simulation 59

8 Consensus map construction **60**

9 List of parameters and default values **63**

10 Further algorithmic details **64**
10.1 Population pedigrees 65
10.2 Marker segregation distortion 65
10.3 Estimation of recombination fractions per meiosis 65
10.4 Computation of genetic distances from recombination fractions: distance functions 66
10.5 Specifics of cross-pollinated populations 66
10.6 Management of putative genotyping errors 69
10.6.1 Twins 69
10.6.2 Singletons 70

11 Basic functions for manual analyses **70**
11.1 2pt analysis 70
11.2 EM multipoint analysis 72

12 Debugging tools **73**

13 Quick-Start tutorial **74**

1 Introduction

The sesam project was initiated when the authors had to construct genetic maps with large numbers of markers. Using the current available tools, the amount of human time required was such that they decided to develop a fully automated pipeline able to handle many markers. This pipeline can handle most common types of biparental mapping populations, including progenies obtained from crossing heterozygous parents. In addition to the automatic procedure, the `SeSAM` package includes a toolkit with a number of lower-level functions whereby one can analyze linkage and compare marker orders to build maps manually, convert segregation data to and from various formats, assess the quality of data and maps through graphcal outputs, ***simulate*** many types of segregation data sets, or compute consensus maps from several different maps. As data sets succeeded one another, the software was regularly updated to extend its domain of use and its robustness to issues associated with different particular situations. Your feedback is welcome to further extend this process.

2 Input and output data file formats

The following file formats are used throughout the `SeSAM` package for input and output data.

2.1 Marker Segregation Data

Marker segregation data files contain the genotypes of a set of markers in the mapping population. Several formats may be used to represent marker segregation data in `SeSAM`, depending on the genetic structure of the mapping panel.

2.1.1 Population types

Knowing the crossing scheme used to generate the mapping population (population type) is necessary to interpret any segregation data. This package uses a system of generic codes to identify the population types although it also accepts some commonly used codes as valid synonyms. The following table lists the population types supported for genetic mapping in `SeSAM`:

Population	Type	Supported by `SeSAM`
Haploid / Doubled Haploid	HAP	HAP
Successive Selfings (RIL self)	Fx	x = 2, 3 or >4
Intermating + Successive Selfings (IRIL self)	IxFy	any x, y>5
Successive Sib Matings (RIL sib)	Fxb	x>5
Intermating + Successive Sib Matings (IRIL sib)	IxFyb	any x, y>5
Backcross	BCx	BC1
Cross-Polinated	CP	CP

Table 1: Mappable Population Types

Furthermore, a number of synonyms will be automatically translated to a population type code understood by `SeSAM`.

Synonym (case insensitive)	Population Type
`haploid, doubled haploid, h, dh`	HAP
`bc, backcross, f2 backcross`	BC1
`f2 intercross`	F2
`RI`x	Fx
`ril self, ri self`	Fx as `SeSAM_par("ri_self")`
`ril sib, ri sib`	Fxb as `SeSAM_par("ri_sib")`
`iril self, iri self`	IxFy as `SeSAM_par("iri_self")`
`iril sib, iri sib`	IxFyb as `SeSAM_par("iri_sib")`
`unphased f2 backcross, unphased f2 intercross`	CP
`outbred, cross-pollinated`	CP

Table 2: Population Type Synonyms

2.1.2 Genotypes and encoding

Segregation data files may represent alleles in one of several ways in `SeSAM`, so that it can be used with raw data files formatted for other softwares. It is thus compatible with MapMaker or CarthaGene (`.raw` files) and JoinMap (`.loc` files), but it can also handle a new format (`.gen`) that we specified to be easier to use with SNP data. `SeSAM` includes functions allowing to read and write such data files, so all conversions to and from any of these formats is very easy to perform.

Genotypes are represented by a pair of characters representing the two alleles of diploid individuals. Any character may be used, for instance letters representing nucleotides at SNP loci. In `SeSAM`, the genotypes indicate the segregation of alleles in offsprings descending from a cross between two parental genotypes, so the offspring genotypes must be combinations of characters that are possible given the parental genotypes. For instance, with `cc` and `ct` as parental genotypes, the possible genotypes for offsprings are `cc`, `ct` and `tc`. Character order in offspring genotypes does not matter (thus `ct` and `tc` are equivalent) but in the case

of CP populations, the character order in the *parental* genotypes is used to indicate segregation phase (see section 10.5 page 66 for further details about segregation phases).

Encoding uses specific characters to represent segregation in a simplified manner without having to compare offspring genotypes to parental genotypes. `SeSAM` uses two systems of encoding. "Single-letter" encoding is a commonly used system and is suited for all population types except Cross-Pollinated (CP). It uses the `'A'`, `'B'`, `'H'`, `'C'`, `'D'` and `'-'` characters. `'C'` and `'D'` codes are used for dominant markers.

Code	Description	Genotype
A	Homozygous for first allele (c)	cc
B	Homozygous for second allele (t)	tt
H	Heterozygous	ct or tc
C	Not homozygous for first allele (c)	ct or tc or tt
D	Not homozygous for second allele (t)	ct or tc or cc
-	Unknown	-

Table 3: Letter encoding codes and what they represent.

For Cross-Pollinated populations in particular, `SeSAM` uses the system of hexadecimal codes used in the CarthaGene software (de Givry et al., 2004), thus ensuring data compatibility with that software. In this system, each value represents either a specific segregation of parental alleles or a set of possible segregations. In the latter case, the hexadecimal code is equal to the sum of the hexadecimal codes of the different possible segregations. Encoded CP genotypes thus embed parental haplotype information (see Table below).

Code	Letter Synonym	Combines	Possible Genotypes
1	A	1	$F_0 \vert M_0$
2		2	$F_0 \vert M_1$
3		1, 2	$F_0 \vert M_0,\ F_0 \vert M_1$
4		4	$F_1 \vert M_0$
5		1, 4	$F_0 \vert M_0,\ F_1 \vert M_0$
6	H	2, 4	$F_0 \vert M_1,\ F_1 \vert M_0$
7	D	1, 2, 4	$F_0 \vert M_0,\ F_0 \vert M_1,\ F_1 \vert M_0$
8	B	1	$F_1 \vert M_1$
9		1, 8	$F_0 \vert M_0,\ F_1 \vert M_1$
a		2, 8	$F_0 \vert M_1,\ F_1 \vert M_1$
b		1, 2, 8	$F_0 \vert M_0,\ F_0 \vert M_1,\ F_1 \vert M_1$
c		4, 8	$F_1 \vert M_0,\ F_1 \vert M_1$
d		1, 4, 8	$F_0 \vert M_0,\ F_1 \vert M_0,\ F_1 \vert M_1$
e	C	2, 4, 8	$F_0 \vert M_1,\ F_1 \vert M_0,\ F_1 \vert M_1$
f	-	1, 2, 4, 8	$F_0 \vert M_0,\ F_0 \vert M_1,\ F_1 \vert M_0,\ F_1 \vert M_1$

Table 4: Hexadecimal codes representing the segregation of parental alleles.

2.1.3 Segregation data dataframe

All segregation data file reading functions output a dataframe we call "segregation data dataframe". The variables of this dataframe are:

- **mrkName:** The unique identifier of the marker.
- **refName:** A non-unique identifier used to group duplicates in the case of CP populations (10.5).
- **p1:** Genotype of the population's first parent for this marker.
- **p2:** Genotype of the population's second parent for this marker.

- **genotype:** Genotypes of the individuals of the population for this marker. It is a string of length $2N$ for a population of N individuals where every two consecutive character represents a genotype.
- **segType:** Segregation type of this marker in CP populations (10.5).
- **phase:** Phase code identifying the parental allelic configuration at this marker in CP populations.
- **encoding:** Encoding of the segregations of the individuals of the population for this marker. It is a string of length N for a population of N individuals.

Furthermore, the following attributes may be attached to this dataframe (see `? attributes`):

- **popName:** Name of the population.
- **typePop:** Population type (2.1.1) of the population.
- **indivNames:** Names of the individuals of the population in the same order as the genotype and encoding strings.
- **nbCO:** Counts of the number of crossovers for the individuals ; this attribute is attached by `computeCOPerIndiv` (6).
- **genotype:** This table contains valid values for variables `genotype`, `p1` and `p2`.
- **encoded:** This table contains valid values for the `encoding` variable.
- **locified:** The characters used for the genotypes match joinMap's specification for genotypes. Use for the purpose of writing .loc files.
- **encodingType:** "letters" or "hexadecimal". Which encoding (2.1.2) format is in use in this table.

2.1.4 .raw format

The `.raw` format was first used in the software MapMaker (Lander et al., 1987) and has then been widely used in other mapping softwares (e.g. CarthaGene (de Givry et al., 2004)). It represents one marker per line with a string of one encoding character (letter or hexadecimal) for each individual. It must contain two header lines. The first header should be `data type` followed by the population type or a suitable synonym. The second header should contain, in this order, the number of individuals, the number of markers, and the number of QTLs (not used in `SeSAM`). Optionally, this may be followed by `'symbols'` followed by any number of `X=Y` statements. These statements are aliases used in the rest of the file to be translated into encoding characters. For example, `'0=A 1=B 2=H'` means "`0` is read as `A`, `1` read as `B` and `2` read as `H`". Each marker entry is marked with a '*' character followed by the marker's name, a separating character, and the string of encoding sequence, whose length must be the same for all markers. The marker names must all be unique, must start with a letter character, but can then include any non-blank character (although even blank characters may be used if the default separator is changed in the readRaw() function).

Example .raw file

```
data type F7
45 4 0 symbols A=A B=B H=- -=-

*mrk1 BABA-BBBBBBBBAAABABBBABBBBABAABAAAABBBBBBBBBA
*mrk2 BABAHBBBBBBBBAAABABBBABBBBAB-ABAAAABBBBBBBBBA
*mrk3 AAAABBBBABB-BABBBBABBABBABHBABAABHH-ABBBBBBAA
*mrk4 AAAABBBBABBABABBBBABBABBABHBABAABHHBABBBBBBA-
```

The dedicated function for reading `.raw` files is `readRaw()`. The following example shows a simple use of this function:
(CAUTION copying text from a pdf file to a R console may introduce additional characters like spaces)

```
library(SeSAM)
loadExample("testData")
segData <- readRaw(segDataFileRaw)
```

This function outputs a segregation data dataframe (2.1.3).

readRaw

```
readRaw(
  filename, typePop=NULL, popName=NULL, indivNames=NULL, sep=""
)
```

Description:

Reads a .raw segregation file and loads it as a segregation dataframe (2.1.3).

Arguments:

filename Path of the .raw segregation file to read.
typePop Population type code (2.1.1) or suitable synonym. Overwrites the population type possibly read in the file header.
popName Name of the population. Attached as attribute to the segregation data dataframe.
indivNames Vector of unique individual names. Attached as attribute to the outputted dataframe.
sep Separator character between marker names and encoding sequence. Defaults to any number of white space characters.

Value:

Segregation Data dataframe (2.1.3) with encoding variable but no genotype variables. Optional attributes attached.

The dedicated function for writing `.raw` files is `writeRaw()`.

writeRaw

```
writeRaw(
  segData, filename, typePop=NULL,
  naChar=SeSAM_par("naChar"),
  nb_cpu=SeSAM_par("nb_cpu"), aliases=NULL, sep="\t",
  verbose=FALSE, writose=FALSE, logFile=NULL
)
```

Description:

Writes a .raw segregation file from a segregation data dataframe (2.1.3).

Arguments:

segData Segregation data (2.1.3) or marker information (4.0.1) dataframe containing the segregation data to write. If the dataframe contains an empty encoding variable, the encoding will be computed from the parents and offspring genotypes first.

filename Path of the .raw segregation file to write.

typePop Population type code (2.1.1) or suitable synonym. Overwrites the dataframe's population type.

naChar Character representing missing data in genotypes. Only used when encoding the genotypes.

nb_cpu Number of CPUs useable by the parallelized processes (encoding).

aliases Named character vector of alliases to apply to written file. The values of the vector are the characters being replaced by the names of the vector. e.g. c("0"="A", "1"="H") => " symbols 0=A 1=H".

sep Separator character between marker names and encoding sequence. Defaults to tabulation.

verbose Write information in the standard output.

writose Write information in a file.

logFile File for writose outputs.

Value:

Path of the written file.

File_Output:

.raw text file as per the 'filename' argument.
.log text file as per the 'logFile' argument.

2.1.5 .loc format

The `.loc` format follows the format specifications from the joinMap (Stam, 1993) documentation. ';' marks comments. The file must begin with four header lines with the following flags:

- `name`: population's name.
- `popt`: population type. The joinMap population type codes can be understood by `SeSAM`. The nomenclature is the same as for `.raw` files (see 2.1.1)
- `nloc`: number of markers (loci).
- `nind`: number of individuals in the population.

Following the header are the marker entries. This section is formatted differently for CP populations than for the others. The marker names must all be unique. The end of marker entry is denoted with a `'individual names :'` line after which each non-empty line is an individual's name in the order in which their segregations are given for each marker. The individual names must all be unique as well.

For each marker in a non-CP population:

- one line with the name of the marker.
- any number of lines with the marker's encoding using characters `'a'`, `'b'`, `'h'`, `'c'`, and `'d'` as per letter encoding but in lower case and `'-'`, `'.'`, `'u'` to signify unknown segregations. The total number of encoding characters must equal the number of individuals (`nind`).

Example Non-CP .loc file

```
name=example_non-CP
popt=RI7
nloc=5
nind=45

data type F7
45 4827 0 symbols A=A B=B H=- -=-

mrk1 ; 1
   baba- bbbbb bbbaa ababb babbb babaa baaaa bbbbb bbbba

mrk2 ; 2
   babah bbbbb bbbaa ababb babbb bab-a baaaa bbbbb bbbba

mrk3 ; 3
   aaaab bbbab b-bab bbbab babba bhbab aabhh -abbb bbbaa

mrk4 ; 4
   aaaab bbbab babab bbbab babba bhbab aabhh babbb bbba-

individual names:
plant1
plant2
plant3
[...]
plant45
```

For each marker in a CP population:

- one line with the name of the marker followed by its segregation type. The segregation type may be one of `'<abxcd>'`, `'<efxeg>'`, `'<hkxhk>'`, `'<lmxll>'` or `'<nnxnp>'`. Bracketed phase informations after this are ignored by `SeSAM`.
- any number of lines with the marker's genotype in pairs of two characters using the set of characters from the segregation type. **CAUTION: each such line with marker's genotypes MUST begin with one or two spaces before the first genotype.**

Example CP .loc file

```
name = example_CP
popt = CP
nloc = 4
nind = 20

mrk1    <nnxnp>   ; 1
   np np nn nn np  nn nn nn nn np  nn nn np nn np  nn np nn np nn

mrk2    <abxcd>   ; 2
   bc bc ad bd ac  bd bd bd bd bd  ad bd ac bd ac  bd bc ad bc bd

mrk3    <lmxll>   ; 3
   ll ll ml ll ml  ll ll ll ll ll  ml ll ml ll ml  ll ml ml ll ll

mrk4    <efxeg>   ; 4
   eg eg fe ee eg  ee ee ee ee ee  fe ee fg ee fg  ee fe fe eg ee

individual names:
plant1
plant2
plant3
[...]
plant20
```

The dedicated function for reading `.loc` files is `readLoc()`. The following example shows a simple use of this function:
(CAUTION copying text from a pdf file to a R console may introduce additional characters like spaces)

```
library(SeSAM)
loadExample("testData")
segData <- readLoc(segDataFileLoc)
```

This function outputs a segregation data dataframe (2.1.3).

readLoc

```
readLoc(
  filename, typePop=NULL, popName=NULL, indivNames=NULL,
  naChar=SeSAM_par("naChar")
)
```

Description:

Reads a .loc segregation file and loads it as a segregation dataframe (2.1.3).

Arguments:

filename Path of the .raw segregation file to read.
typePop Population type code (2.1.1) or suitable synonym. Overwrites the population type read in the file header.
popName Name of the population. Attached as attribute to the segregation data dataframe.
indivNames Vector of unique individual names. Overwrites inividual names from the file.
naChar Character used to represent missing data in genotypes.

Value:

Segregation Data dataframe (2.1.3) with encoding variable (non−CP) or genotype, p1 and p2 variables (CP). Optional attributes attached.

The dedicated function for writing `.loc` files is `writeLoc()`. Note that, in the case of CP populations, the arbitrary characters used to represent offspring genotypes in the input dataframe will be replaced by the characters commonly used for each marker's segregation type (e.g. `'hh'`, `'hk'`, `'nn'` etc...).

writeLoc

```
writeLoc(
  segData, filename, typePop=NULL, popName=NULL, indivNames=NULL,
  naChar=SeSAM_par("naChar"), nb_cpu=SeSAM_par("nb_cpu"),
  verbose=FALSE, writose=FALSE, logFile=NULL
)
```

Description:

Writes a .loc segregation file from a segregation data dataframe (2.1.3).

Arguments:

segData Segregation data (2.1.3) or marker information (4.0.1) dataframe containing the segregation data to write. If encoding is needed and the dataframe contains an empty encoding variable, the encoding will be computed from the parents and offspring genotypes first.
filename Path of the .raw segregation file to read/write.
typePop Population type code (2.1.1) or suitable synonym. Overwrites the dataframe's population type.
popName Name of the population. Overwrites the dataframe's attached popName.
indivNames Vector of the individual names. Should not contain duplicates. Overwrites the attached attribute of the inputted dataframe.
naChar Character representing missing data in genotypes. Only used when encoding the genotypes.
nb_cpu Number of CPUs useable by the parallelized processes (encoding).
verbose Write information in the standard output.
writose Write information in a file.
logFile File for writose outputs.

Value:

Path of the written file.

File_Output:

.loc text file as per the 'filename' argument.
.log text file as per the 'logFile' argument.

2.1.6 .gen format

The `.gen` format is a tabulated format for parent and offspring diploid genotypes. Each row represents a marker. The first two columns hold marker names, the second and third columns are for the parents and every subsequent column represents an individual of the mapping population. The table may have a header begining with a cell separator character containing the individual names.

Example .gen file

```
       p1    p2    plant1  plant2  plant3  plant4  plant5  plant6
mrk1   AA    AC    AC      AC      AA      AA      AC      AA
mrk2   AT    CG    TC      TC      AG      TG      AC      TC
mrk3   CT    TT    TT      TT      TC      TT      TC      TT
mrk4   AT    AC    AC      AC      TA      AA      AC      AA
```

The dedicated function for reading `.gen` files is `readGen()`. The following example shows a simple use of this function:
(CAUTION copying text from a pdf file to a R console may introduce additional characters like spaces)

```r
library(SeSAM)
loadExample("testData")
segData <- readGen(segDataFileGen, typePop="BC1")
```

This function outputs a segregation data dataframe (2.1.3).

readGen

```r
readGen(
  filename, typePop=NULL, popName=NULL, indivNames=NULL,
  naChar=SeSAM_par("naChar"), sep=""
)
```

Description:

Reads a .gen segregation file and loads it as a segregation dataframe (2.1.3).

Arguments:

filename Path of the .gen segregation file to read.
typePop Population type code (2.1.1) or suitable synonym. Used to verify the validity of the segregations within the population. attaches as an attribute to the outputted dataframe.
popName Name of the population. Attached as attribute to the segregation data dataframe.
indivNames Vector of unique individual names. Attached as attribute to the outputted dataframe.
naChar Character used to represent missing data in genotypes.
sep Separator character between marker names and genotypes. Defaults to any number of white space characters.

Value:

Segregation Data dataframe (2.1.3). Optional attributes attached.

The dedicated function for writing `.gen` files is `writeGen()`.

writeGen

```
writeGen(
  segData, filename, naChar=SeSAM_par("naChar"), sep="\t",
  verbose=FALSE, writose=FALSE, logFile=NULL
)
```

Description:

Writes a .gen segregation file from a segregation data dataframe (2.1.3).

Arguments:

segData Segregation data (2.1.3) or marker information (4.0.1) dataframe containing the segregation data to write.
filename Path of the .gen segregation file to write.
naChar Character representing missing data in genotypes. Only used when encoding the genotypes.
sep Separator character between marker names and encoding sequence. Defaults to tabulation.
verbose Write information in the standard output.
writose Write information in a file.
logFile File for writose outputs.

Value:

Path of the written file.

File_Output:

.gen text file as per the 'filename' argument.
.log text file as per the 'logFile' argument.

2.1.7 Segregation Data Input/Output wrappers

The following two functions are wrappers for reading and writing the various segregation data file formats presented above.

readSegData

```
readSegData(
  filename, format=NULL, typePop=NULL, popName=NULL, indivNames=NULL,
  naChar=SeSAM_par("naChar"), sep=""
)
```

Description:

Reads a segregation data file (several possible formats) and loads it as a segregation dataframe (2.1.3).

Arguments:

filename Path of the segregation file to read. The format of the file is determined from this filename's extention.
format One of 'raw', 'loc', 'gen'. Format of the file to read. Overwrites the automatically detected file extension in 'filename'.
typePop Population type code (2.1.1) or suitable synonym. Overwrites the file's population type.
popName Name of the population. Attached as attribute to the segregation data dataframe.
indivNames Vector of the individual names. Should not contain duplicates. Overwrites any individual names read in the file.
naChar Character used to represent missing data in genotypes.
sep Separator character between marker names and encoding sequence. Defaults to any number of white space characters.

Value:

Segregation Data dataframe (2.1.3) with encoding variable but no genotype variables. Optional attributes attached.

writeSegData

```
writeSegData <- function(
  segData, filename, format=NULL, typePop=NULL,
  popName=NULL, indivNames=NULL, naChar=SeSAM\_par("naChar"),
  nb_cpu=SeSAM\_par("nb_cpu"),
  raw\_aliases=NULL, sep="\t",
  verbose=FALSE, writose=FALSE, logFile=NULL
)
```

Description:

Writes a segregation file from a segregation data dataframe (2.1.3).

Arguments:

segData Segregation data (2.1.3) or marker information (4.0.1) dataframe containing the segregation data to write. If encoding is needed and the dataframe contains an empty encoding variable, the encoding will be computed from the parents and offspring genotypes first.
filename Path of the segregation file to write.
format One of 'raw', 'loc', 'gen'. Format of the file to write. Overwrites the automatically detected file extension in 'filename'.
typePop Population type code (2.1.1) or suitable synonym. Overwrites the dataframe's population type.
popName Name of the population. Overwrites the dataframe's population name. Only used for .loc files.
indivNames Vector of the individual names. Should not contain duplicates. Overwrites the attached attribute of the inputted dataframe.
naChar Character used to represent missing data in genotypes.
nb_cpu Number of CPUs useable by the parallelized processes (encoding). Only used for .raw files and non−CP loc files.
raw_aliases Named character vector of alliases to apply to written .raw file. The values of the vector are the characters being replaced by the names of the vector. e.g c("0"="A", "1"="H") => "symbols 0=A 1=H".
sep Separator character between marker names and encoding sequence (.raw) or between cells (.gen). Defaults to a tabulation.
verbose Write information in the standard output.
writose Write information in a file.
logFile File for writose outputs.

Value:

Path of the written file.

File_Output:

.raw, .loc, or .gen segregation text file as per the 'filename' and 'format' **arguments**.
.log text file as per the 'logFile' argument.

2.2 Map Data

Map files are tabulated text files representing physical or genetic maps. Map files may serve as input and output throughout several modules in the `SeSAM` package: for instance physical map files are used to draw seed markers on each chromosome to initiate the seriation process for mapping and to graphically compare genetic maps with physical maps (generating so-called Marey maps). Genetic maps may be used as input to inspect colinearity between different maps, or to compute graphical genotypes and inspect the distribution of COs along chromosomes.
Map files may contain 3 or 4 variables.

- Marker names.
- Chromosome/linkage group.
- Position. Either a physical position in base pairs or a genetic position in centiMorgan. Physical positions must be positive integer values.
- Phase code. An integer value between 1 and 4 representing which arrangement of parental haplotypes correspond to this map. See section 10.5 page 66 for more details about phase codes. This variable is only used for genetic maps of cross-pollinated (CP) populations.

The dataframes outputed by the following map reading functions are known as "map dataframes" and can be specifically "physical" or "genetic". The names of the variables are as follows:

- **mrkName**, **chr**, **pos:** For a map of an unspecified type.
- **mrkName**, **phyChr**, **phyPos:** For a physical map.
- **mrkName**, **genChr**, **genPos:** For a physical map.

A fourth "phase" variable exists in genetic maps of CP populations (10.5).

Example map file

```
mrk1    1    0.000000
mrk2    1    1.626136
mrk3    1    6.176255
mrk4    1    6.625850
mrk5    1    10.000000
mrk6    2    0.000000
mrk7    2    6.444596
mrk8    2    7.040902
mrk9    2    9.898561
mrk10   2    10.000000
```

The functions for reading map files are `readPhyMap` (for physical maps) and `readGenMap` (for genetic maps). The only difference between those two is the names of the variables in the outputted dataframe which match with the variable names in marker information dataframes (see section 4.0.1 page 26) and the verifications made to variables. Genetic maps may optionally have a `phase` variable. These functions also accept arguments allowing reading tabulated text files with a different arragement of variables.

readGenMap

```
readGenMap(
  filename, colId=1, colChr=2, colPos=3, colPha=NULL,
  header=FALSE, sep="", naChars="-"
)
```

Description:

Reads a map file and loads it as a genetic map dataframe (2.2).

Arguments:

filename Name of the file to read.
colId Number of the column to read the markers' names from.
colChr Number of the column to read the markers' chromosomes/linkage groups from.
colPos Number of the column to read the markers' genetic position from.
colPha Number of the column to read/write the markers' phase codes from.
header Whether the file has a header line.
sep Character used to separate the table's cells. Defaults to any number of white space characters.
naChars Characters that signify a missing **value**, to be replaced with NA.

Value:

A physical map dataframe.

readPhyMap

```
readPhyMap(
  filename, colId=1, colChr=2, colPos=3,
  header=FALSE, sep="", naChars="-"
)
```

Description:

Reads a map file and loads it as a physical map dataframe (2.2).

Arguments:

filename Name of the file to read.
colId Number of the column to read the markers' names from.
colChr Number of the column to read the markers' chromosomes/linkage groups from.
colPos Number of the column to read the markers' genetic position from.
header Whether the file has a header line.
sep Character used to separate the table's cells. Defaults to any number of white space characters.
naChars Characters that signify a missing **value**, to be replaced with NA.

Value:

A physical map dataframe.

Both genetic and physical maps may be written with `writeMap`.

writeMap

```
writeMap(
  map, filename, colId=1L, colChr=2L, colPos=3L, colPha=4L, sep='\t',
  header=FALSE, verbose=FALSE, writose=FALSE, logFile=NULL
)
```

Description:

Write map file from a map dataframe.

Arguments:

filename Map dataframe (genetic or physical) to write.
filename Name of the file to write.
colId Number of the column to read/write the markers' names from/to.
colChr Number of the column to read/write the markers' chromosomes/linkage groups from/to.
colPos Number of the column to read/write the markers' genetic position from/to.
colPha Number of the column to read/write the markers' phase codes from/to.
header Whether the file has a header line.
sep Character used to separate the table's cells. Defaults to a tabulation.
naChars Characters that signify a missing **value**, to be replaced with NA.
verbose Write information in the standard output.
writose Write information in a file.
logFile File for writose outputs.

Value:

Path of the written file.

File_Output:

.txt tabulated file.
.log text file as per the 'logFile' argument.

2.3 Marker Quality Data

Marker Quality files are tabulated files with two columns. Marker quality files are an optional input to `loadData()` and by extention `autoMap()` (see section 4.3.1 page 33 and section 4.1 page 28) in order to apply a filter based on any sort of quality rating when classifying markers in quality categories. Quality scores must be between 0 and 1. A normalization of the value used to rate quality may be needed.

- Marker names.
- Marker quality scores between 0 and 1.

Example marker quality file

```
mrk1     5.7
mrk2     7.1
mrk3     5.5
mrk4     9.9
mrk5     3.6
mrk6     8.9
mrk7     3.2
mrk8     0.5
mrk9     3.7
mrk10    9.5
```

readMrkQual

```
readMrkQual(filename, colId=1L, colSco=2L, header=FALSE, sep="")
```

Description:

Reads a tabulated file containing marker quality scores and loads it as a Marker Quality dataframe.

Arguments:

filename Name of the file to read.
colId Number of the column to read the markers' names from.
colSco Number of the column to read the markers' scores from.
header Whether the file has a header line.
sep Character used to separate the table's cells. Defaults to any number of white space characters.

Value:

A Marker Quality dataframe.

2.4 Marker Placement Information Data

Placement information files are tabulated files outputted near the end of the map building process (See section 4.3.7 page 45). They contain more detailed information about the status of each marker in the final map, compared to regular map files presented above. The variables of these files are:

- `mrkName`: Name of the markers.
- `genChr`: Linkage group.
- `genPos`: Genetic position of the marker on its linkage group's map.
- `status`: The marker's current status (4.0.2) in the mapping process.
- `region`: Shows the set of other statistically plausible ranks of the marker in the map, around its most likely interval. Only markers added in the placement stage of the mapping process may have an uncertainty on the interval of the framework they belong to. The `'+'` character represents the marker's most likely interval. Other possible adjacent intervals are separated by a `':'` and rated by the LOD

between the most likely interval and each other possible interval. For example, a marker with a region value of '2.5:+:1.6' is most likely placed in the central interval, but may also lie in the neighbouring left or right intervals with a LOD of 2.5 and 1.6 respectively.

- fw_left: In the case of a placed marker: name of the marker preceding its most likely position in the map's marker order.
- fw_right: In the case of a placed marker: name of the marker following its most likely position in the map's marker order.

2.5 Log files

Many functions in SeSAM may write information in a file marked with a .log extension according to their writose argument. High level functions such as autoMap() and simulateMap() direct the output of all called functions to a same .log file.

2.6 Mapping Saves (RData)

The autoMap() function outputs RData files at several steps of the pipeline. These files contain objects from autoMap()'s internal environment in order to allow stopping at and resuming from those steps. These objects may be retrieved manually using load(). Exploring the mrkInfo marker information data frame (see section 4.0.1 page 26) loaded from these files allows advanced users to analyze different steps of the mapping process in many aspects when outputted files and graphical outputs aren't enough.

2.7 SpellMapTools Session Cache

SpellMapTools is a C++ module in charge of the ML and EM computation work. When a SpellMapTools session is initialized (see section 4.3.2 page 36), some data are pre-computed and written in a cache directory to save time in further calculations. This cache must be conserved as long as a SpellMapTools session using this cache is in use.

In particular cases with very large data sets, one may wants to keep this cache and later on use the same SpellMapTools session for further mapping analysis. This is possible by giving back the relevant information to the session construction function as shown in the example below:
(CAUTION copying text from a pdf file to a R console may introduce additional characters like spaces)

```
library(SeSAM)
loadExample("testData")
spellSession <- SPELL_cast( segData=mrkInfo_after_loadData, dirOut="
    SeSAM_example_testData/spell_session" )
SPELL_saveSessionInfo( spellSession=spellSession, filename="SeSAM_
    example_testData/spell_session/sessionInfo.RData")
q()  # the session is now saved, you can exit R

R  # next R session where you want to re-use the previous SpellMapTools
    cache
library(SeSAM)
loadExample("testData")
spellSession <- SPELL_recallFromInfoSave( filename="SeSAM_example_
    testData/spell_session/sessionInfo.RData" )
#  you can now proceed with further mapping analyses.
#  for instance 2pt linkage analyses, scaffold construction, etc.
```

3 Graphical Outputs

SeSAM generates a number of graphs throughout the mapping process. If the user wants to generate such graphs outside the contexte of a SeSAM mapping process, the SeSAM library provides the corresponding functions listed below. All details about the use of these functions can be obtained with the R command '?'. The different graphs generated provide information at the following levels:

3.1 Data quality visualization

Several graphs are generated relative to the quality of the data (missing data per marker or per individual, segregation bias). The associated functions are:
mappingCategoriesAreaPlot()
mrkDistoHist()
mrkQualityHist()
mrkMissingDataHist()
indMissingDataHist()

3.2 Assignment Barplot

This graph indicates the numbers of markers successfully and unambiguously assigned to a each linkage group, and those which could not be assigned. The corresponding function is assignmentBarplot().

3.3 2-pt linkage heatmaps

These graphs are extremely useful to visually assess the quality of a map. They represent the strength of pairwise linkage in a matrix of markers, based on LOD scores and on genetic distance. The associated functions are:
heatmap2pt()
lod2ptHeatMap()
dist2ptHeatMap()
distAndLod2ptHeatMaps()

3.4 Marey maps

Marey maps represent the genetic position of markers as a function of their physical position. Its slope thus visually indicates the local recombination rate. Flat regions in such Marey maps typically indicate centromere positions in many species. The associated function is `mareyMap()`.

3.5 Graphical genotypes

These graphs represent the genotypes of the individuals of the population along the chromosomes. Each possible genotype is represented by a color so that any change in color along a chromosome indicates a crossover within that marker interval. In the case of CP population, each genotype is represented by a pair of colors each corresponding to a parental allele rather than representing all 16 possible genotypes by a separate color. These graphs are useful to visually inspect very close-by COs which may indicate genotyping errors. The associated function is graphicalGenotypes().

3.6 Colinearity plots

SeSAM also proposes several types of plots to visualize the level of colinearity between the genetic map and the physical map. This proved to be useful to investigate non-colinear regions, for (1) detecting inversions in a genome assembly, (2) detecting ordering errors in a genetic map, and (3) detecting true local non-colinearities between the reference genome used for the physical map and the couple of parents of the segregating population used for the genetic map. The associated functions are: colinearity()

colinearityMat()
bestColinearityOrders()
ladderGraphs()

4 Standard pipeline for automated map construction

The main feature of the `SeSAM` library is its automated map construction pipeline. It involves different steps including seriation, assignment, and placement algorithms. Overall, the pipeline successively proceeds with the four main steps below:

- **Scaffold**: For each chromosome, starting from one `seed` marker, a low-density map is build by seriation with the constraint of including sparsely and regularly spaced markers (at distances between `scaffDistRange[1]` and `scaffDistRange[2]` from each other) strongly linked with their neighbours. Scaffold maps thus include few markers, but their order is statistically extremely robust.

- **Assignment**: After scaffolding, each remaining marker is tested for linkage ith all scaffold markers, in order to assign that marker to the linkage group determined by one of the scaffolds.

- **Framework**: Framework maps are specified to represent the best compromise between marker density and statistical robustness of marker order. To build them, `SeSAM` will try to densify the scaffolds by adding more markers, thus making marker order slighty less robust but still statistically supported by a LOD threshold (`minMultipointLod`). The maximum number of markers included in this framework map will thus be limited by the size of the mapping population: the larger the population the more markers will be possible to include while keeping order robustness above the threshold.

- **Placement**: In this last step, `SeSAM` will place all remaining polymorphic markers one by one on the framework map, but without including them in that map. The mapping (placement) positions of all markers are used to produce a high-density (`Total`) map, but the order of this map is not statistically supported at a fine scale.

The rest of this section will provide more details about the different steps of the pipeline.

The procedures presented here apply for non-CP populations. For CP populations, which require phasing, a number of additional steps have been incorporated in the software. More information about methods to handle CP populations may be found in section 10.5 page 66.

Marker classification

Before mapping, all markers are classified into several sets depending on their usability in the different mapping steps:

- **Mappable**: The set of mappable markers contains all markers which are not monomorphic, do not have a hundred percent of missing data, and for which the segregation is determined (no missing parental genotype).

- **Strict**: The set of strict markers contains all mappable markers whose distortion, percentage of missing data and quality score (if provided) are within parametrable thresholds (see section 9 page 63).

- **Reduced**: The set of reduced markers is a set of non-redundant strict markers. To obtain this set, co-segregation groups are first built, and then the marker having the lowest number of missing data is selected in each group. Two markers are considered redundant (co-segregating) when their genotypes for all individuals are either known to be the same or may be the same (case of missing data in either marker).

Most of the mapping process uses only the reduced set of markers or, if reduction by redundancy is turned off, the strict set of markers. The rest of the markers are placed on the map at the end of the process, but are not considered for the determination of robust marker orders.

Linkage Group Seeds

The first step is to draw a number of `seed` markers for each linkage group as the starting point of the seriation process. Seeds are drawn from the reduced set of markers. Several seeds can be drawn in each linkage group to provide replicates of next step (scaffold), to avoid problems due to possible particular unlucky choices of seeds (e.g. seed very poorly linked to other markers of its linkage group). Chosing these seed markers requires to know at least one marker in each linkage group for each replicate. This information is strictly used for the drawing of seed markers, and will not be used in any further step of map construction, so the genetic map construction is blind to any previous knowledge for instance on physical mapping (this may be important e.g. to use genetic maps to detect errors in physical maps). The information used to chose the seed markers may come from one of two sources:

- Physical Map: As it is common today to have physical mapping (and/or sequencing) information, that information may be used as the starting point of the seriation algorithm. Although this physical information is, as stated previously, only used for drawing seed markers, physical chromosome assignments and map positions may be useful throughout the building process, to compare the newly produced genetic map with the physical map, thus providing a form of independent quality control. Such comparisons are visualized through so-called Marey maps (see section 3.4 page 22). Alternatively, in the absence of physical map, previous genetic maps may be used instead of physical maps to draw seed markers.

- Putative Linkage Groups: In the absence of physical map or previous genetic map, `SeSAM` can also implement a seriation algorithm to build linkage groups from randomly drawn seeds. This method is detailed in section 5 page 47.

The seed markers of different chromosomes are tested for linkage against each other to ensure that they are not cross-linked. If they are, new seed markers are drawn until no more cross-linkage is detected.

Scaffold map construction

The aim of this step is to provide a set of markers covering each linkage group with a sparse map of regularly spaced markers, so the order of this map is statistically extremely robust. For each seed marker drawn previously, a scaffold map is then constructed. Each of these constructions is independent from the others. The scaffold elongation procedure starts from the seed marker and adds strongly linked markers on either side of the scaffold alternatively (see figure 1 page 25). Specifically, markers are selected such that:

- They have the highest possible linkage (and in all cases a LOD > `minMappingLod`) to the marker at the current scaffold's extremity on the considered side.

- They lay within a range of distance to the extremity marker (greater than `scafDistRange[1]` and lower than `scafDistRange[2]`). Markers which are too close to a marker of the current scaffold are blacklisted from this particular construction.

- In the case of CP populations, further conditions and priorities are tested. More detail is provided in section 10.5 page 66.

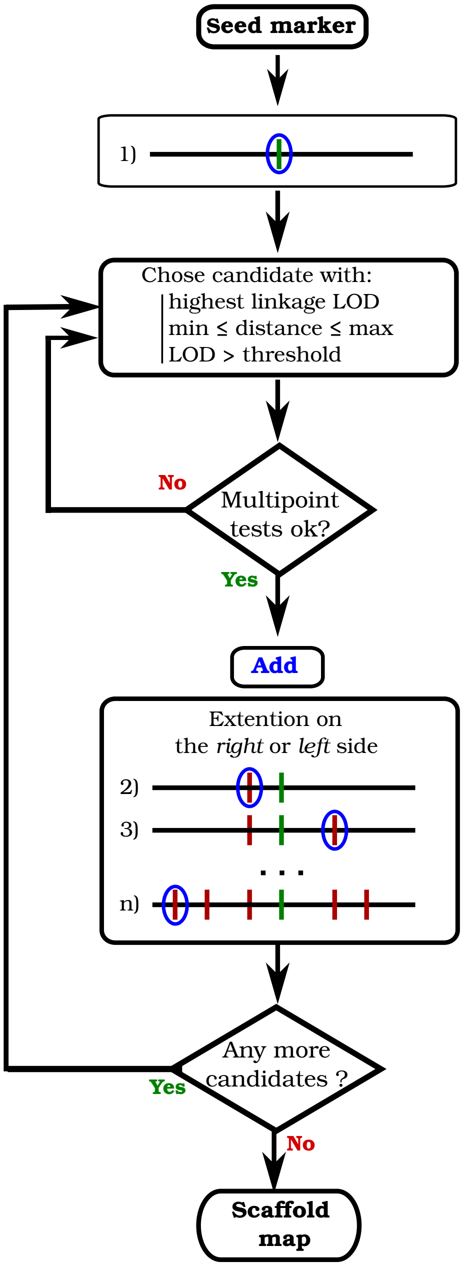

Figure 1: Scaffold building procedure.

The selected marker with the strongest linkage to one scaffold extremity becomes the candidate which must pass a number of quality tests before being added. If a test fails, the next candidate is tested until one passes all tests or there is no marker left. In this last case, extension on this side of the map is stopped. The tests that a candidate must pass are:

- "Continuity test": Checks that the distance between the candidate marker and the second last marker at the considered extremity of the scaffold is less than twice the maximum distance between two markers of the scaffold (`scafDistRange[2]`). This serves to ensure that a single anomalous linkage (e.g. due to segregation distortion) doesn't make the construction jump to markers that should not belong to the current group. This proved to be useful with some data sets...

- "Try test": Checks that the position at the extremity of the current scaffold is the best possible position for the candidate on the scaffold, and that it is significantly better than the second best one (LOD > `minMultipointLod`).
- "Locus specificity test": Only for backcross populations, based on conformity to expected two-locus marginal allele frequencies (p-value must be below `locusSpecificityPval`).
- "3-point distance test": Between the candidate, the extremity marker and the penultimate marker. Checks that the distance between the candidate and the penultimate marker is greater than the other two distances. This test is only used with CP populations and proved to be useful in some particular cases.

Once a scaffold map has been built from each seed, the replicates for each linkage group are sorted by number of markers. For each linkage group, the replicate with the highest number of markers is selected. All selected replicates are then filtered for cross-linkage, and the process is repeated until no cross-linkage is detected. `SeSAM` considers that there is cross-link if at least one of the two following conditions is met: (1) a marker of a group is linked to a marker of another group with LOD > `maxCrossLod`, or (2) the difference between the highest linkage LOD of a marker with another marker of its group, and the highest linkage LOD of this marker with a marker of another group, is lower than `minDeltaLod`.

Assigning markers to linkage groups

All remaining markers are then tested for linkage against each of the constructed scaffold maps to determine to which linkage group they belong. This is done by measuring the maximum 2-point linkage LOD of each marker against each linkage group scaffold. Markers having no linkage to any group with LOD > `minMappingLod` are considered unassigned and those which are linked to two different groups are considered in conflict (using the same both criteria and associated thresholds `maxCrossLod` and `minDeltaLod` as in the previsous section). Neiter unassigned nor conflicting markers are considered for the rest of the construction.

Building frameworks from scaffolds

This step aims to densify the map, starting from the scaffold, to obtain the most dense yet statistically robust possible map. For each linkage group, all assigned markers of the reduced set are considered sequentially to attempt to find their position on the map. Markers are added to the map only if (1) they are linked to the group with LOD > `minMappingLod` and (2) the best interval of placement is robust at a LOD threshold of `minMultipointLod`. Each new candidate is tested on the map that includes all previously added markers. Finally, the statistical robustness of the order between all markers of the complete framework is checked (using the `minMultipointLod` threshold). This procedure also rejects markers which would inflate the genetic length of the map too much (threshold: `maxFrameworkExpansion`).

Placing all remaining markers on the framework

This step aims to find the best possible position for each remaining marker of the mappable set, including those that were considered for the framework map but could not be added. Each marker is thus placed independently of the others on the framework map, which means that the order between two placed markers is not statistically supported, but this allows to place extremely large numbers of markers whatever the population size.

4.0.1 Marker information data dataframe

Most functions involved in the mapping process use a structure called a "marker information dataframe" as either an input, output or both. This structure combines marker segregation dataframes (2.1.3), genetic maps and physical maps (2.2) and contains additional variables relevant to the mapping process. All functions which take a segregation data dataframe as input accept marker information dataframes as well.

- **mrkName:** The unique identifier of the marker.
- **refName:** A non-unique identifier used to group duplicates in the case of CP populations (10.5).

- **p1:** Genotype of the population's first parent for this marker.
- **p2:** Genotype of the population's second parent for this marker.
- **genotype:** Genotypes of the individuals of the population for this marker. It is a string of length $2N$ for a population of N individuals where every two consecutive character represents a genotype.
- **segType:** Segregation type of this marker in CP populations (10.5).
- **phase:** Phase code identifying the parental configuration of this marker in CP populations.
- **encoding:** Encoding of the segregations of the individuals of the population for this marker. It is a string of length $2N$ for a population of N individuals.
- **status:** The marker's current status (4.0.2) in the mapping process.
- **phyChr:** The marker's physical chromosome.
- **phyPos:** The marker's physical position.
- **genChr:** The marker's genetic chromosome.
- **genPos:** The marker's genetic position.
- **numValid:** Number of valid (not missing data) individual genotypes the marker.
- **distortion:** The marker's segregation distortion percentage (10.2). Positive value between 0 and 100.
- **quality:** The marker's quality score. Positive value between 0 and 1.
- **mappable:** Whether the marker is useable for the map.
- **strict:** Whether the marker is useable for 'scaffold' (4.3.4) and 'framework' (4.3.6) maps construction.
- **reduced:** Whether the marker is part of the reduced set of markers.
- **twin:** Whether the marker is a twin (Identical markers exist in the dataset).
- **region:** String to represent placement (4.3.7) uncertainty on flanking intervals.

Furthermore, the following attributes may be attached to this dataframe:

- **popName:** Name of the population.
- **typePop:** Population type (2.1.1) of the population.
- **indivNames:** Names of the individuals of the population in the same order as the genotype and encoding strings.
- **nbCO:** Counts of the number of crossovers for the individuals attached by `computeCOPerIndiv` (6).
- **genotype:** This table contains valid values for variables `genotype`, `p1` and `p2`.
- **encoded:** This table contains valid values for the `encoding` variable.
- **locified:** The characters used for the genotypes matches joinMap's specification for genotypes. Use for the purpose of writing .loc files.
- **encodingType:** "letters" or "hexadecimal". Which encoding (2.1.2) format is in use in this table.

4.0.2 Mapping status

Markers are given a mapping status during the mapping process to identify whether they have been succesfully assigned and at which stage, if any, they have been added to the map. The possible statuses are:

- **none:** Yet unused.
- **not mappable:** Monomorphic, or no valid data.
- **seed:** Linkage group seed marker.
- **scaffold:** Part of the scaffold map.
- **assigned:** Assigned to a linkage group but not part of any map yet.
- **unassigned:** Couldn't be assigned to any linkage group.
- **conflict:** Linked to more than one linkage group. Assignment couldn't be determined.
- **framework:** Part of the framework map.
- **placed:** Part of the total map (`Full`) but not of scaffold or framework maps.

4.1 Launching an automated map construction: `autoMap()`

The `autoMap()` function runs the entire algorithm presented above. It functions as a pipeline that runs each major step within a function and is able to stop and resume at the end of each step through the use of `.RData` saves containing objects from its internal environment. The `writose` and `graphose` arguments modulate the amount of information about the advance of the process, that will be sent respectively to the standard output and the log file, and to separate graphics files. Note that even if both of these arguments are set to `FALSE`, `autoMap` needs to write files on disk for its cache and step save files. The structure of the output directory can be found in figure 2 page 29. The `autoMap()` function has many arguments, most of them optional and inherited from sub-functions to allow to control and tweak every part of the process. A number of those arguments default to a call to the `SeSAM_par` (see section 9 page 63) function and thus will take the values previously modified by the user, if any.

In the following example, we use the `testData` files that come with the `SeSAM` library to run the mapping procedure. This outputs a marker information dataframe (see section 4.0.1 page 26) and writes a number of files in the `SeSAM_example_testData` folder.
(CAUTION copying text from a pdf file to a R console may introduce additional characters like spaces)

```
library(SeSAM)
loadExample("testData")
SeSAM_par(nb_rep=2) # 2 scaffold replicates (2 seeds) per chromosome.

mrkInfo <- autoMap(
  mapName="testData",
  dirOut="SeSAM_example_testData",
  segDataFile=segDataFileRaw,
  phyMapFile=phyMapFile
)
```

```
<dirOut>/
├── SeSAM_<mapName>.log ........................................ Default path ① to ⑦ Ⓦ
├── <mapName>_distortion.pdf ................................................ ① Ⓖ
├── <mapName>_quality.pdf ................................ If marker quality file ① Ⓖ
├── <mapName>_missing_per_markers.pdf ....................................... ① Ⓖ
├── <mapName>_missing_per_individuals.pdf ................................... ① Ⓖ
├── <mapName>_marker_categories.pdf ......................................... ① Ⓖ
├── Rdata_saves/
│   ├── save_[1]_Loading.RData ................................................ ①
│   ├── save_[2]_Seeding.RData ................................................ ②
│   ├── save_[3]_Scaffolding.RData ............................................ ③
│   ├── save_[4]_Assignment_1.RData ........................................... ④
│   ├── save_[5]_Frameworking.RData ........................................... ⑤
│   ├── save_[6]_Assignment_2.RData ........................................... ⑥
│   └── save_[7]_Placement.RData .............................................. ⑦
├── spell_session/
│   ├── SPELL_<mapName>_<date-and-time>.ped
│   ├── SPELL_<mapName>_<date-and-time>.raw
│   ├── spell-map-tools-<md5>
│   └── sessionInfo.RData
├── putative_LGs/ ................................... If usePutativeLGs ② Ⓦ or Ⓖ
│   ├── <mapName>_putative_LGs.txt ............................................ Ⓦ
│   ├── <mapName>_putative_LGs_scaffold.txt ................................... Ⓦ
│   ├── <mapName>_putative_LGs_assignment_counts.pdf .......................... Ⓖ
│   └── <mapName>_putative_LGs_iterations.pdf ................................. Ⓖ
├── <mapName>_seeds.txt ..................................................... ② Ⓦ
├── <mapName>_seeds_2pt_linkage.pdf ................................ 2 pages ② Ⓖ
├── scaffold_reps/ ..................................................... ③ Ⓦ or Ⓖ
│   ├── lg<lgName>/ ...................................................... Per LG.
│   │   ├── <mapName>_scaffold_genMap_LG<lgName>_rep<repNum>.txt ...... 1 file/rep Ⓦ
│   │   ├── <mapName>_scaffold_2pt_linkage_LG<lgName>_rep<repNum>.pdf .... Per rep; 2 pages Ⓖ
│   │   ├── <mapName>_scaffold_marey_map_LG<lgName>_rep<repNum>.pdf ...... Per rep Ⓖ
│   │   ├── <mapName>_scaffold_ladder_graph_LG<lgName>_rep<repNum>.pdf ... Per rep Ⓖ
│   │   ├── <mapName>_scaffold_positions_LG<lgName>_rep<repNum>.pdf ...... Per rep Ⓖ
│   │   └── <mapName>_scaffold_LODs_LG<lgName>_rep<repNum>.pdf ........... Per rep; Ⓖ
│   └── <mapName>_scaffold_top_reps_2pt_linkage.pdf ................... 2 pages Ⓖ
├── <mapName>_scaffold_genMap.txt ........................................... ③ Ⓦ
├── <mapName>_genotyping_errors.txt ....................................... ③ ⑤ Ⓦ
├── <mapName>_scaffold_2pt_linkage.pdf ............................ 2 page/LG ③ Ⓖ
├── <mapName>_scaffold_marey_maps.pdf ............................. 1 page/LG ③ Ⓖ
├── <mapName>_scaffold_ladder_graph.pdf ........................... 1 page/LG ③ Ⓖ
├── <mapName>_scaffold_positions.pdf .............................. 1 page/LG ③ Ⓖ
├── <mapName>_scaffold_LODs.pdf ............................................. ③ Ⓖ
├── <mapName>_assignment_counts.pdf ....................................... ④ ⑥ Ⓖ
├── framework_LGs/ ..................................................... ⑤ Ⓦ or Ⓖ
│   ├── <mapName>_framework_genMap_LG<lgName>.txt ..................... Per LG Ⓦ
│   ├── <mapName>_framework_2pt_linkage_LG<lgName>.pdf ....... Per LG; 2 pages Ⓖ
│   ├── <mapName>_framework_marey_map_LG<lgName>.pdf .................. Per LG Ⓖ
│   ├── <mapName>_framework_ladder_graph_LG<lgName>.pdf ...... Per LG; 1 page/LG Ⓖ
│   └── <mapName>_framework_positions_LG<lgName>.pdf ......... Per LG; 1 page/LG Ⓖ
└── ...
```

Figure 2: Directory structure of the outputs of the autoMap() function. ①...⑦ = Created/Updated at this step. Ⓦ = If writose. Ⓖ = If graphose.

```
<dirOut>/
├── ...
├── <mapName>_framework_genMap.txt ........................ Step 5; If writose
├── <mapName>_framework_graphical_genotypes.pdf ............ 1 page/LG ⑤ Ⓖ
├── <mapName>_framework_2pt_linkage_separate.pdf ........... 2 page/LG ⑤ Ⓖ
├── <mapName>_framework_2pt_linkage_merged.pdf ............. 2 pages ⑤ Ⓖ
├── <mapName>_framework_marey_maps.pdf ..................... 1 page per LG ⑤ Ⓖ
├── <mapName>_framework_ladder_graph.pdf ................... 1 page per LG ⑤ Ⓖ
├── <mapName>_framework_positions.pdf ...................... 1 page per LG ⑤ Ⓖ
├── <mapName>_framework_filtered_2pt_linkage_merged.pdf .... ⑤ Ⓖ
├── <mapName>_framework_filtered_marey_maps.pdf ............ 1 page per LG ⑤ Ⓖ
├── <mapName>_framework_filtered_ladder_graph .............. 1 page per LG ⑤ Ⓖ
├── <mapName>_framework_filtered_positions.pdf ............. 1 page per LG ⑤ Ⓖ
├── total_LGs/ ............................................. ⑦ Ⓦ or Ⓖ
│   ├── <mapName>_total_genMap_LG<lgName>.txt .............. For each LG Ⓦ
│   ├── <mapName>_total_2pt_linkage_LG<lgName>.pdf ......... For each LG; 2 pages Ⓖ
│   ├── <mapName>_total_marey_map_LG<lgName>.pdf ........... For each LG Ⓖ
│   ├── <mapName>_total_ladder_graph_LG<lgName>.pdf ........ Per repeat; 1 page/LG Ⓖ
│   └── <mapName>_total_positions_LG<lgName>.pdf ........... Per repeat; 1 page/LG Ⓖ
├── <mapName>_total_genMap.txt ............................. ⑦ Ⓦ
├── <mapName>_total_detailed_info.txt ...................... ⑦ Ⓦ
├── <mapName>_total_2pt_linkage_separate.pdf ............... 2 pages ⑦ Ⓖ
├── <mapName>_total_2pt_linkage_merged.pdf ................. ⑦ Ⓖ
├── <mapName>_total_marey_maps.pdf ......................... 1 page per LG ⑦ Ⓖ
├── <mapName>_total_ladder_graph.pdf ....................... 1 page per LG ⑦ Ⓖ
├── <mapName>_total_positions.pdf .......................... 1 page per LG ⑦ Ⓖ
└── <mapName>_marker_information.txt ....................... Ⓦ
```

Figure 2: Directory structure of the outputs of the autoMap() function. ①...⑦ = Created/Updated at this step. Ⓦ = If writose. Ⓖ = If graphose.

autoMap

```
autoMap( mapName, dirIn=NULL, dirOut=NULL, typePop=NULL,
  restartStep=NULL, stopStep=NULL,
  segDataFile=NULL, segData_format=NULL, segData_sep="",
  genotype_naChar = SeSAM_par("naChar"), indivNames=NULL,
  phyMapFile =NULL, phyMap_colId =1L, phyMap_colChr =2L,
  phyMap_colPos=3L, phyMap_header =FALSE,
  phyMap_sep ="", phyMap_naChars="-", usePutativeLGs=FALSE,
  mrkQualFile=NULL, mrkQual_colId=1L, mrkQual_colSco=2L,
  mrkQual_header=FALSE, mrkQual_sep="",
  minQualityScore=SeSAM_par("minQualityScore"),
  maxMissingdata=SeSAM_par("maxMissingdata"),
  maxDistoMappable=SeSAM_par("maxDistoMappable"),
  maxDistoStrict=SeSAM_par("maxDistoStrict"),
  nb_rep=SeSAM_par("nb_rep"),
  mappingFunction=SeSAM_par("mappingFunction"),
  scaffDistRange=SeSAM_par("scaffDistRange"),
  minDeltaLod=SeSAM_par("minDeltaLod"),
  minMappingLod=SeSAM_par("minMappingLod"),
  maxCrossLod=SeSAM_par("maxCrossLod"),
  minMultipointLod=SeSAM_par("minMultipointLod"),
```

```
  locusSpecificityPval=SeSAM_par("locusSpecificityPval"),
  maxFrameworkExpansion=SeSAM_par("maxFrameworkExpansion"),
  nb_cpu=SeSAM_par("nb_cpu"),
  maxSessionSize=SeSAM_par("maxSessionSize"),
  mrkPhaseSep= SeSAM_par("mrkPhaseSep"),
  lgFragmentSep=SeSAM_par("lgFragmentSep"),
  reduceByRedundancy=TRUE, CPDeployPhases=TRUE,
  verbose=TRUE, writose=TRUE, graphose=TRUE, logFile=NULL,
  seedRNG=1, safe2ptMat=FALSE )
```

Description:

Main function for \sesam' genetic map construction process.

Arguments:

mapName Population name for the map. Used to automatically identify files. From the following patterns, files are detected in the input directory (working directory or dirIn path):
<mapName>_segData.<raw|loc|gen>: Segregation data file. File extention (.raw, .loc or .gen) determines file type (see 2.1.7). Pattern ignored if segDataFile is given.
<mapName>_phyMap.<ext>: Physical map file. Ignored if phyMapFile is given. File extention is ignored.
<mapName>_mrkQual.txt: Marker quality file. Ignored if mrkQualFile is given. File extention is ignored.
dirIn Base directory path for input files. If given, input file search (see mapName) is made in this directory. Otherwise, input files are searched by default the current working directory. If dirIn is given, paths given through segDataFile, phyMapFile and mrkQualFile are interpreted as relative paths from this dirIn.
dirOut Base directory for the output files. When starting the pipeline from step 1, a default output directory is created in the input directory. When restarting the pipeline from step 2 or further, dirOut must be specified and must correspond to a directory containing a "rdata_save " directory with the save file from the previous step. See the File Output section for the structure of this output directory.
typePop A population type code (see (2.1.1). Needs to be given for segregation data file formats which do not contain that information. Overrides the population type from the segregation data file if there is one.
restartStep Name or number of the step to restart the pipeline from. Requires the RData file outputted by the previous step (see **description**).
stopStep Name or number of the step to stop the pipeline at.
segDataFile Name of the segregation data file. May be a file of one of the following formats : \ itemize{
".raw": Encoded genotype segregation data file. See 2.1.7.
".loc": joinMap segregation data file format. See 2.1.7.
".gen": Genotype table file format. See 2.1.7.
segData_... (segData_format, segData_sep, segData_naChar, segData_indivNames) Additional parameters passed down to the segregation data file reading function (see 2.1.7).
genotype_naChar Default character used to represent NA/missing data in genotype files.
indivNames Vector of the names of the individuals of the population. Attached as an attribute to the returned mrkInfo dataframe.
phyMapFile Name of the physical map file. See 2.2.
phyMap_... (phyMap_colId, phyMap_colChr, phyMap_colPos, phyMap_header, phyMap_sep, phyMap_naChars): Additional parameters passed down to the physical map file reading

function (2.2).

usePutativeLGs Behaviour concerning the construction of putative linkage groups before drawing seed markers. 0: Never construct putative LGs, will only build one LG if no physical map data is given. 1: Construct putative LGs only if no physical map data is given. 2: Always construct putative LGs, only using any given physical map data for graphical comparisons.

mrkQualFile Name of the marker quality file file (2.3.

mrkQual_... (mrkQual_colId, mrkQual_colSco, mrkQual_header, mrkQual_sep) Additional parameters passed down to the marker quality file reading function (2.3).

minQualityScore Quality score threshold for marker quality.

maxMissingdata Tolerated percentage of missing data to consider a marker as "strict".

maxDistoMappable Tolerated percentage of distortion from expected allele frequencies to integrate the marker in the map.

maxDistoStrict Tolerated percentage of distortion from expected allele frequencies to consider a marker to be useable for the mapping steps producing statistically supported map orders (scaffold, framework).

nb_rep Number of seed markers to draw from each chromosome. One scaffold map is built for each before filtering and selecting to assemble the complete scaffold.

mappingFunction "haldane" or "kosambi". Mapping function for genetic distances (10.4.

scaffDistRange Length 2 numeric vector (min and max). Genetic distance range between two consecutive markers for scaffold construction.

minDeltaLod Minimum 2pt−LOD difference for choosing between two linkage hypotheses (e.g. linkage to two different chromosomes).

minMappingLod Minimum 2pt linkage to consider adding a marker to a map.

maxCrossLod Minimum two point LOD to consider a linkage to an alternative chromosome (cross−linkage) significative.

minMultipointLod Minimum multipoint LOD score to consider a marker order significantly better than another.

locusSpecificityPval p−**value** Threshold to below which the locus specifity test will be rejected. Test only used in backcross populations.

maxFrameworkExpansion Maximum ratio of map length expansion for a candidate marker to be integrated in the framework map.

genotypingErrorDetection Whether to filter out data that appear to be genotyping error in the framework map.

nb_cpu Number of CPUs useable by the parallelized processes.

maxSessionSize Maximum number of markers that should be loaded in a single SpellMapTools bayesian network session.

mrkPhaseSep Character/String used to separate marker name from their phase. Example " mrk1_phase_1". Visible in output files.

lgFragmentSep String of characters to denote a smaller fragment of the same reference linkage groups.

reduceByRedundancy TRUE/FALSE. Whether or not to reduce the set of markers for steps 2 to 5 from the set of "strict" markers to a reduced set by filtering redundant markers (no cross −over inbetween). Allows to reduce the computational and memory load for large sets of markers.

CPDeployPhases TRUE/FALSE. Whether or not to deploy the required marker phases for genotyped CP populations. Disable for debugging purposes only.

verbose TRUE/FALSE. Write information in the standard output and display progress bars for lengthy processes.

writose TRUE/FALSE. Write information in a log file and output text documents for the results of the different steps (See File Output section below).

graphose TRUE/FALSE. Write pdf chart files for the results of the different steps (See File Output section below).

logFile Name of the file in which to output the writose information. Defaults to "<dirOut>/SeSAM_<mapName>.log".
seedRNG Integer to set the seed of R's Random Number Generator. If NULL, lets R set a pseudo−random seed as normal.

Value:

Marker Information Dataframe (see section 4.0.1 page 26).

File_Output:
See the file outputs on figure 2 page 29.

4.2 Stopping and restarting

The `autoMap` function is able to stop or resume at any of the major steps previously presented. To resume, the `dirOut` argument must be the path of a folder, as presented in figure 2 page 29 which contains, in the `rdata_saves` directory, the relevant RData save file (2.6) for the step preceding the step to restart with. For instance, in order to resume at the "scaffold" step. The save file for the "seeding" step must be present. Furthermore, when resuming at any step which requires the main SpellMapTools session, `autoMap` will attempt to recover the cache (2.7) left by previous runs in this `dirOut` directory using the `./spell_session/sessionInfo.RData` file.

4.3 Main pipeline functions

The `autoMap` pipeline procedure is subdivided into functions for each of its main steps which may be used manually one after the other for a more controlled map building approach.

4.3.1 Data reading and pre-processing: `loadData()`

The `loadData` function reads the various file inputs needed for mapping or other tasks and performs some analyses in order to classify markers for the next steps. With the 'mapName' only, this function will detect and read relevant files within the working directory or the `dirIn` directory. Alternatively, each file path may be specified individually with either their relative paths to `dirIn` if specified or their absolute paths. This function may load the following types of files:

- Segregation Data File: of any of the formats presented in section 2.1 page 3. Depending on the format to read, some additional arguments may need to be provided to be passed on to the relevant reader function.
- Physical Map File: as presented in section 2.2 page 16. This file is optional. It is only used to draw seed markers from different chromosomes, and to graphically compare *a posteriori* the genetic map produced with the physical map through a Marey map graph.
- Genetic Map File: as presented in section 2.2 page 16. This file is optional. It is not used for linkage mapping, but only with the function `consensusMap()` to compute a consensus map from different existing genetic maps.
- Marker Quality File: as presented in section 2.3 page 19. This file is optional.

The following operations are, by default, made on the inputted data by `loadData`:

- Matching loci between segregation and map data.
- Measuring the amount of missing data per marker and per individual.

- Detecting monomorphic markers.
- Calculating an index of the segregation distortion (see section 10.2 page 65) for each marker.
- Finding the set of `mappable` markers from the above values (basically all markers that are polymorphic).
- Finding the set of `strict` markers from the above values (based on thresholds for marker quality, segregation distortion, and missing data).
- Finding the set of `reduced` markers by filtering redundancy (exact co-segregation) out of the `strict` set.
- In the case of CP, deploying the necessary alternative parental haplotypes for phasing. More information about marker phasing in CP population may be found in section 10.5 page 66.
- In the case of genotype (e.g. AA, AC, TT) data, encoding each segregation data point as a single character in the column "encoding" (see below).

This function outputs a data frame called Marker Information (mrkInfo, see section 4.0.1 page 26), which combines segregation data, map data and the status of each marker in the mapping procedure. Every other main step function gets this data frame as an argument, modifies it, and returns it. This mrkInfo data frame thus accumulates information throughout the entire `autoMap` procedure.

In the following example, we load and process the `testData` files that come with the `SeSAM` library. This outputs a marker information dataframe (see section 4.0.1 page 26) and writes a number of files in the `testData_SeSAM_run` folder.
(CAUTION copying text from a pdf file to a R console may introduce additional characters like spaces)

```
library(SeSAM)
loadExample("testData")

mrkInfo <- loadData(
  mapName="testData",
  dirOut="SeSAM_example_testData",
  segDataFile=segDataFileRaw,
  phyMapFile=phyMapFile,
  writose=TRUE, graphose=TRUE
)
```

This example and the examples that follow for each function of the mapping procedure should be run one after the other.

loadData

```
loadData( mapName, dirIn=NULL, typePop=NULL,
  segDataFile=NULL, segData_format=NULL, segData_sep="",
  genotype_naChar=SeSAM_par("naChar"), indivNames=NULL,
  phyMapFile=NULL, phyMap_colId=1L, phyMap_colChr=2L,
  phyMap_colPos=3L, phyMap_header=FALSE,
  phyMap_sep="", phyMap_naChars='-',
  genMapFile=NULL, genMap_colId=1L, genMap_colChr=2L,
  genMap_colPos=3L, genMap_colPha=NULL, genMap_header=FALSE,
  genMap_sep="", genMap_naChars='-',
  mrkQualFile=NULL, mrkQual_colId=1L, mrkQual_colSco=2L,
  mrkQual_header =FALSE, mrkQual_sep ="",
  minQualityScore=SeSAM_par("minQualityScore"),
  maxMissingdata=SeSAM_par("maxMissingdata"),
  maxDistoMappable=SeSAM_par("maxDistoMappable"),
```

```
  maxDistoStrict=SeSAM_par("maxDistoStrict"),
  mrkPhaseSep= SeSAM_par("mrkPhaseSep"),
  computeDistortion=TRUE, nb_cpu=SeSAM_par("nb_cpu"),
  reduceByRedundancy=TRUE, CPDeployPhases=TRUE,
  verbose=TRUE, writose=FALSE, graphose=FALSE,
  logFile=NULL, dirOut=getwd()
)
```

Description:

Load, analyse, integrate and classify data in a Marker Information dataframe (see section 4.0.1 page 26). Deploy other phases for CP populations

Arguments:

mapName Population name for the map. Used to automatically identify files. From the following patterns, files are detected in the input directory (working directory or dirIn path):
<mapName>_segData.<raw|loc|gen>: Segregation data file. File extention (.raw, .loc or .gen) determines file type (see 2.1.7). Pattern ignored if segDataFile is given.
<mapName>_phyMap.<ext>: Physical map file. Ignored if phyMapFile is given. File extention is ignored.
<mapName>_mrkQual.txt: Marker quality file. Ignored if mrkQualFile is given. File extention is ignored.
dirIn Base directory path for input files. If given, input file search (see mapName) is made in this directory. Otherwise, input files are searched by default the current working directory. If dirIn is given, paths given through segDataFile, phyMapFile and mrkQualFile are interpreted as relative paths from this dirIn.
typePop A population type code (see (2.1.1). Needs to be given for segregation data file formats which do not contain that information. Overides the population type from the segregation data file if there is one.
segDataFile Name of the segregation data file. May be a file of one of the .raw, .loc, or .gen formats. The format is detected by the file extention but can be overriden by segData_format.
segData_... (segData_format, segData_sep, segData_naChar, segData_indivNames) Additional parameters passed down to the segregation data file reading function (see 2.1.7).
indivNames Vector of the names of the individuals of the population. Attached as an attribute to the returned mrkInfo dataframe.
genotype_naChar Default character used to represent NA/missing data in genotype files.
phyMapFile Name of the physical map file. See 2.2.
phyMap_... (phyMap_colId, phyMap_colChr, phyMap_colPos, phyMap_header, phyMap_sep, phyMap_naChars): Additional parameters passed down to the physical map file reading function (2.2).
genMapFile Name of the genetic map file. See 2.2.
genMap_... (genMap_colId, genMap_colChr, genMap_colPos, genMap_header, genMap_sep, genMap_naChars): Additional parameters passed down to the genetic map file reading function (2.2).
mrkQualFile Name of the marker quality file file (2.3.
mrkQual_... (mrkQual_colId, mrkQual_colSco, mrkQual_header, mrkQual_sep) Additional parameters passed down to the marker quality file reading function (2.3).
minQualityScore Quality score threshold for marker quality.
maxMissingdata Tolerated percentage of missing data to consider a marker as "strict".
maxDistoMappable Tolerated percentage of distortion from expected allele frequencies to integrate the marker in the map.

maxDistoStrict Tolerated percentage of distortion from expected allele frequencies to consider a marker to be useable for the mapping steps producing statistically supported map orders (scaffold, framework).
maxSessionSize Maximum number of markers that should be loaded in a single SpellMapTools bayesian network session.
mappingFunction "haldane" or "kosambi". Mapping function for genetic distances (10.4.
nb_cpu Number of CPUs useable by the parallelized processes.
mrkPhaseSep Character/String used to separate marker name from their phase. Example " mrk1_phase_1". Visible in output files.
computeDistortion TRUE/FALSE, Whether or not to compute marker distortion.
reduceByRedundancy TRUE/FALSE. Whether or not to reduce the set of markers for steps 2 to 5 from the set of "strict" markers to a reduced set by filtering redundant markers (no cross −over inbetween). Allows to reduce the computational and memory load for large sets of markers.
CPDeployPhases TRUE/FALSE. Whether or not to deploy the required marker phases for genotyped CP populations. Disable for debugging purposes only.
verbose TRUE/FALSE. Write information in the standard output and display progress bars for lengthy processes.
writose TRUE/FALSE. Write information in a log file and output text documents for the results of the different steps (See File Output section below).
graphose TRUE/FALSE. Write pdf chart files for the results of the different steps (See File Output section below).
logFile Name of the file in which to output the writose information. Defaults to "<dirOut>/SeSAM_<mapName>.log".
dirOut Base directory for the output files. Defaults to either dirIn or the working directory. Will be created even if writose and graphose are off for the purpose of writing the SpellMapTools bayesian network sessions' cache directories.

Value:

Marker Information Dataframe (see section 4.0.1 page 26).

File_Output:
<dirOut>/
SeSAM_<mapName>.log (if writose) Default log file.
<mapName>_distortion.pdf (if graphose) Histogram of the markers' distortion.
<mapName>_quality.pdf (if graphose and marker quality file) Histgram of the markers' quality scores.
<mapName>_missing_per_markers.pdf (if graphose) Histogram of the markers' missing data percentage.
<mapName>_missing_per_individuals.pdf (if graphose) Histogram of the individuals' missing data percentage.
<mapName>_marker_categories.pdf (if graphose) Concentric circles representing the proportions of each categories of markers.

See the file outputs in figure 2 page 29. for Step 1 except for the RData save file.

4.3.2 Initializing a SpellMapTools Session: `SPELL_cast()`

SpellMapTools is a module of `SeSAM` written in C++, which is in charge of the most computation-intensive parts of the mapping process, namely the maximum-likelihood (ML) and the Expectation-Maximization (EM) algorithms used to estimate recombination rates (see section 10.3 page 65). Thus, to perform any

of the subsequent mapping steps, the segregation data must first be loaded into a SpellMapTools session. This step performs some preliminary computations and write them into cache files. Note that all steps except `placement` only require the `reduced` set of markers to be loaded. For the `placement` procedure, the SpellMapTools session must contain all `mappable` markers to be analyzed in the considered batch, plus the framework map. This allows, in the case of large datasets, to only load the `reduced` set into a first session to use throughout most of the procedure, and then to create any number of new sessions to process the remaining markers in batches. This avoids to saturate the memory, whatever the number of markers to map.

The following example initializes a SpellMapTools session from the marker info dataframe created in the previous example (`mrkInfo_after_loadData`).
(CAUTION copying text from a pdf file to a R console may introduce additional characters like spaces)

```
library(SeSAM)
loadExample("testData")

spellSession <- SPELL_cast(
  segData=mrkInfo_after_loadData,
  dirOut="SeSAM_example_testData/spell_session"
)
```

4.3.3 Drawing seed markers: `generateSeeds()`

Drawing seeds is done through the `generateSeeds` function. This function will sample `nb_rep` seed markers from the reduced set of makers for each linkage group such that no seed marker has a linkage LOD to any seed marker of a linkage group other than its own larger than `maxCrossLod`.

The following example draws a set of seeds using the marker information dataframe (`mrkInfo_after_loadData`) and SpellMapTools session (`spellSession`) created in the previous steps.
(CAUTION copying text from a pdf file to a R console may introduce additional characters like spaces)

```
library(SeSAM)
loadExample("testData")
spellSession <- SPELL_cast(segData=mrkInfo_after_loadData, dirOut="SeSAM
   _example_testData/spell_session")

seeds_DF <- generateSeeds(
  mrkInfo=mrkInfo_after_loadData,
  spellSession=spellSession,
  dirOut="SeSAM_example_testData", nb_rep=2,
  verbose=TRUE, writose=TRUE, graphose=TRUE
)
```

generateSeeds

```
generateSeeds(
  mrkInfo, spellSession, mrkPool=NULL, typePop=NULL,
  nb_rep=SeSAM_par("nb_rep"), maxCrossLod=SeSAM_par("maxCrossLod"),
  mappingFunction=SeSAM_par("mappingFunction"), phasing=NULL,
  verbose=FALSE, writose=FALSE, graphose=FALSE, logFile=NULL,
  dirOut=getwd(), seedRNG=1
)
```

Description:

Generate a list of seed markers for scaffolding by drawing random markers from each physical chomosome without cross–link (significantly stronger linkage to their chromosome's seed markers than to those of others).

Arguments:

mrkInfo Marker Information dataframe (4.0.1).
spellSession a SpellMapTools object pointing to an active session.
mrkPool Set of marker names to define the pool of markers from which seeds can be drawn.
nb_rep Number of seed markers to draw from each chromosome.
maxCrossLod Minimum two point LOD to consider a linkage to an alternative chromosome (cross–linkage) significative.
mappingFunction "haldane" or "kosambi". Mapping function for genetic distances (10.4). Only used in this function for graphical outputs.
phasing TRUE/FALSE, whether or not the seeds have to be drawn from multiple possible phases (CP populations).
verbose TRUE/FALSE. Write information in the standard output.
writose TRUE/FALSE. Write information in a log file.
logFile Name of the file in which to output the writose information. Defaults to "<dirOut>/ SeSAM_<mapName>.log".
graphose TRUE/FALSE. Write LOD and genetic distance heatmap pdf charts for the seeds.
dirOut Base diractory for the output files. Defaults to the working directory.
seedRNG Value to set the seed of R's Random Number Generator. If NULL, lets R set a pseudo –random seed as normal.

Value:

Physical map dataframe (2.2) of the generated seed markers.

File_Output:
<dirOut>/
SeSAM_<mapName>.log (if writose) Default log file.
<mapName>_seeds.txt (if writose) Seed markers in a physical map format.
<mapName>_seeds_2pt_linkage.pdf (if graphose) pairwise two–point LOD and distance heatmaps of the markers drawn as seeds.

See the file outputs in figure 2 page 29. for Step 2 except for the RData save file.

4.3.4 Scaffold construction: `buildScaffold()`

The `buildScaffold` function constructs a scaffold map from a single seed marker using the seriation procedure described in Figure 1 page 25 and section 4 page 23.

The `buildMultiScaffold` function builds maps from each seed marker (corresponding to each replicate), checks (and filters) markers cross-linked with illegitimate chromosomes, and then for each chromosome the longest scaffold replicate is selected. The output is a single scaffold map for each chromosome.

The following example builds the scaffold map using the marker information dataframe (`mrkInfo_after_loadData`), SpellMapTools session (`spellSession`) and set of seeds (`seeds_DF`) created in the previous steps. This returns an updated marker information dataframe containing the constructed scaffold map (`mrkInfo`). (CAUTION copying text from a pdf file to a R console may introduce additional characters like spaces)

```r
library(SeSAM)
loadExample("testData")
spellSession <- SPELL_cast(segData=mrkInfo_after_loadData, dirOut="SeSAM
   _example_testData/spell_session")

mrkInfo <- buildMultiScaffold(
  seeds=seeds_DF,
  mrkInfo=mrkInfo_after_loadData,
  spellSession=spellSession,
  dirOut="SeSAM_example_testData",
  verbose=TRUE, writose=TRUE, graphose=TRUE
)
```

buildScaffold

```r
buildScaffold( seed, mrkInfo, spellSession,
  typePop=NULL, mrkPool=NULL,
  scaffDistRange=SeSAM_par("scaffDistRange"),
  minMappingLod=SeSAM_par("minMappingLod"),
  minMultipointLod=SeSAM_par("minMultipointLod"),
  locusSpecificityPval=SeSAM_par("locusSpecificityPval"),
  mappingFunction=SeSAM_par("mappingFunction"), phasing=NULL
)
```

Description:

Build a scaffold map by sequentially adding markers from a seed marker.

Arguments:

seed Marker name present in mrkInfo. Seed marker for the scaffold map building process.
mrkInfo Marker Information dataframe (4.0.1).
spellSession a SpellMapTools object pointing to an active session.
typePop Character scalar. A population type string (2.1.1). Defaults to mrkInfo's typePop attribute if any.
mrkPool Set of marker names to define the pool of markers from which candidate markers can be used to build the scaffold map.
scaffDistRange Length 2 numeric vector (min and max). Genetic distance range between two consecutive markers for scaffold construction.
minMappingLod Minimum 2pt linkage to consider adding a marker to a map.
minMultipointLod Minimum multipoint LOD score to consider a marker order significantly better than another.
locusSpecificityPval p−**value** Threshold to below which the locus specifity test will be rejected.
mappingFunction "haldane" or "kosambi". Mapping function for genetic distances (10.4).
phasing TRUE/FALSE, whether or not the seeds have to be drawn from multiple possible phases (CP populations).

Value:

genetic map dataframe (2.2) of the built scaffold map.

buildMultiScaffold

```
buildMultiScaffold( seeds, mrkInfo, spellSession,
  typePop=NULL, mrkPool=NULL,
  scaffDistRange=SeSAM_par("scaffDistRange"),
  minMappingLod=SeSAM_par("minMappingLod"),
  minDeltaLod=SeSAM_par("minDeltaLod"),
  minMultipointLod=SeSAM_par("minMultipointLod"),
  maxCrossLod=SeSAM_par("maxCrossLod"),
  locusSpecificityPval=SeSAM_par("locusSpecificityPval"),
  mappingFunction=SeSAM_par("mappingFunction"),
  mrkPhaseSep=SeSAM_par("mrkPhaseSep"),
  lgFragmentSep=SeSAM_par("lgFragmentSep"), phasing=NULL,
  verbose=FALSE, writose=FALSE, graphose=FALSE,
  logFile=NULL, dirOut=getwd()
)
```

Description:

Build a scaffold map for each seed marker and constitute the final scaffold map for each chromosome by selecting the longest replicates (if there were multiple seeds per chromosome to produce scaffold replicates) with filtered cross–linked markers.

Arguments:

seeds Vector of marker names or physical map dataframe (2.2) of the seed markers for scaffold map repetitions.
mrkInfo Marker Information dataframe (4.0.1).
spellSession a SpellMapTools object pointing to an active session.
typePop Character scalar. A population type string (2.1.1). Defaults to mrkInfo's typePop attribute if any.
mrkPool Set of marker names to define the pool of markers from which candidate markers can be used to build the scaffold map.
scaffDistRange Length 2 numeric vector (min and max). Genetic distance range between two consecutive markers for scaffold construction.
minMappingLod Minimum 2pt linkage to consider adding a marker to a map.
minDeltaLod Minimum 2pt–LOD difference for choosing between two linkage hypotheses (e.g. linkage to two different chromosomes).
minMultipointLod Minimum multipoint LOD score to consider a marker order significantly better than another.
maxCrossLod Minimum two point LOD to consider a linkage to an alternative chromosome (cross–linkage) significative.
locusSpecificityPval p–**value** Threshold to below which the locus specifity test will be rejected.
mappingFunction "haldane" or "kosambi". Mapping function for genetic distances (10.4).
mrkPhaseSep Character/String used to separate marker name from their phase. Example " mrk1_phase_1". Visible in output files.
lgFragmentSep String of characters to denote a smaller fragment of the same reference linkage groups.
phasing TRUE/FALSE, whether or not the seeds have to be drawn from multiple possible phases (CP populations).
verbose TRUE/FALSE. Write information in the standard output and display progress bars for lengthy processes.
writose TRUE/FALSE. Write information in a log file.

logFile Name of the file in which to output the writose information. Defaults to "<dirOut>/SeSAM_<mapName>.log".
graphose TRUE/FALSE. Write Marey graph and heatmap pdf files.
dirOut Base diractory for the output files. Defaults to the working directory.

Value:

Inputted marker information dataframe (4.0.1) with updated genetic chromosome, genetic position and mapping status variables. In the case of CP populations, the mapped markers are phased and the other phases are removed from the table.

File_Output:

<dirOut>/
SeSAM_<mapName>.log (if writose) Default log file.
scaffold_reps/ (if writose or graphose)
lg<chromosome>/ (per chromosome)
<mapName>_scaffold_genMap_LG<lgName>_rep<repNum>.txt (per repetition, if writose) Genetic map of the chromosome scaffold repetition.
<mapName>_scaffold_2pt_linkage_LG<lgName>_rep<repNum>.pdf (per repetition, if graphose) pairwise two–point LOD and distance heatmaps of the chromosome scaffold repetition.
<mapName>_scaffold_marey_map_LG<lgName>_rep<repNum>.pdf (per repetition, if graphose and physical file loaded) Marey map of the chromosome scaffold repetition.
<mapName>_scaffold_ladder_graph_LG<lgName>_rep<repNum>.pdf (per repetition, if graphose and physical file loaded) Ladder graph compairing physical and genetic marker orders for the chromosome scaffold repetition.
<mapName>_scaffold_positions_LG<lgName>_rep<repNum>.pdf (per repetition, if graphose) Positions graph of the chromosome scaffold repetition.
<mapName>_scaffold_LODs_LG<lgName>_rep<repNum>.pdf Boxplot of the neighbour to neighbour LODs of the chromosome scaffold repetition.
<mapName>_scaffold_top_reps_2pt_linkage.pdf (if graphose) pairwise two–point LOD and distance heatmaps of the longest scaffold repetitions per chromosome.
<mapName>_scaffold_genMap.txt (if writose) Genetic map of the complete scaffold map.
<mapName>_genotyping_errors.txt (if writose) Reports suspected genotyping errors.
<mapName>_scaffold_2pt_linkage.pdf (if graphose) pairwise two–point LOD and distance heatmaps of the complete scaffold map.
<mapName>_scaffold_marey_maps.pdf (if graphose and physical file loaded) Marey map of the complete scaffold map.
<mapName>_scaffold_ladder_graph.pdf (if graphose and physical file loaded) Ladder graph compairing physical and genetic marker orders for the scaffold.
<mapName>_scaffold_positions.pdf (if graphose) Positions graph of the scaffold.
<mapName>_scaffold_LODs.pdf (if graphose) Boxplot of the neighbour to neighbour LODs of the scaffold.

4.3.5 Assigning markers to linkage groups: `assignment()`

The `assignment` function is used after the scaffold maps are built to determine to which linkage groups the remaining markers belong. In the cases where there is a large number of markers in the dataset and loading them all at once into a single SpellMapTools session would require too much memory, then the markers that are not in the reduced set (and thus not necessary for the next step) can be assigned in several batches, after building the framework map. This function is also used then.

The following example assigns the markers from the scaffold map (`mrkInfo_after_buildMultiScaffold`) and the SpellMapTools session (`spellSession`) built at the previous steps. This returns an updated marker information dataframe containing the assignment information (`mrkInfo`).
(CAUTION copying text from a pdf file to a R console may introduce additional characters like spaces)

```
library(SeSAM)
loadExample("testData")
spellSession <- SPELL_cast(segData=mrkInfo_after_loadData, dirOut="SeSAM
   _example_testData/spell_session")

mrkInfo <- assignment(
  mrkInfo=mrkInfo_after_buildMultiScaffold,
  spellSession=spellSession,
  dirOut="SeSAM_example_testData",
  verbose=TRUE, writose=TRUE
)
```

assignment

```
assignment <- function(
  mrkInfo, spellSession, mrkPool=NULL, typePop=NULL, whichMap=c("
     framework", "scaffold"),
  minMappingLod=SeSAM_par("minMappingLod"), minDeltaLod=SeSAM_par("
     minDeltaLod"),
  phasing=NULL, verbose=FALSE, writose=FALSE, logFile=NULL, dirOut=
     getwd()
)
```

Description:

Assign markers to linkage groups based on buit scaffold (4.3.4) or framework (4.3.6) maps.

Arguments:

mrkInfo Marker Information dataframe (4.0.1).
spellSession a SpellMapTools object pointing to an active session.
typePop Character scalar. A population type string (2.1.1). Defaults to mrkInfo's typePop attribute if any.
mrkPool Set of marker names to define the pool of markers to assign.
whichMap Which map should be used as reference for assignments ("framework" includes "scaffold").
minMappingLod Minimum 2pt linkage to consider adding a marker to a map.
minDeltaLod Minimum 2pt−LOD difference for choosing between two linkage hypotheses (e.g. linkage to two different chromosomes).
phasing TRUE/FALSE, whether or not the seeds have to be drawn from multiple possible phases (CP populations).
verbose TRUE/FALSE. Write information in the standard output and display progress bars for lengthy processes.
writose TRUE/FALSE. Write information in a log file.
logFile Name of the file in which to output the writose information. Defaults to "<dirOut>/SeSAM_<mapName>.log".
dirOut Base diractory for the output files. Defaults to the working directory.

Value:

Inputted marker information dataframe (4.0.1) with updated genetic chromosome and mapping status variables.

4.3.6 Building frameworks from scaffolds: `buildFramework()`

This function will successively add markers to a scaffold, provided that the resulting marker order is still robust at the chosen multipoint LOD threshold, in order to build the framework map.

The following example builds the framework map from the scaffold map, assignments (information contained in `mrkInfo_after_assignment`), and SpellMapTools session (`spellSession`) already produced at the previous steps. It returns the framework map into a new version of the marker information dataframe (`mrkInfo`).
(CAUTION copying text from a pdf file to a R console may introduce additional characters like spaces)

```
library(SeSAM)
loadExample("testData")
spellSession <- SPELL_cast(segData=mrkInfo_after_loadData, dirOut="SeSAM
   _example_testData/spell_session")

mrkInfo <- buildFramework(
  mrkInfo=mrkInfo_after_assignment,
  spellSession=spellSession,
  dirOut="SeSAM_example_testData",
  verbose=TRUE, writose=TRUE, graphose=TRUE
)
```

buildFramework

```
buildFramework(
  mrkInfo, spellSession, typePop=NULL, mrkPool=NULL,
  mappingFunction=SeSAM_par("mappingFunction"),
  minMappingLod=SeSAM_par("minMappingLod"),
  minMultipointLod=SeSAM_par("minMultipointLod"),
  locusSpecificityPval=SeSAM_par("locusSpecificityPval"),
  maxFrameworkExpansion=SeSAM_par("maxFrameworkExpansion"),
  maxExtention=SeSAM_par("scaffDistRange")["min"],
  naChar=SeSAM_par("naChar"), nb_cpu=SeSAM_par("nb_cpu"),
  lgFragmentSep=SeSAM_par("lgFragmentSep"), phasing=NULL
  verbose=FALSE, writose=FALSE, graphose=FALSE,
  logFile=NULL, dirOut=getwd()
)
```

Description:

Build a dense and robust framework map by densifying the scaffold map (4.3.4) with assigned markers (4.3.5).

Arguments:

mrkInfo Marker Information dataframe (4.0.1).
spellSession a SpellMapTools object pointing to an active session.

typePop Character scalar. A population type string (2.1.1). Defaults to mrkInfo's typePop attribute if any.
mrkPool Set of marker names to define the pool of markers to assign.
mappingFunction "haldane" or "kosambi". Mapping function for genetic distances (10.4).
minMappingLod Minimum 2pt linkage to consider adding a marker to a map.
minMultipointLod Minimum multipoint LOD score to consider a marker order significantly better than another.
locusSpecificityPval p−**value** Threshold to below which the locus specifity test will be rejected.
maxFrameworkExpansion Map length expansion rate allowed per integrated marker in the framework map.
maxExtention Maximum distance from the extremities of the map to add markers that extend the map outside of the current extremities. Defaults to the maximum distance of the scaffold extention process.
naChar Default character used to represent NA/missing data in genotype files.
nb_cpu Number of CPUs useable by the parallelized processes (encoding).
lgFragmentSep String of characters to denote a smaller fragment of the same reference linkage groups.
phasing TRUE/FALSE, whether or not the seeds have to be drawn from multiple possible phases (CP populations).
verbose TRUE/FALSE. Write information in the standard output and display progress bars for lengthy processes.
writose TRUE/FALSE. Write information in a log file.
logFile Name of the file in which to output the writose information. Defaults to "<dirOut>/SeSAM_<mapName>.log".
graphose TRUE/FALSE. Write Marey graph and heatmap pdf files.
dirOut Base directory for the output files. Defaults to the working directory.

Value:

Inputted marker information dataframe (4.0.1) with updated genetic chromosome, genetic position and mapping status variables.

File_Output:
<dirOut>/
SeSAM_<mapName>.log (if writose) Default log file.
framework_LGs/ }{ (if writose or graphose)
<mapName>_framework_genMap_LG<lgName>.txt (if writose, per LG) Genetic map of the chromosome framework map.
<mapName>_framework_2pt_linkage_LG<lgName>.pdf (if graphose, per LG) pairwise two−point LOD and distance heatmaps of the chromosome framework map.
<mapName>_framework_marey_map_LG<lgName>.pdf (if graphose and physical file loaded, per LG) Marey map of the chromosome framework map.
<mapName>_framework_ladder_graph_LG<lgName>_rep<repNum>.pdf (per LG, if graphose and physical file loaded) Ladder graph compairing physical and genetic marker orders for the chromosome framework.
<mapName>_framework_positions_LG<lgName>_rep<repNum>.pdf (per LG, if graphose) Positions graph of the chromosome framework.
<mapName>_framework_genMap.txt }{ (if writose) Genetic map of the framework map.
<mapName>_genotyping_errors.txt (if writose) Reports detected genotyping errors.
<mapName>_framework_2pt_linkage_separate.pdf (if graphose) pairwise two−point LOD and distance heatmaps of the framework with a single chromosome in each page for easier readability.

<mapName>_framework_2pt_linkage_merged.pdf (if graphose) pairwise two–point LOD and distance heatmaps of the framework map with all chromosomes in each page to view cross–linkage.
<mapName>_framework_marey_maps.pdf (if graphose and physical file loaded) Marey map of the framework map.

4.3.7 Placing all remaining markers on the framework: `placement()`

This function will take all remaining markers one by one and find their most likely position on the framework. The order between two placed markers will not be statistically supported.

The following example places the remaining markers to build the "total" map using the framework and assignments built into the marker information dataframe from the previous step. This completes the map construction for this example.
(CAUTION copying text from a pdf file to a R console may introduce additional characters like spaces)

```
library(SeSAM)
loadExample("testData")
spellSession <- SPELL_cast(segData=mrkInfo_after_loadData, dirOut="SeSAM
    _example_testData/spell_session")

mrkInfo <- placement(
  mrkInfo=mrkInfo_after_buildFramework,
  spellSession=spellSession,
  dirOut="SeSAM_example_testData",
  verbose=TRUE, writose=TRUE, graphose=TRUE
)
```

placement

```
placement(
  mrkInfo, spellSession, typePop=NULL, mrkPool=NULL,
  mappingFunction=SeSAM_par("mappingFunction"),
  minMappingLod=SeSAM_par("minMappingLod"),
  minMultipointLod=SeSAM_par("minMultipointLod"),
  lgFragmentSep=SeSAM_par("lgFragmentSep"),
  phasing=NULL, batchNum=NULL,
  verbose=FALSE, writose=FALSE, graphose=FALSE,
  logFile=NULL, dirOut=getwd()
)
```

Description:

Finds the most likely position of the remaining unmapped assigned markers on the existing framework map. This constitutes a total map which order cannot be statistically guaranteed.

Arguments:

mrkInfo Marker Information dataframe (4.0.1).
spellSession a SpellMapTools object pointing to an active session.
typePop Character scalar. A population type string (2.1.1). Defaults to mrkInfo's typePop

attribute if any.
mrkPool Set of marker names to define the pool of markers to assign.
mappingFunction "haldane" or "kosambi". Mapping function for genetic distances (10.4).
minMappingLod Minimum 2pt linkage to consider adding a marker to a map.
minMultipointLod Minimum multipoint LOD score to consider a marker order significantly better than another.
lgFragmentSep String of characters to denote a smaller fragment of the same reference linkage groups.
phasing TRUE/FALSE, whether or not the seeds have to be drawn from multiple possible phases (CP populations).
batchNum Number of the placement batch used for naming files. Used when running this function multiple times without overriding heatmaps.
verbose TRUE/FALSE. Write information in the standard output and display progress bars for lengthy processes.
writose TRUE/FALSE. Write information in a log file.
logFile Name of the file in which to output the writose information. Defaults to "<dirOut>/SeSAM_<mapName>.log".
graphose TRUE/FALSE. Write Marey graph and heatmap pdf files.
dirOut Base diractory for the output files. Defaults to the working directory.
batchNum Number of the placement batch used for naming files. Used when running this function multiple times without overriding heatmaps.

Value:

Inputted marker information dataframe (4.0.1) with updated genetic chromosome, genetic position and mapping status variables.

File_Output:

<dirOut>/
SeSAM_<mapName>.log (if writose) Default log file.
total_LGs/ (if writose or graphose, for each LG)
<mapName>_total_genMap_LG<lgName>.txt (if writose, per chromosome) Genetic map of the chromosome total map.
<mapName>_total_2pt_linkage_LG<lgName>.pdf (if graphose, per chromosome) pairwise two−point LOD and distance heatmaps of the chromosome total map.
<mapName>_total_marey_map_LG<lgName>.pdf (if graphose and physical file loaded, per chromosome) Marey map of the chromosome total map.
<mapName>_total_ladder_graph_LG<lgName>.pdf (per LG, if graphose and physical file loaded) Ladder graph compairing physical and genetic marker orders for the chromosome total map.
<mapName>_total_positions_LG<lgName>.pdf (per LG, if graphose) Positions graph of the chromosome total map.
<mapName>_total_genMap.txt (if writose) Genetic map of the total map.
<mapName>_total_detailed_info.txt (if writose) Table of information about the placements made to constitute the total map.
<mapName>_total_2pt_linkage_separate.pdf (if graphose) pairwise two−point LOD and distance heatmaps of the total map with a single chromosome in each page for easier readability.
<mapName>_total_2pt_linkage_merged.pdf (if graphose) pairwise two−point LOD and distance heatmaps of the total map with all chromosomes in each page to view cross−linkage.
<mapName>_total_marey_maps.pdf (if graphose and physical file loaded) Marey map of the

total map.
<mapName>_total_ladder_graph.pdf (if graphose and physical file loaded) Ladder graph compairing physical and genetic marker orders for the total map.
<mapName>_total_positions.pdf (if graphose) Positions graph of the total map.

5 Creating *de novo* Linkage Groups

The main genetic map construction algorithm in `SeSAM`, `autoMap`, relies on previously knowing a few markers on each linkage group in order to build one map for each chromosome. In case such information is not available, we present here an alternative method, using some of the same sub-functions used by the autoMap procedure, to build linkage groups from scratch by an iterative process repeating the following sequence:

- A seed marker is randomly drawn from the reduced set of markers not yet assigned to any linkage group.
- A scaffold map is constructed from this seed using the `buildScaffold` (4.3.4) function.
- This new scaffold is compared to previously constructed scaffolds. It may be discarded or merged to others under certain conditions of overlap or cross-linkage. The orders of markers in this set of scaffold maps are not retained, it only serves as a core to group the rest of the markers.
- All other markers in the reduced set are assigned to the scaffolds using the `assignment` (4.3.5) function. Thus the assignment of every marker that isn't part of the scaffolds is evaluated anew at every iteration. This recurring assignment serves to maximize the chances of chosing a seed for the next iteration, that is not on one of the linkage groups already determined.

This process is repeated until the number of remaining unassigned markers is lower than one tenth of the lowest number of markers in any group, considering that these remaining markers probably do not belong to an additional chromosome. The result of this method is a set of marker groups that encompass most markers in the reduced sets. This method is used automatically in `autoMap` if the `usePutativeLGs` is set to `TRUE`.
(CAUTION copying text from a pdf file to a R console may introduce additional characters like spaces)

```
library(SeSAM)
loadExample("testData")
spellSession <- SPELL_cast(segData=mrkInfo_after_loadData, dirOut="SeSAM
   _example_testData/spell_session")

putLGs <- putativeLGs(
  mrkInfo=mrkInfo_after_loadData,
  spellSession=spellSession,
  dirOut="SeSAM_example_testData/Putative_LGs",
  verbose=TRUE, writose=TRUE, graphose=TRUE
)
```

If a physical map is loaded, the putative LGs may be matched against the physical map:

```
phyMap <- extractPhyMap(mrkInfo[ mrkInfo$reduced, ])
putMap <- mapsChrMatch(putLGs, phyMap)
```

putativeLGs

```
putativeLGs(
```

```
  mrkInfo, spellSession, mapName=NULL, typePop=NULL,
  mappingFunction=SeSAM_par("mappingFunction"),
  scaffDistRange=SeSAM_par("scaffDistRange"),
  minDeltaLod=SeSAM_par("minDeltaLod"),
  maxCrossLod=SeSAM_par("maxCrossLod"),
  minMappingLod=SeSAM_par("minMappingLod"),
  minMultipointLod=SeSAM_par("minMultipointLod"),
  locusSpecificityPval=SeSAM_par("locusSpecificityPval"),
  verbose=TRUE, writose=FALSE, graphose=FALSE, logFile=NULL,
  dirOut=getwd(), seedRNG=1
)
```

Description:

Determines putative Linkage Groups by a repeating process of building a scaffold (4.3.4) map from a random yet unassigned marker as seed, filtering the previously built scaffolds by comparison of length, common markers and cross−linkage, and performing an assignment (4.3.5) from the built scaffold until almost all markers are assigned.

Arguments:

mrkInfo Marker Information dataframe (4.0.1).
spellSession a SpellMapTools object pointing to an active session.
mapName Population name for the map. Appears in file names and graph titles. Defaults to mrkInfo's mapName attribute, if any.
typePop Character scalar. A population type string (2.1.1). Defaults to mrkInfo's typePop attribute, if any.
mappingFunction "haldane" or "kosambi". Mapping function for genetic distances (10.4).
scaffDistRange Length 2 numeric vector (min and max). Genetic distance range between two consecutive markers for scaffold construction.
minDeltaLod Minimum 2pt−LOD difference for choosing between two linkage hypotheses (e.g. linkage to two different chromosomes).
maxCrossLod Minimum two point LOD to consider a linkage to an alternative chromosome (cross−linkage) significative.
minMappingLod Minimum 2pt linkage to consider adding a marker to a map.
minMultipointLod Minimum multipoint LOD score to consider a marker order significantly better than another.
locusSpecificityPval p−**value** Threshold to below which the locus specifity test will be rejected.
verbose TRUE/FALSE. Write information in the standard output and display progress bars for lengthy processes.
writose TRUE/FALSE. Write information in a log file.
graphose TRUE/FALSE. Write pdf chart files for the marker and individual metrics, progression of the putative map construction as well as a heatmap for the set of scaffolds.
logFile Name of the file in which to output the writose information. Defaults to "<dirOut>/SeSAM_<mapName>.log".
seedRNG Value to set the seed of R's Random Number Generator. If NULL, lets R set a pseudo −random seed as normal.

Value:

Genetic Map dataframe (2.2) containing only Linkage Group information.

File_Output:

<dirOut>/ (if writose or graphose)
putLGs_<mapName>.log (if writose) Default log file.
<mapName>_putative_LGs.txt (if writose) Putative Map used for drawing seeds.
<mapName>_putative_LGs_scaffold.txt (if writose) Genetic Map of the scaffolds built for the Putatve Map Building process.
<mapName>_putative_LGs_iterations.pdf (if graphose) Plot of the total number of assigned markers over the Putative Map construction iterations.
<mapName>_putative_LGs_assignment_counts.pdf (if graphose) Barplot of assigned marker counts per linkage group in the Putative Map.

The `createPutativeLGs` function is a standalone application of the above and serves to generate these groupings directly from a segregation data file.

createPutativeLGs

```
createPutativeLGs(
  mapName, dirIn=NULL, dirOut=NULL, typePop=NULL,
  segDataFile=NULL, segData_format=NULL, segData_sep="",
  mrkQualFile=NULL, mrkQual_colId=1L, mrkQual_colSco=2L,
  mrkQual_header=FALSE, mrkQual_sep="",
  minQualityScore=SeSAM_par("minQualityScore"),
  maxMissingdata=SeSAM_par("maxMissingdata"),
  maxDistoMappable=SeSAM_par("maxDistoMappable"),
  maxDistoStrict=SeSAM_par("maxDistoStrict"),
  mappingFunction=SeSAM_par("mappingFunction"),
  scaffDistRange=SeSAM_par("scaffDistRange"),
  minDeltaLod=SeSAM_par("minDeltaLod"),
  maxCrossLod=SeSAM_par("maxCrossLod"),
  minMappingLod=SeSAM_par("minMappingLod"),
  minMultipointLod=SeSAM_par("minMultipointLod"),
  locusSpecificityPval=SeSAM_par("locusSpecificityPval"),
  nb_cpu=SeSAM_par("nb_cpu"), mrkPhaseSep=SeSAM_par("mrkPhaseSep"),
  reduceByRedundancy=TRUE, CPDeployPhases=TRUE,
  verbose=TRUE, writose=TRUE, graphose=TRUE, logFile=NULL, seedRNG=1
)
```

Description:

Generates putative Linkage Groups by a repeating process of building a scaffold (4.3.4) map from a random yet unassigned marker as its seed, filtering the previously built scaffolds by comparisson of length, common markers and cross–linkage, and performing an assignment (4.3.5) from the built scaffold until almost all markers are assigned.

Arguments:
mapName Population name for the map. Used to automatically identify files. See "*File" parameters for info about the possible file formats looked for. Appears in file names and graph titles. Looks for files which names contain "segData" for segregation data, "phyMap" for the physical map and "mrkQual" for marker quality data.
dirIn Base directory for input files. All further file paths should be relative to this directory.
dirOut Base directory for the output files. Defaults to either dirIn or the working directory. Will

be created even if writose and graphose are off for the purpose of writing the SpellMapTools bayesian network sessions' cache directories.

typePop A population type code (see (2.1.1). Needs to be given for segregation data file formats which do not contain that information. Overides the population type from the segregation data file if there is one.

indivNames Vector of the names of the individuals of the population. Attached as an attribute to the returned mrkInfo dataframe.

segDataFile Name of the segregation data file. May be a file of one of the .raw, .loc, or .gen formats. The format is detected by the file extention but can be overriden by segData_format.

segData_format Format of the segregation data file (given or detected). Overrides the detected file extention.

segData_sep Separator character for the segregation data file.

genotype_naChar Default character used to represent NA/missing data in genotype files.

mrkQualFile Name of the marker quality file file (2.3.

mrkQual_colId Column number of the marker identifiers in the marker quality file.

mrkQual_colSco Column number of the quality score in the marker quality file.

mrkQual_header TRUE/FALSE whether the marker quality file has column headers.

mrkQual_sep Separator character for the marker quality file.

minQualityScore Quality score threshold for marker quality.

maxMissingdata Tolerated percentage of missing data to consider a marker as "strict".

maxDistoMappable Tolerated percentage of distortion from expected allele frequencies to integrate the marker in the map.

maxDistoStrict Tolerated percentage of distortion from expected allele frequencies to consider a marker to be useable for the mapping steps producing statistically supported map orders (scaffold, framework).

mappingFunction "haldane" or "kosambi". Mapping function for genetic distances (10.4).

scaffDistRange Length 2 numeric vector (min and max). Genetic distance range between two consecutive markers for scaffold construction.

minDeltaLod Minimum 2pt−LOD difference for choosing between two linkage hypotheses (e.g. linkage to two different chromosomes).

maxCrossLod Minimum two point LOD to consider a linkage to an alternative chromosome (cross−linkage) significative.

minMappingLod Minimum 2pt linkage to consider adding a marker to a map.

minMultipointLod Minimum multipoint LOD score to consider a marker order significantly better than another.

locusSpecificityPval p−**value** Threshold to below which the locus specifity test will be rejected.

mrkPhaseSep Character/String used to separate marker name from their phase. Example " mrk1_phase_1". Visible in output files.

reduceByRedundancy TRUE/FALSE. Whether or not to reduce the set of markers for steps 2 to 5 from the set of "strict" markers to a reduced set by filtering redundant markers (no cross −over inbetween). Allows to reduce the computational and memory load for large sets of markers.

CPDeployPhases TRUE/FALSE. Whether or not to deploy the required marker phases for genotyped CP populations. Disable for debugging purposes only.

verbose TRUE/FALSE. Write information in the standard output and display progress bars for lengthy processes.

writose TRUE/FALSE. Write information in a log file.

graphose TRUE/FALSE. Write pdf chart files for the marker and individual metrics, progression of the putative map construction as well as a heatmap for the set of scaffolds.

logFile Name of the file in which to output the writose information. Defaults to "<dirOut>/ SeSAM_<mapName>.log".

seedRNG Value to set the seed of R's Random Number Generator. If NULL, lets R set a pseudo

–random seed as normal.

Value:

Genetic Map dataframe (2.2) containing only Linkage Group information.

File_Output:

<dirOut>/ (if writose or graphose)
spell_session/
SPELL_<mapName>_<YYYY–MM–DD–HH–MM–SS>.raw Prepared segregation data file for session loading.
SPELL_<mapName>_<YYYY–MM–DD–HH–MM–SS>.ped (per session) : Prepared pedigree data file for session loading.
spell–map–tools–<id>/ (per session) : Cache directory for a SpellMapTools bayesian network session.
putLGs_<mapName>.log (if writose) Default log file.
<mapName>_putLGs.txt (if writose) Putative Map used for drawing seeds.
<mapName>_putLGs_scaffold.txt (if writose) Genetic Map of the scaffolds built for the Putatve Map Building process.
<mapName>_putLGs_Assigned_markers_iter.txt (if graphose) Plot of the total number of assigned markers over the Putative Map construction iterations.
<mapName>_putLGs_Scaffold_lengths_iter.txt (if graphose) Plot of the genetic lengths of the scaffolds built for the Putatve Map Building process over the Putative Map construction iterations.
<mapName>_putLGs_Assignment_Barplot.txt (if graphose) Barplot of assigned marker counts per linkage group in the Putative Map.
<mapName>_putLGs_Scaffold_Heatmap.txt (if graphose) LOD and Distance heatmaps of the scaffolds built for the Putatve Map Building process.

6 Filtering outlier individuals with high crossover counts

When a genetic map is computed for a population, the number of crossover events respective to that map can be determined for each individual. This may allow to detect some outlier individuals, for instance produced by pollen contamination.

The `computeCOPerIndiv` function computes the number of crossovers per individual using the framework map of a marker information data frame (see section 4.0.1 page 26). The following example shows how to compute the number of crossovers per individuals following the example for building the framework map (4.3.6). The information is attached as an attribute to the inputted dataframe. To simply obtain the vector of crossover counts, see `getCOPerIndiv` (6).
(CAUTION copying text from a pdf file to a R console may introduce additional characters like spaces)

```
library(SeSAM)
loadExample("testData")

mrkInfo <- computeCOPerIndiv(
  mrkInfo=mrkInfo_after_buildFramework,
  dirOut="SeSAM_example_testData",
  verbose=TRUE, writose=TRUE, graphose=TRUE
)
```

```
nbCO <- getCOPerIndiv(mrkInfo)
```

computeCOPerIndiv

```
computeCOPerIndiv(
  mrkInfo, typePop=NULL, popName=NULL,
  naChar=SeSAM_par("naChar"), nb_cpu=SeSAM_par("nb_cpu"),
  verbose=FALSE, writose=FALSE, graphose=FALSE,
  logFile=NULL, dirOut=getwd()
)
```

Description:

Count the number of crossover events for each individual.

Arguments:

mrkInfo Marker Information dataframe (4.0.1).
typePop Character scalar. A population type string (2.1.1). Defaults to mrkInfo's typePop attribute, if any.
popName Population name for the map. Appears in file names and graph titles. Defaults to mrkInfo's mapName attribute, if any.
naChar Default character used to represent NA/missing data in genotype files.
nb_cpu Number of CPUs useable by the parallelized processes.
verbose TRUE/FALSE. Write information in the standard output and display progress bars for lengthy processes.
writose TRUE/FALSE. Write information in a log file.
graphose TRUE/FALSE. Write pdf chart files for the marker and individual metrics, progression of the putative map construction as well as a heatmap for the set of scaffolds.
logFile Name of the file in which to output the writose information. Defaults to "<dirOut>/SeSAM_<mapName>.log".
dirOut Base directory for the output files. Defaults to either dirIn or the working directory. Will be created even if writose and graphose are off for the purpose of writing the SpellMapTools bayesian network sessions' cache directories.

Value:

Inputted marker information dataframe (4.0.1) with a "nbCO" attribute which is a vector of the number of cross−overs per individual.

File_Output:

<dirOut>/ (if writose or graphose)
SeSAM_<popName>.log (if writose) Default log file.
<popName>_histogram_CO_indiv.pdf (if graphose) Histogram of the number of crossovers per individual.

getCOPerIndiv

```
getCOPerIndiv(
```

```
  mrkInfo, typePop=NULL,
  naChar=SeSAM_par("naChar"), nb_cpu=SeSAM_par("nb_cpu"),
)
```

Description:

Get the vector of cross–over counts per individual computed by computeCOPerIndiv, either by returning an existing 'nbCO' attribute or computing it.

Arguments:

mrkInfo Marker Information dataframe (4.0.1).
typePop Character scalar. A population type string (2.1.1). Defaults to mrkInfo's typePop attribute, if any.
naChar Default character used to represent NA/missing data in genotype files.
nb_cpu Number of CPUs useable by the parallelized processes.

Value:

Vector of the number of cross–overs per individual.

The `filterIndivByNbCO` function uses the above function to filter the individuals which crossover count are over a threshold value.

filterIndivByNbCO

```
filterIndivByNbCO(
  mrkInfo, maxNbCO=NULL, typePop=NULL,
  popName=NULL, verbose=FALSE, writose=FALSE, graphose=FALSE,
  logFile=NULL, dirOut=getwd(),
  segData_format=c("raw", "loc", "gen"),
  naChar=SeSAM_par("naChar"), nb_cpu=SeSAM_par("nb_cpu")
)
```

Description:

Count the number of crossover events for each individual.

Arguments:

mrkInfo Marker Information dataframe (4.0.1).
maxNbCO Threshold of number of crossovers above which individuals are filtered out. Defaults to the 0.01 quantile of a Poisson law centered on the mean number of crossovers per individual .
popName Population name for the map. Appears in file names and graph titles. Defaults to mrkInfo's mapName attribute, if any.
typePop Character scalar. A population type string (2.1.1). Defaults to mrkInfo's typePop attribute, if any.
verbose TRUE/FALSE. Write information in the standard output and display progress bars for lengthy processes.
writose TRUE/FALSE. Write information in a log file.

graphose TRUE/FALSE. Write pdf chart files for the marker and individual metrics, progression of the putative map construction as well as a heatmap for the set of scaffolds.
logFile Name of the file in which to output the writose information. Defaults to "<dirOut>/SeSAM_<mapName>.log".
dirOut Base directory for the output files. Defaults to either dirIn or the working directory. Will be created even if writose and graphose are off for the purpose of writing the SpellMapTools bayesian network sessions' cache directories.
segData_format Format of the outputted segregation data file.
naChar Default character used to represent NA/missing data in genotype files.
nb_cpu Number of CPUs useable by the parallelized processes.

Value:

Filtered marker information dataframe (see section 4.0.1 page 26).

File_Output:

<dirOut>/ (if writose or graphose)
SeSAM_<popName>.log (if writose) Default log file.
<popName>_histogram_CO_indiv.pdf (if graphose) Histogram of the number of crossovers per individual.

The `filterSegDataFileByCO` does the same as the above function to filter the individuals in a segregation data file by number of crossovers. To do so, the function uses a map file. The markers of the inputted map file are all considered in the computations as if they were part of a scaffold map.

filterSegDataFileByCO

```
filterSegDataFileByCO(
  popName, maxNbCO=NULL, dirIn=NULL, typePop=NULL,
  segDataFile=NULL, segData_format_in=NULL, segData_sep="",
  nb_cpu=SeSAM_par("nb_cpu"),
  genMapFile=NULL, genMap_colId=1L, genMap_colChr=2L,
  genMap_colPos=3L, genMap_header=FALSE, genMap_sep="",
  genMap_naChars='-',
  verbose=FALSE, writose=FALSE, graphose=FALSE,
  logFile=NULL, dirOut=getwd(),
  segData_format_out=c("raw", "loc", "gen")
)
```

Description:

Count the number of crossover events for each individual.

Arguments:

popName Population name for the map. Appears in file names and graph titles. Defaults to mrkInfo's mapName attribute, if any.
maxNbCO Threshold of number of crossovers above which individuals are filtered out. Defaults to the 0.01 quantile of a Poisson law centered on the mean number of crossovers per individual

.

dirIn Base directory for input files. Files are automatically looked for in this directory if the specific **arguments** aren't specified.

typePop Character scalar. A population type string (2.1.1). Defaults to mrkInfo's typePop attribute, if any.

segDataFile Name of the segregation data file. May be a file of one of the .raw, .loc, or .gen formats. The format is detected by the file extention but can be overriden by segData_format.

segData_... (segData_format, segData_sep, segData_naChar, segData_indivNames) Additional parameters passed down to the segregation data file reading function (see 2.1.7).

nb_cpu Number of CPUs useable by the parallelized processes.

genMapFile Name of the genetic map file. See 2.2.

genMap_... (genMap_colId, genMap_colChr, genMap_colPos, genMap_header, genMap_sep, genMap_naChars): Additional parameters passed down to the genetic map file reading function (2.2).

verbose TRUE/FALSE. Write information in the standard output and display progress bars for lengthy processes.

writose TRUE/FALSE. Write information in a log file.

graphose TRUE/FALSE. Write pdf chart files for the marker and individual metrics, progression of the putative map construction as well as a heatmap for the set of scaffolds.

logFile Name of the file in which to output the writose information. Defaults to "<dirOut>/ SeSAM_<mapName>.log".

dirOut Base directory for the output files. Defaults to either dirIn or the working directory. Will be created even if writose and graphose are off for the purpose of writing the SpellMapTools bayesian network sessions' cache directories.

segData_format_out Format of the outputted segregation data file.

Value:

Filtered marker information dataframe (see section 4.0.1 page 26).

File_Output:

<dirOut>/ (if writose or graphose)
SeSAM_<popName>.log (if writose) Default log file.
<mapName>_histogram_distortion.pdf : (if graphose) Histogram of the markers' distortion.
<mapName>_histogram_md_markers.pdf : (if graphose) Histogram of the markers' missing data percentage.
<mapName>_histogram_indiv_markers.pdf : (if graphose) Histogram of the individuals' missing data percentage.
<mapName>_mapping_categories_count (if graphose) Concentric circles representing the proportions of each categories of markers.
<popName>_histogram_CO_indiv.pdf (if graphose) Histogram of the number of crossovers per individual.

7 Simulating segregation data sets

SeSAM features a module to simulate genetic maps and associated segregation data. This is extremely useful to assess the quality of a process of map construction, by comparing *a posteriori* the map constructed with the initial map used to simulate the segregation data. Simulating data may also help to compare expected and observed levels of heterozygosity in some types of populations. It may also help to figure out to what extent a given level of genotyping errors or missing data is expected to be able to alter the outcome

of a mapping process (although in that case, the way missing data are distributed in the experimental data can also play a significant role).

7.1 Simulating a genetic map

The `generateGenMap` function generates a genetic map by spreading a number or markers across a number of chromosomes either regularly or randomly (with a uniform distribution). The following example generates a map with two chromosomes with 100 and 200 markers respectively, spread uniformly on genetic lengths of 50 and 150 cM respectively.
(CAUTION copying text from a pdf file to a R console may introduce additional characters like spaces)

```
library(SeSAM)
dirOut <- "./simulation"
genMap <-  generateGenMap(
  nbChr=2L,
  nbMrk=c(100, 200),
  mapLength=c(50, 150),
  verbose=TRUE,
  writose=TRUE,
  dirOut=dirOut
)
```

generateGenMap

```
generateGenMap <- function(
  nbChr, nbMrk, mapLength, mrkDist=c("uniform", "regular"),
  popName="sim", verbose=FALSE, writose=FALSE,
  logFile=NULL, dirOut=getwd(), seedRNG=NULL
)
```

Description:

Generate a genetic map from given parameters.

Arguments:

nbChr Positive integer **value** of the number of chromosomes to generate.
nbMrk Positive integer vector. Number of markers for each chromosome. Values will be ignored/recycled if the length of this argument doesn't match the number of chromosomes ('nbChr').
mapLength Positive integer vector. Genetic length of each chromosome in centiMorgan (cM). Values will be ignored/recycled if the length of this argument doesn't match the number of chromosomes ('nbChr').
mrkDist Method for placing markers along the length of a chromosome:
"uniform" : Marker positions will be drawn in a uniform distribution except for the makers at the boundaries of the chromosome.
"regular" : Markers will be evenly spaced along the length of the chromosome.
popName Character string. Name of the population (for file naming).
verbose TRUE/FALSE various informative prints to the standard output. Loading bars for the segregation simulation process.
writose TRUE/FALSE various informative prints to a log file. File(s) printed for the generated genetic map and/or segregation data.
logFile Filename of the log file for the writose behaviours.
dirOut Path of the directory in which to write files for the writose behaviours.
seedRNG **Value** to set the seed of R's Random Number Generator. If NULL, lets R set a pseudo

–random seed as normal.

Value:

Genetic Map dataframe (2.2) of the generated map.

File_Output:

<dirOut>/ (if writose)
SeSAM_<popName>.log: default log file.
[sim]_<popName>_genMap.txt : Generated map file.

7.2 Simulating segregation data

The `simulatePopFromMap` function simulates the segregation of marker alleles for a given map. To describe the genetic structure of the population to simulate, the simulations use a system of population pedigree (10.1) which allows to simulate a wide variety of population types:

Population	Type	Supported by `SeSAM`
Successive Selfings (RIL self)	Fx	x >1
Intermating + Successive Selfings (IRIL self)	IxFy	any x, y>5
Successive Backcrosses	BCx	x >0
Successive Backcrosses + Successive Selfings	BCxSy	x >0, y >0
Cross-Polinated	CP	CP

Table 5: Population types possible to simulate with `SeSAM`

The following example simulates the segregation of the markers from the previous example's genetic map in a back-cross population.
(CAUTION copying text from a pdf file to a R console may introduce additional characters like spaces)

```
library(SeSAM)
dirOut <- "./simulation"
segData <- simulatePopFromMap(
  genMap=genMap,
  nbIndiv=200,
  typePop="BC1",
  popName="sim_BC1",
  verbose=TRUE,
  writose=TRUE,
  dirOut=dirOut
)
```

simulatePopFromMap

```
simulatePopFromMap(
  genMap, nbIndiv, typePop, popName="sim", segTypeProb=NULL,
  segType=NULL, phase=NULL, interference=1, missingDataPercent=0,
  shuffleMrk=TRUE, scramblePhases=TRUE,
  naChar=SeSAM_par("naChar"), nb_cpu=SeSAM_par("nb_cpu"),
  verbose=FALSE, writose=FALSE, logFile=NULL,
```

```
  dirOut=getwd(), segData_format=c("raw", "loc", "gen"),
  seedRNG=NULL
)
```

Description:

Generate segregation data for a given genetic map, number of individuals and population type.

Arguments:

paramName Param **description**.

genMap A genetic map dataframe (2.2).
nbIndiv Positive integer **value**. Number of individuals of in the simulated population.
typePop Population type code string (2.1.1).
segTypeProb Length 5 vector of values between 0 and 1 with a sum of 1. May be named with the first 5 values of `segTypes()`(2.1.1). Probabilities for each segregation type to be drawn for a 'CP' population. Ignored if the 'segType' parameter is set or if typePop is not 'CP'. If NULL and 'segType' is NULL, segregation types are drawn with equal probabilities.
segType Vector of the segregation types of the markers. Length must be at least equal to the number of rows in genMap. Ignored if 'typePop' is not 'CP'. If NULL segregation types are drawn according to 'segTypeProb'.
phase Vector of the marker phases (10.5. Length must be at least equal to the number of rows in genMap. Ignored if 'typePop' is not 'CP'. Phases 3 and 4 are converted to phases 1 and 2 respectively for markers which segregation types follow a backcross−like segregation.
interference Positive numeric **value**. Cross−Over interference **value**.
missingDataPercent Percentage of missing data to introduce at the end of the simulation.
genotypingErrorPercent Percentage of genotyping errors to introduce at the end of the simulation.
missingDataGammaShape Length 2 numeric vector. Shape parameters of gamma functions that define the probability distribution of missing data being added across markers and individuals (respectively). If unspecified or invalid, defaults to a uniform probability distribution.
genotypingErrorGammaShape Length 2 numeric vector. Shape parameters of gamma functions that define the probability distribution of genotyping errors being added across markers and individuals (respectively). If unspecified or invalid, defaults to a uniform probability distribution.
shuffleMrk Shuffle the order of markers in the output.
scramblePhases Switch the order of the parental alleles randomly to obfuscate the map's phases. Ignored if 'typePop' is not 'CP'.
segData_format Format used for the segregation data file (2.1).
naChar Single character. Character used to represent missing genetic data in written file(s).

Value:

Segregation Data dataframe for the simulated population (2.1).

File_Output:

<dirOut>/ (if writose) \itemize{
SeSAM_<popName>.log: default log file.
[sim]_<popName>_segData_original.<segData_format>: Segregation data file as simulated

before marker shuffling and phase scrambling.

[sim]_<popName>_segData.<segData_format>: Segregation data file corresponding to the returned segregation data dataframe.

7.3 Complete Simulation

The `simulatePop` function combines `generateGenMap` and `simulatePopFromMap`, generating a genetic map from given parameters and simulating the segregation for its markers in a given population type. (CAUTION copying text from a pdf file to a R console may introduce additional characters like spaces)

```
dirOut <- "./simulation"
sim <-  simulatePop(
  nbChr=2L,
  nbMrk=c(100, 200),
  mapLength=c(200, 300),
  nbIndiv=200,
  typePop="BC1",
  popName="sim_BC1",
  verbose=TRUE,
  writose=TRUE,
  dirOut=dirOut
)
genMap <- sim$genMap
segData <- sim$segData
phyMap <- sim$phyMap
rm(sim)
```

simulatePop

```
simulatePop(
  nbChr, nbMrk, mapLength, nbIndiv, typePop,
  mrkDist=c("uniform", "regular"), popName="sim", segTypeProb=NULL,
  segType=NULL, phase=NULL, interference=1, missingDataPercent=0,
  shuffleMrk=TRUE, scramblePhases=TRUE, fakePhyMap=TRUE,
  naChar=SeSAM_par("naChar"), nb_cpu=SeSAM_par("nb_cpu")
  verbose=FALSE, writose=FALSE, logFile=NULL, dirOut=getwd(),
  segData_format=c("raw", "loc", "gen"), seedRNG=NULL
)
```

Description:

Generate a genetic map from a set of parameters and its segregation data for a given number of individuals and population type.

Arguments:

nbChr Positive integer **value** of the number of chromosomes to generate.
nbMrk Positive integer vector. Number of markers for each chromosome. Values will be ignored/recycled if the length of this argument doesn't match the number of chromosomes ('nbChr').
mapLength Positive integer vector. Genetic length of each chromosome in centiMorgan (cM). Values will be ignored/recycled if the length of this argument doesn't match the number of

chromosomes ('nbChr').
mrkDist Method for placing markers along the length of a chromosome:
"uniform" : Marker positions will be drawn in a uniform distribution except for the makers at the boundaries of the chromosome.
"regular" : Markers will be evenly spaced along the length of the chromosome.
popName Character string. Name of the population (for file naming).
nbIndiv Positive integer **value**. Number of individuals of in the simulated population.
typePop Population type code string (2.1.1).
segTypeProb Length 5 vector of values between 0 and 1 with a sum of 1. May be named with the first 5 values of `segTypes()`(2.1.1). Probabilities for each segregation type to be drawn for a 'CP' population. Ignored if the 'segType' parameter is set or if typePop is not 'CP'. If NULL and 'segType' is NULL, segregation types are drawn with equal probabilities.
segType Vector of the segregation types of the markers. Length must be at least equal to the number of rows in genMap. Ignored if 'typePop' is not 'CP'. If NULL segregation types are drawn according to 'segTypeProb'.
phase Vector of the marker phases (10.5. Length must be at least equal to the number of rows in genMap. Ignored if 'typePop' is not 'CP'. Phases 3 and 4 are converted to phases 1 and 2 respectively for markers which segregation types follow a backcross−like segregation.
missingDataPercent Percentage of missing data to introduce at the end of the simulation.
interference Positive numeric **value**. Cross−Over interference **value**.
shuffleMrk Shuffle the order of markers in the output.
scramblePhases Switch the order of the parental alleles randomly to obfuscate the map's phases. Ignored if 'typePop' is not 'CP'.
segData_format Format used for the segregation data file (2.1).
naChar Single character. Character used to represent missing genetic data in written file(s).

Value:

list of two named elements:
'genMap' : Genetic Map dataframe (see \code{\link{isGenMap}}) of the generated map.
'segdata' : Segregation Data dataframe for the simulated population. \code{\link{SegData}}

File_Output:
<dirOut>/ (if writose)
SeSAM_<popName>.log: default log file.
[sim]_<popName>_genMap.txt: Generated genetic map file.
[sim]_<popName>_segData_original.<segData_format>: Segregation data file as simulated before marker shuffling and phase scrambling.
[sim]_<popName>_segData.<segData_format>: Segregation data file corresponding to the returned segregation data dataframe.
[sim]_<popName>_fake_phyMap.txt: (if fakePhyMap) Fake physical map obtained from multiplying the generated genetic positions by 1000 and rounding.

8 Consensus map construction

The `consensusMap` is an independent module for building consensus from maps of the same species. The consensus map is defined as the map for which, the distances between markers are minimally different from the distances in the input maps.This is obtained through a minimization algorithm over the difference between the consensus map distances and the input map distances. This algorithm is analogous to balancing a system of "pegs" and "springs" where each marker is a "peg" attached in a network of "springs", which are observed distances, to neighboring markers. In this analogy, the criterion score to minimize could be

seen as the overall "tension" (in fact energy) of the system.

- For each marker of each map, the distances between it and a number of neighboring markers are measured. Each of these observed distances will factor in the criterion score to optimize. In order to reduce the number of variables for the optimization, only distances over `cons_minDist`, preferentially below `cons_prefDist` and up to a number of `cons_maxNei` neighbors are retained through a recursive search. The distances may be associated with a weight value. This weight or an observed distance is inversely proportional to the number of inputted maps which feature the markers that bound this distance. This is to ensure that some markers do not dominate the others in virtue of being featured in more maps.
- An initial consensus map is defined with all the markers of all the inputted maps with their average position between all maps.
- A criterion score is defined as the weighed sum of differences between observed distances and the corresponding distances in the consensus map.
- The positions of markers in the consensus map are adjusted by iteration in order to minimize this criterion until a `cons_maxIter` number of iterations have been computed or until the difference in score between two iterations falls under `cons_scoreSig`.

```
library(SeSAM)
consensusMap(
  SeSAM_consensusTest_map1(),
  SeSAM_consensusTest_map2(),
  SeSAM_consensusTest_map3()
)
```

funName

```
consensusMap(
  ..., dirIn=NULL, colId=1L, colChr=2L, colPos=3L, mapNames=NULL,
  header=FALSE, sep="\t", cons_minDist=SeSAM_par("cons_minDist"),
  cons_prefDist=SeSAM_par("cons_prefDist"),
  cons_maxNei=SeSAM_par("cons_maxNei"),
  doNorm=TRUE, mapsWeight=NULL, weightByCount=FALSE,
  cons_scoreSig=SeSAM_par("cons_scoreSig"),
  cons_maxIter=SeSAM_par("cons_maxIter"),
  basename=NULL, verbose=FALSE, writose=FALSE,
  graphose=FALSE, logFile=NULL, dirOut=NULL
)
```

Description:

Builds a consensus map from several maps by a process of minimizing the difference of marker—to—marker distances between the consensus map and each of the the original maps.

Arguments:

... Any number of file names for the map files to read. May accept vectors of file names. If left empty, all the files of the dirIn argument will be read.
dirIn Directory containing the map files. If any unnamed (...) filename **arguments** precede, these will be looked for relative to dirIn. If left NULL, those file names are searched as absolute paths.
colId Number of the column to read/write the marker's names when reading maps from files.

colChr Number of the column to read/write the marker's chromosome/linkage group when reading maps from files.
colPos Number of the column to read/write the marker's genetic position when reading maps from files.
mapNames Names for the maps in the order of files given. If NULL, defaults names are put in place.
header Whether the map files to read have a header line.
sep Character used to separate the table's cells when reading maps from files. Defaults to any number of white space characters.
cons_prefDist Preferred distance from marker for the consensus neighbour search.
cons_minDist Minimum distance from marker for the consensus neighbour search.
cons_maxNei Maximum neighbors per marker for the consensus neighbour search.
doNorm Work with normalized map positions (highly recommended).
mapsWeight Weight factor to apply to distances from each map, respectively.
weightByCount Whether or not to additively factor each distance's weights inversely to its marker's number of copies across the set of maps.
cons_scoreSig Significative score change for consensus map optimization.
cons_maxIter Maximum number of iterations for consensus map optimization.
basename Name used as a base for the outputed folders and files.
verbose If true, will print progress on steps. 0/FALSE : no printing, 1/TRUE : General status, number of conflicting markers removed, consensus map iterations, >1 : Consensus map progress bar per iteration.
writose Level of verbosity for the log file output.
graphose Whether or not to generate graphs of the maps (starting and consensus).
logFile Name of the file in which to output the writose information. Defaults to "<basename>.log ".
dirOut Path to the directory where the consensus map and other files should be outputed. **Value** of NULL will not write a file.

Value:

Optimized consensus map dataframe. Each LG submap has its own score as attribute and the object has the total score.

File_Output:
<dirOut>/ (if writose or graphose)
SeSAM_<basename>.log (If writose): Default Log File.
<basename>_positions_graphs/ (if graphose) : Graphs comparing positions of markers between all inputted maps and the consensus map.
<basename>_positions_graph_<chromosome>.pdf (for each chromosome)
<basename>_ladder_graphs/ : Graphs comparing the order of markers between two maps at a time. Only markers in common between both maps may be represented.
<basename>_ladder_graph_<chromosome>/ (for each chromosome)
<basename>_ladder_graph_<chromosome>_<mapA>_<mapB>.pdf (for each pair of maps A and B including all inputted maps and the consensus map)
<basename>_merged_ladder_graphs/ : Graphs comparing the order of markers between all maps. Only markers in common between all maps may be represented.
<basename>_merged_ladder_graphs_<chromosome>.pdf (for each chromosome)

9 List of parameters and default values

The default values for the main parameters used by the various modules in `SeSAM` are stored and accessed through the `SeSAM_par` function. These default values are appropriate for most data sets. The stored parameter values may also be modified for the running R session and all functions which take values from `SeSAM_par` for their defaults will use the modified value. `SeSAM_par` is used similarly to the `options` function as the following examples show:

```
SeSAM_par() # Named list of all parameters
SeSAM_par("maxMissingdata") # Returns value itself.
SeSAM_par(maxMissingdata=60) # Set parameter. Full list returned invisibly
    .
SeSAM_par("maxMissingdata") # New value remains.
SeSAM_par("maxMissingdata", "minDeltaLod") # Named list of both parameter
    values.
SeSAM_par("maxMissingdata", minDeltaLod=4) # Named list of both parameter
    values.
```

Aditionally, this function will verify whether inputted values are valid and throw an error if not. The parameters managed through this function with their default values (in brackets) are the following:
Mapping values:

- **maxMissingdata [50]:** Tolerated percentage of missing data to consider a marker as "strict".
- **maxDistoMappable [90]:** Tolerated percentage of distortion from expected allele frequencies to integrate the marker in the map.
- **maxDistoStrict [10]:** Tolerated percentage of distortion from expected allele frequencies to consider a marker "strict".
- **minQualityScore [0.8]:** Quality score threshold for marker to consider a marker as "strict".
- **minDeltaLod [3]:** Minimum 2pt-LOD difference for choosing between two linkage hypotheses (e.g. linkage to two different chromosomes).
- **maxCrossLod [2]:** Minimum two point LOD to consider a linkage to an alternative chromosome (cross-linkage) significant.
- **locusSpecificityPval [10e-4]:** Specificity test, based on two-locus segregation expectations (only with backcross populations). p-value threshold.
- **minMultipointLod [5]:** Minimum multipoint LOD score to consider a marker order significantly better than another.
- **minMappingLod [3]:** Minimum 2pt linkage to consider adding a marker to a map.
- **scaffDistRange [c(min=5, max=50)]:** Length 2 numeric vector (min and max). Genetic distance range between two consecutive markers for scaffold construction.
- **maxFrameworkExpansion [0.01]:** Map length expansion rate allowed per integrated marker in the framework map.

Specs:

- **mrkPhaseSep ["_phase_"]:** Character/String used to separate marker name from their phase.
- **lgFragmentSep ["_sub_"]:** Character/String used to denote a smaller fragment of the same reference linkage groups.

- **mappingFunction ["haldane"]:** "haldane" or "kosambi". Mapping function for genetic distances (10.4).
- **nb_cpu [10]:** Number of CPUs usable for parallelization.
- **nb_rep [10]:** Number of replicates for scaffold construction.
- **naChar ["-"]:** Default character used to represent NA/missing data in files.

Default Populations:

- **ri_self ["F6"]:** Default RIL population obtained by selfing.
- **ri_sib ["F6"]:** Default RIL population obtained by sib-mating.
- **iri_self ["I4F6"]:** Default IRIL population obtained by selfing.
- **iri_sib ["I4F6"]:** Default IRIL population obtained by sib-mating.

Consensus Parameters:

- **cons_prefDist [10]:** Preferred distance from marker for the consensus neighbor search.
- **cons_minDist [2]:** Minimum distance from marker for the consensus neighbor search.
- **cons_maxNei [Inf]:** Maximum number of neighbors per marker for the consensus neighbor search.
- **cons_scoreSig [0.05]:** Significant score change for consensus map optimization.
- **cons_maxIter [100L]:** Maximum number of iterations for consensus map optimization.

funName

```
SeSAM_par(...)
```

Description:

SeSAM global parameters handler.

Arguments:

paramName Param **description**.
... Parameters by name to set a **value**. Parameter name strings to get values.

Value:

Full list (invisibly) if only setting. List (unlisted if only 1) of all values affected/requested. Full list if given with no **arguments**.

10 Further algorithmic details

10.1 Population pedigrees

`SeSAM` describes population types internally with a system of pedigrees. In the `autoMap` mapping (4.1) pipeline, such pedigrees are used in the `SpellMaptools` C++ module used to compute recombination rates. In simulations (7), pedigrees are used to describe the sequence of crossings between individuals to simulate data from any type of segregating population that can be used in `SeSAM`.

Example .ped file (BC1)

```
gen;id;p1;p2
A;1;0;0
B;2;0;0
F1;3;1;2
BC1;4;1;3
BC1;5;1;3
BC1;6;1;3
BC1;7;1;3
BC1;8;1;3
BC1;9;1;3
BC1;10;1;3
```

10.2 Marker segregation distortion

`SeSAM` measures distortion using a score which indicates the relative difference between observed and expected genotype frequencies. The purpose of such an index is to be able to calculate distortion the same way regardless of the type of population. So it can be calculated with any set G of all possible single-locus genotypes in the population.

$$Distortion\ Index = \frac{1}{2} * \frac{\sum_{i=1}^{n(G)} \left| g_i^{obs} - g_i^{exp} \right|}{\min\left(g^{exp}\right)} \tag{1}$$

With g^{obs} the observed frequencies of genotypes G and g^{exp} the expected frequencies of genotypes G for the marker.

10.3 Estimation of recombination fractions per meiosis

Backcross, F2, and F3 populations

Recombination rates (r) are estimated by Maximum Likelihood (ML) using the C++ SpellMapTools module of the SPELL-QTL software developed by Jasson & Leroux (2017). That software is built on a framework in which crossovers arise independently (no crossover interference), thereby allowing a Markov process-based computation of the likelihood to produce any genotype in the considered population. Essentially, given the genotype and pedigree of an individual in the population considered, the Markovian nature of the recombination events along the genome makes it feasible to sum over all meiotic histories compatible with that given genotype. Of course these likelihoods depend on the recombination rates between each pair of adjacent markers, while the likelihood of a putative map is obtained by taking the product of the likelihoods of each individual in the population. Furthermore, this software implements an EM algorithm to handle missing data for BC (and thus RIL and IRIL) and F2 populations. For F3 populations, the EM is first carried out by SpellMapTools as for an F2 populations, leading to (biased) recombination fractions, but then the corrected recombination rates are inferred with a likelihood maximization.

Recombinant Inbred Line populations (RIL: F5+) (5 or more generations of selfing or sib-mating after the F2 without previous generations of intermating). `SeSAM` first calculates the proportion of recombinant individuals R (which would be equal to the recombination rate r in a back-cross population), and then

this value is corrected for multiple meioses to obtain r using the formula given by Haldane & Waddington (1931):
For selfing inbreeding generations:

$$r = \frac{R}{2(1-R)} \tag{2}$$

For sib-mating inbreeding generations:

$$r = \frac{R}{2(2-3R)} \tag{3}$$

Where r is the recombination rate for RILs and R the recombination rate estimated by treating the population as a back-cross population.

Intermated Recombinant Inbred Line populations (IRIL: IxF5+) with I intermating generations between F2 individuals carried before recurrent inbreeding. SeSAM proceeds as for RILs except that the formulae of Haldane and Waddington are replaced by those of Winkler et al. (2003) which take into account recombination occurring during the intermating generations as follows:
For selfing inbreeding generations:

$$R = \frac{1}{2}(1 - \frac{1-2r}{1+2r}(1-r)^I) \tag{4}$$

For sib-mating inbreeding generations:

$$R = \frac{1}{2}(1 - \frac{1-2r}{1+6r}(1-r)^I) \tag{5}$$

Where r is the recombination rate *per meiosis*, R the proportion of recombinant individuals (recombination rate estimated by treating the population as a back-cross population), and I the number of random intermating generations between the F2 and the inbreeding process.

10.4 Computation of genetic distances from recombination fractions: distance functions

Recombination fractions per meiosis may not be considered as valid genetic distances since they are not additive (because they do not account for multiple COs). On the other hand, true genetic distances in (centi)Morgan represent the expected number of COs, which is an additive metrics. To compute centiMorgan distances from recombination fractions, SeSAM proposes the two mostly used distance functions: the Haldane mapping function (Haldane, 1919) which assumes no CO interference, or the Kosambi mapping function (Kosambi, 1944) which uses a simple linear modeling of CO interference. Either function may be more appropriate depending on the species considered, one possible choice criterion being the level of additivity achieved by the distances (e.g. the quality of the correlation between d_{AC} and $d_{AB} + d_{BC}$ across all ordered triplets of linked markers A, B, C). The Haldane distance function is:

$$d\ (cM) = -50 \ln(1-2r) \tag{6}$$

The Kosambi distance Function is:

$$d\ (cM) = 25 \ln(\frac{1+2r}{1-2r}) \tag{7}$$

10.5 Specifics of cross-pollinated populations

Because cross-pollinated (CP) populations are the result of crossing two partly heterozygous parents, the types of segregation vary across markers, depending on the parental configuration of alleles.

Segregation Types

Unlike populations obtained through a variable number of crossings from a pair of homozygous parents, in CP populations, there are a greater number of possible segregation types. Depending on the locus, the parents may involve just two alleles, in which case we have the same segregation types as those observed in BC or F2 populations derived from homozygous parents. However when the parents involve more than 2 alleles, truly novel types arise. We use joinMap's notation (Stam, 1993) to classify all these different cases according to the alleles present in each parent without any information on the phasing:

Class	Segregation Type	Example
pseudo-F2	<abxcd>	AT x GC
	<efxeg>	AT x AC
	<hkxhk>	AT x AT
pseudo-BC♀	<lmxll>	AT x AA
pseudo-BC♂	<nnxnp>	AA x AT
monomorph	mono	AA x AA

This has the consequence that the amount of linkage information between a pair of loci depends on the segregation types at those loci. While the linkage in both parents separately (male and female) can be determined in the case of two loci each having 4 alleles, more often having two pseudo-F2 loci only provides the sex averaged recombination rate. If one of the loci is a pseudo-BC locus, then one obtains linkage information for either male or female meiosis, unless one has one pseudo-BC♀ locus and one pseudo-BC♂ locus in which case there is no linkage information at all. To deal with this issue, we have developed a strategy for the scaffold map construction (4.3.4) which prioritizes between segregation types with the aim of obtaining a map with as regularly spaced pseudo-F2 markers as possible. The objective of this method is to always have a pseudo-F2 marker at a reasonable distance in any interval, inserting pseudo-BC markers in the map at later stages. Rather than simply selecting the most strongly linked marker within range of the extremity to extend, the candidate marker selection for CP scaffolds proceeds as follows:

- Filter potential candidates by LOD and distance as for non-CP populations.
- If there are not at least two markers in the scaffold with either pseudo-F2 or pseudo-BC♂ segregation type, exclude pseudo-BC♂ candidate markers.
- If there are not at least two markers in the scaffold with either pseudo-F2 or pseudo-BC♀ segregation type, exclude pseudo-BC♀ candidate markers.
- Find the candidate closest (in genetic distance) to the external marker of the side being extended.
- If this candidate is pseudo-F2, proceed to tests with this candidate.
- Else, find the pseudo-F2 candidate closest to the external marker of the side being extended.
- If the distance between the closest overall candidate and the closest pseudo-F2 candidate is lower than the minimum distance interval for the scaffold map, proceed to tests with the pseudo-F2 candidate.
- Else proceed to tests with the closest overall candidate.

This priority to pseudo-F2 markers for scaffold construction operates conjointly to the priority given to twin markers (10.6)

Phases

The allelic phase of a set of markers (corresponding to the parental haplotypes) is generally not known with CP populations. Thus, parental genotypes (e.g. for a given pair of markers) must be interpreted according to multiple possible phases (*i.e.* haplotypic configurations) to analyze linkage. When looking at loci individually, there are different possible parental configurations. For instance a pseudo-F2 marker has four possible parental configurations while pseudo-BC markers have two. Specifically, a <hkxhk> marker with parental genotypes AT x AT has the following possible parental phases: AT x AT, TA x AT, AT x TA

and `TA x TA` while a `<nnxnp>` marker with parental genotypes `AA x AT` has the following possible parental phases: `AA x AT` and `AA x TA`. Because of this 'one to many' mapping, one must account for all possible parental phases when analyzing the linkage between two loci.

When treating CP markers, we allow the maximum likelihood procedure to search for recombination rates (r_{ML}, see below) between 0 and 1 rather than between 0 and 0.5. Indeed, r_{ML} values over 0.5 can be interpreted as one minus the actual recombination rate.

$$r = \begin{cases} \text{if } r_{ML} > 0.5 & 1 - r_{ML} \\ \text{else} & r_{ML} \end{cases} \tag{8}$$

where r_{ML} is the recombination rate estimated by maximum likelihood between 0 and 1. As a consequence of extending as such the ML procedure to a range of 0 to 1, the LOD will be the same whether we measure the actual recombination rate or one minus that value. There are then two possible situations when a significant LOD is measured between two markers: the markers are either "linked" ("direct" parental configuration, $r_{ML} < 0.5$) or "anti-linked" ("opposite" parental configuration, $r_{ML} > 0.5$). A pair of markers being anti-linked indicates that they would be linked if the parental configuration of one of them had been assigned the opposite configuration (where both parents are inverted, for example AT x AT to TA x TA). This method therefore allows one to perform a single ML test covering two parental phase assumptions; if linkage is found, one can then reconstitute a valid two-locus haplotype by looking *a posteriori* at the estimated value of r_{ML}.

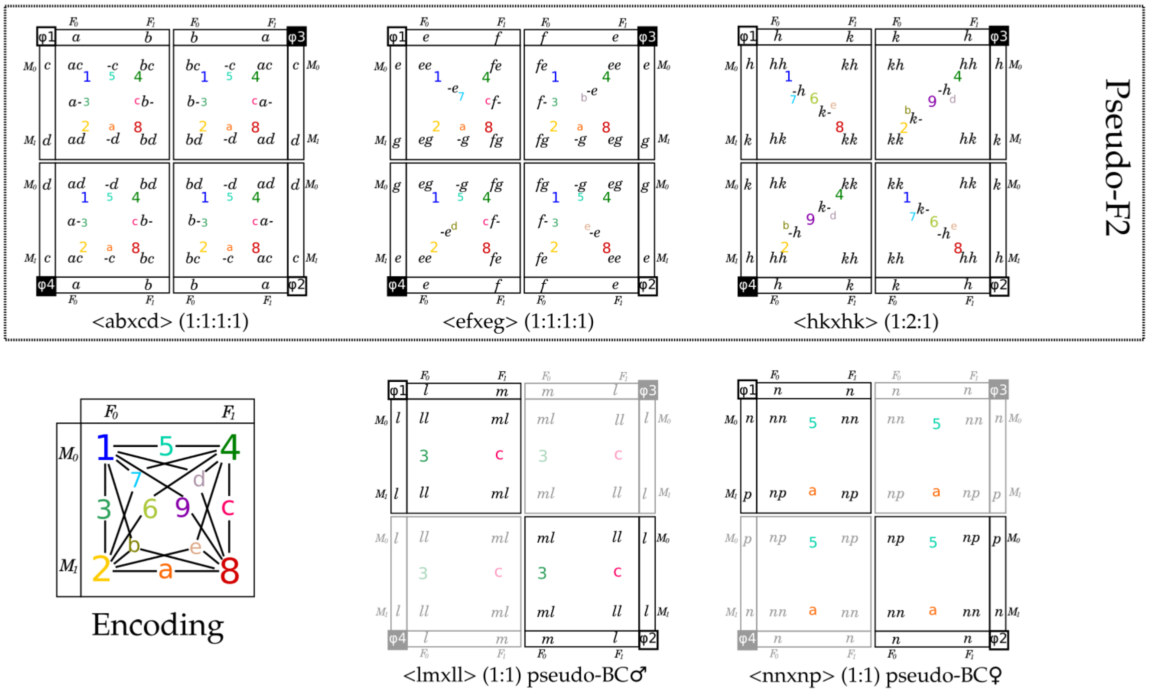

Figure 3: Diagram of the correspondance between phase codes, parental configurations, segregation types and encoding. Each box corresponds to a segregation type. Each quadrant of a box corresponds to a possible parental haplotypic configuration for a marker of this segregation type with its phase code in the corner (φ1...φ4). Black letters "a" through "m" represent the genotypes associated with the considered configuration and the colored characters "1" through "f" represent the hexadecimal encoding (table 4 page 4) used by `SeSAM`.

Based on these elements, we determine the phase of markers along the map construction process of the `autoMap` (4.1) pipeline as follows:

- In loadData (4.3.1), each pseudo-F2 marker is duplicated into two different putative markers, whose parental haplotypic configurations differ in one of the parents (for example AT x AT and TA x AT). Each of these markers, once loaded in the spellMapTools session, can then be considered (as explained above) as representing both the "direct" parental configuration and the "opposite" parental configuration; consequently, all four possible phases of pseudo-F2 markers will be considered. Pseudo-BC markers need not be duplicated. As before, they are also considered to represent both the "direct" and "opposite" parental configurations. The distinction between the two parental configurations of a marker will be made (by looking at the r_{ML} value) only once the marker has been fully tested and added to the map. A system of "phase" codes with values 1 to 4 is used to record parental configurations of markers relative to each other and to the actual configurations loaded into spellMapTools sessions. Figure 3 presents the correspondence between these phase codes, the parental configurations they represent for each segregation type and the encoding of segregations under these configurations.

- When measuring cross-linkage at any stage, implicitly or explicitly, all phases are considered between markers of different linkage groups. A marker may be linked to two linkage groups under different configurations.

- In generateSeeds (4.3.3), the parental configuration of the seed marker is arbitrarily determined. Thus, the parental configuration of a seed marker is the fixed reference starting phase against which all phases in the map are thereafter determined.

- In buildScaffold (4.3.4), we ensure that only one duplicate of any marker can ever be added to the map, this means that the selection process partially phases pseudo-F2 markers to be added. When a candidate passes all the tests and is added to the extremity of the scaffold, the actual phase is then determined by inverting, or not, the parental configuration of the candidate marker according to (1) whether the raw r_{ML} is above or under 0.5, and (2) whether the previous marker has itself been inverted. The chains of linkage and anti-linkage from the seed marker to the extremities allows the full phasing of the entire scaffold map. In buildMultiScaffold (4.3.4), all haplotypic configurations are considered again when measuring cross-linkage between scaffold maps of different linkage groups. Once a set of scaffold map replicates has been selected and filtered, the other duplicates of pseudo-F2 markers that were added to the scaffold may be discarded from the data.

- In assignment (4.3.5), both duplicates of a pseudo-F2 marker are tested to determine if the marker shows an overall significant preferential linkage to a single group. Once assigned, only the duplicate with the strongest linkage is assigned, the other one is discarded. The assignment process thus allows partial phasing of all assigned pseudo-F2 markers. All markers are then fully phased by looking at their r_{ML} values with the markers they are most strongly linked to.

- In buildFramework (4.3.6) and placement (4.3.7) SeSAM proceeds as with any other population types because all markers considered there are already phased.

10.6 Management of putative genotyping errors

10.6.1 Twins

During the segregation data processing step (4.3.1), as redundant markers are filtered out to compose a reduced list of markers for the scaffold and framework steps of the mapping process, markers of this set which have redundant peers are identified. These markers are marked as twins. Twin marker data is less likely to contain genotyping errors because unless there are very particular experimental systematic biases, the likelihood of two identical markers having the same genotyping error is very low.

During the scaffold building step (4.3.4), twin markers may take priority over selected candidates if they are sufficiently close ($<$ minimal distance) to the candidate. In the case of CP populations (10.5), candidate priority is based on two factors: whether the markers have a pseudo-F2 segregation type and whether the marker is a twin. Each factor gives markers priority over other markers, with pseudo-F2 segregation type being more important than being a twin.

During the framework building step (4.3.6), all twin markers are evaluated before any non-twin marker in order to give them priority in the map densification.

10.6.2 Singletons

A singleton is a case where two crossovers are found consecutively for the same individual in a triplet of consecutive markers in close proximity. Such singletons may betray a genotyping error in the middle marker.

At the end of the framework building step, singletons are identified in each marker according to the triplet they form with their two nearest neighbors. The segregation data of individuals identified as singletons are considered to be most likely the result of genotyping errors and are eliminated. The genetic distances of the framework map are then recalculated by EM using the filtered segregation data. This may result in a small bias towards smaller map distances because some true double-crossovers may be removed, but such bias is generally neglegible compared to the deleterious consequences of genotyping errors.

11 Basic functions for manual analyses

This section presents the use of some basic functions that use the `SpellMapTools` module to perform elementary calculations. The use of all these functions thus requires an initialized SpellMapTools session containing at least all the markers involved in the calculations. The initialization of a `SpellMapTools` session is covered in section 4.3.2 page 36.

11.1 2pt analysis

The following functions are used to perform two-point linkage analyses between pairs of markers in a matrix.

`SPELL_Lod2pt` returns the two-point LOD of linkage between each pair of markers. Note that for markers of a CP population, LODs may indicate that the parental configurations of the markers (or marker duplicates) are "linked" or "anti-linked" between the pair of markers as explained in section 10.5 page 67.

SPELL_Lod2pt

```
SPELL_Lod2pt(spellSession, mrkSet, mrkSet2=NULL, batchThr=200L)
```

Description:

Compute the two–point LOD of linkage between sets of markers.

Arguments:

spellSession a SpellMapTools object pointing to an active session.
mrkSet character vector, set of row markers. Will also be used as column markers (symetric matrix) if mrkSet2 is NULL.
mrkSet2 character vector, set of column markers.
batchThr number of markers in either set above which the computations will be split in batches.

Value:

Named matrix, two–point LOD.

SPELL_Rate2pt returns the recombination rates between each pair of markers. If correctRates is false, the rates returned will be the raw estimates from SpellMapTool's maximum likelihood function. If it is true, in CP populations the "anti-linked" rates will be corrected for the given population type as described in section 10.3 page 65 and section 10.5 page 66.

SPELL_Rate2pt

```
SPELL_Rate2pt(
  spellSession, mrkSet, mrkSet2=NULL, batchThr=200L,
  typePop=NULL, correctRates=TRUE
)
```

Description:

Compute the recombination rate between sets of markers.

Arguments:

spellSession a SpellMapTools object pointing to an active session.
mrkSet character vector, set of row markers. Will also be used as column markers (symetric matrix) if mrkSet2 is NULL.
mrkSet2 character vector, set of column markers.
batchThr number of markers in either set above which the computations will be cut in batches.
typePop A population type string (2.1.1) Used to apply relevant recombination rate corrections.
correctRates TRUE/FALSE whether or not to return corrected recombination rates.

Value:

Named matrix, recombination rates.

SPELL_Dist2pt returns the genetic distances between each pair of markers. If correctDists is false, the distances returned will be calculated from the raw recombination estimates from SpellMapTool's maximum likelihood function. If it is true, in CP populations the distances will be calculated from the corrected recombination rates for "anti-linked" markers as described in section 10.3 page 65 and section 10.5 page 66.

SPELL_Dist2pt

```
SPELL_Dist2pt(
  spellSession, mrkSet, mrkSet2=NULL, batchThr=200L,
  mappingFunction=SeSAM_par("mappingFunction"), typePop=NULL,
  correctDists=TRUE
)
```

Description:

Compute the genetic distance between sets of markers.

Arguments:

spellSession a SpellMapTools object pointing to an active session.
mrkSet character vector, set of row markers. Will also be used as column markers (symetric matrix) if mrkSet2 is NULL.

mrkSet2 character vector, set of column markers.
batchThr number of markers in either set above which the computations will be cut in batches.
mappingFunction "haldane" or "kosambi". Mapping function for genetic distances.
typePop A population type string (2.1.1) Used to apply relevant recombination rate corrections before applying the mapping function.
correctDists TRUE/FALSE whether or not to return corrected genetic distances.

Value:

Named matrix, genetic distance.

11.2 EM multipoint analysis

The `SPELL_SEM` function runs an Expectation-Maximization (EM) algorithm for multi-point analysis of a given sequence of loci with imputation of missing data, and returns a log-likelihood for this sequence as well as recombination rates between the consecutive markers. The recombination rates may be adjusted in the same manner as in `SPELL_Rate2pt` (11.1). Distances are computed as well from either raw or corrected recombination rates as in `SPELL_Dist2pt` (11.1). This function can perform multiple EMs in parallel.

SPELL_SEM

```
SPELL_SEM(
  spellSession, mrkOrder,
  mappingFunction=SeSAM_par("mappingFunction"),
  typePop=NULL, correctRates=TRUE, correctDists=TRUE
)
```

Description:

Compute the genetic distance between sets of markers.

Arguments:

spellSession a SpellMapTools object pointing to an active session.
mrkOrder character vector, sequence of marker names or list of such vectors for multiple SEMs.
mappingFunction "haldane" or "kosambi". Mapping function for genetic distances.
typePop A population type string (2.1.1) Used to apply relevant recombination rate corrections before applying the mapping function.

Value:

list:
$r: Recombination rates between subsequent markers.
$dist: Genetic distances between subsequent markers.
$logLik: log 10 likelihood of the marker order.
$converged: TRUE/FALSE. Whether the EM has converged.

The `SPELL_Try` function compares the placement of a marker in every interval in a given sequence of loci by running multipoint EM algorithms (in parallel) on each of the orders generated from adding the marker to the given sequence. The number of generated maps to test is $N + 1$ with N the number of markers in

the given sequence of loci. The sequences are compared by a LOD between the likelihood of each map and the likelihood of the map with the highest likelihood (LOD 0). The returned table is ordered by descending LOD starting with the highest likelihood map. This function can be used to run multiple Try tests (with argument recycling).

SPELL_Try

```
SPELL_Try(spellSession, mrkFrame, mrkPlace, minMultipointLod=NULL)
```

Description:

Compute the genetic map for a set of markers.

Arguments:

spellSession a SpellMapTools object pointing to an active session.
mrkOrder character vector, order of marker names or list of such vectors for multiple SEMs.
mrkFrame Character vector, order of marker for the frame in which positions for a new marker are tried. Can be a list of orders to perform multiple Tries. Will be recycled with 'mrkPlace'.
mrkPlace Character scalar, new marker for which the likelihood of all positions will be measured. Can be a vector or list of marker names to perform multiple Tries. Will be recycled with ' mrkFrame'.
minMultipointLod LOD (log 10 likelihood ratio to most likely position) threshold above which an order is significantly more likely than another. In this case, a filter to remove positions significantly less likely than the most likely position.

Value:

dataframe:
$lod: log 10 likelihood ratio of this position to the most likely position.
$left : Name of the maker in the frame left (perceeding in vector order) of this position. NA if preceeding the first marker.
Sorted by descending likelihood (ascending LOD). List of this dataframe structure for multiple Tries.

12 Debugging tools

Some of the main functions in the autoMap pipeline have optional outputs that may be activated through the `SeSAM_debugMode` function. This makes the functions output very verbose step by step reports of their progress. These reports are duplicated in text files:

- `buildMultiScaffold` outputs a debug file for each replicate detailing the candidate selections, tests and additions to the map. These files are outputted in the `scaffold_rep` folder of the pipeline output directory.
- `buildMultiScaffold` also outputs a debug table file for each replicate showing the status of each marker on each step. These files are outputted in the `scaffold_rep` folder of the pipeline output directory.

BL	Blacklisted
SC	In scaffold
SD	Seed marker
EX	Extremity marker
GL	Graylisted
<L	LOD to extremity too low
>D	Distance from extremity too high
<D	Distance from extremity too low
ST	Rejected due to segregation type
CF	Failed continuity test
TF	Failed Try test
3F	Failed 3-point test
AD	Added to scaffold
GE	Removed due to suspected genotyping error

- `buildFramework` outputs a debug file detailing what decision was taken for each candidate. This file is written in the pipeline output directory.
- `placement` outputs a debug file detailing what decision was taken for each candidate. This file is written in the pipeline output directory.

13 Quick-Start tutorial

```

#############################################################
##                                                         ##
##           TUTORIAL OF THE MAIN SeSAM FUNCTIONS          ##
##                                                         ##
#############################################################
#
# These are simple examples of basic use of the functions
# For details and complete list of arguments, see User Manual
# or use "? function"
#
# Each section starting with library(SeSAM) may be run
# independently from the others


###############################################################
##########   Simple automatic mapping from example   ##########
##########      BC1 data included in the package     ##########
library(SeSAM)
loadExample("testData")
SeSAM_par(nb_rep=2) # 2 scaffold replicates (2 seeds) per chromosome.
mrkInfo <- autoMap(
mapName="testData",
dirOut="SeSAM_example_testData",
segDataFile=segDataFileRaw,  # if NULL, looks for xxxx_segData.raw
# (or .loc or .gen) file in dirIn
phyMapFile=phyMapFile        # if NULL, looks for xxxx_phyMap.txt
# file in dirIn
)
```

```r


##############################################################
###########    Step-by-step automatic mapping    ############
library(SeSAM)
loadExample("testData")

# Load data, compute segegation distortion and non-redundant set of
    markers
mrkInfo <- loadData(
mapName="testData",
dirOut="SeSAM_example_testData",
segDataFile=segDataFileRaw,
phyMapFile=phyMapFile,
writose=TRUE, graphose=TRUE
)
head(mrkInfo)

# Pre-compute data in cache for the SPELL session
spellSession <- SPELL_cast(
segData=mrkInfo_after_loadData,
dirOut="SeSAM_example_testData/spell_session"
)

# Draw nb_rep seed markers for each chromosome
# based on phyMap chromosome assignment information
seeds_DF <- generateSeeds(
mrkInfo=mrkInfo_after_loadData,
spellSession=spellSession,
dirOut="SeSAM_example_testData", nb_rep=2,
verbose=TRUE, writose=TRUE, graphose=TRUE
)
head(seeds_DF)

# Build one scaffold map from each seed marker
# and for each chromosome, keeps the best
# scaffold among the nb_rep replicates
mrkInfo <- buildMultiScaffold(
seeds=seeds_DF,
mrkInfo=mrkInfo,
spellSession=spellSession,
dirOut="SeSAM_example_testData",
verbose=TRUE, writose=TRUE, graphose=TRUE
)
head(mrkInfo)

# Assigns each mappable marker to one of the chromosomes
# based on 2-point linkage to the scaffold maps
mrkInfo <- assignment(
mrkInfo=mrkInfo,
spellSession=spellSession,
dirOut="SeSAM_example_testData",
verbose=TRUE, writose=TRUE
)
```

```
head(mrkInfo)

# Densifies the scaffold maps with as many markers
# as possible while keeping marker order robust
# at the desired LOD threshold:
SeSAM_par("minMultipointLod")  # prints default value of LOD threshold
SeSAM_par(minMultipointLod=5)  # change LOD threshold for this SeSAM
    session
mrkInfo <- buildFramework(
mrkInfo=mrkInfo,
spellSession=spellSession,
dirOut="SeSAM_example_testData",
verbose=TRUE, writose=TRUE, graphose=TRUE
)
head(mrkInfo)
# When the genotyping error detection is ON (default), SeSAM will also
# generate "_framework_filtered" maps in which the effect of putative
# genotyping errors has been suppressed as much as mossible

# Places all remaining mappable markers in the total map
# based on where they map onto the framework map, but
# without altering the framework map
mrkInfo <- placement(
mrkInfo=mrkInfo,
spellSession=spellSession,
dirOut="SeSAM_example_testData",
verbose=TRUE, writose=TRUE, graphose=TRUE
)
head(mrkInfo)



##################################################################
###########    Functions to read, write, and convert    ###########
###########           segregation data files            ###########
library(SeSAM)
loadExample("testData")

segData <- readRaw(segDataFileRaw)

filename <- "aaaa"
writeRaw(
segData, filename, typePop=NULL,
naChar=SeSAM_par("naChar"),
nb_cpu=SeSAM_par("nb_cpu"), aliases=NULL, sep="\t",
verbose=FALSE, writose=FALSE, logFile=NULL
)

segData <- readLoc(segDataFileLoc)

filename <- "aaaa"
writeLoc(
segData, filename, typePop=NULL, popName=NULL, indivNames=NULL,
naChar=SeSAM_par("naChar"), nb_cpu=SeSAM_par("nb_cpu"),
```

```
verbose=FALSE, writose=FALSE, logFile=NULL
)

segData <- readGen(segDataFileGen, typePop="BC1")

filename <- "aaaa"
writeGen(
segData, filename, naChar=SeSAM_par("naChar"), sep="\t",
verbose=FALSE, writose=FALSE, logFile=NULL
)

# wrapper for any seg data file format. Will make encoding whenever needed
segData <- readSegData(  #  with raw file
segDataFileRaw, format=NULL, typePop=NULL, popName=NULL, indivNames=NULL,
naChar=SeSAM_par("naChar"), sep=""
)
segData <- readSegData(  # with loc file
segDataFileLoc, format=NULL, typePop=NULL, popName=NULL, indivNames=NULL,
naChar=SeSAM_par("naChar"), sep=""
)
segData <- readSegData(  # with gen file.  Here type="BC" must be specified
segDataFileGen, format=NULL, typePop="BC", popName=NULL, indivNames=NULL,
naChar=SeSAM_par("naChar"), sep=""
)

filename <- "aaaa.raw"
filename <- "aaaa.loc"
filename <- "aaaa.gen"
writeSegData(
segData, filename, format=NULL, typePop=NULL,
popName="aaaa", indivNames=NULL, naChar=SeSAM_par("naChar"),
nb_cpu=SeSAM_par("nb_cpu"),
raw_aliases=NULL, sep="\t",
verbose=FALSE, writose=FALSE, logFile=NULL
)


# ######################################################################################
########### Functions to read and write map data files ############
library(SeSAM)
filename <- SeSAM_consensusTest_map1()

genMap <- readGenMap(
filename, colId=1, colChr=2, colPos=3, colPha=NULL,
header=FALSE, sep="", naChars="-"
)

filename <- "aaaa.map"
writeMap(
map=genMap, filename, colId=1L, colChr=2L, colPos=3L, colPha=4L, sep='\t',
header=FALSE, verbose=FALSE, writose=FALSE, logFile=NULL
```

```
)


##########################################################################
###########    Simulate maps and segregation data sets      ############
###########  (can include missing and/or erroneous data)    ############
#
# Generates by simulation a genetic map,
# and simulates the corresponding segregation data
library(SeSAM)
dirOut <- "./simulation"
sim <-  simulatePop(
nbChr=2L,
nbMrk=c(50, 100),
mapLength=c(100, 200),  # in centiMorgans
nbIndiv=100,
typePop="BC1",
popName="sim_BC1",
interference = 1,        #  coefficient of coincidence (1 = no interference)
missingDataPercent = 0,
genotypingErrorPercent = 0,
missingDataGammaShape = c(mrk = NA, ind = NA),
genotypingErrorGammaShape = c(mrk = NA, ind = NA),
verbose=TRUE,
writose=TRUE,
dirOut=dirOut
)       # the encoding step can be quite long
genMap <- sim$genMap
segData <- sim$segData
phyMap <- sim$phyMap # here
rm(sim)

# Generates only a simulated genetic map
genMap <-  generateGenMap(
nbChr=2L,
nbMrk=c(50, 100),
mapLength=c(100, 200),  # in centiMorgans
verbose=TRUE,
writose=TRUE,
dirOut=dirOut
)

# Generates segregation data from a given genetic map
segData <- simulatePopFromMap(
genMap=genMap,
nbIndiv=100,
typePop="BC1",
popName="sim_BC1",
interference = 1,        #  coefficient of coincidence (1 = no interference)
missingDataPercent = 0,
genotypingErrorPercent = 0,
missingDataGammaShape = c(mrk = NA, ind = NA),
```

```
genotypingErrorGammaShape = c(mrk = NA, ind = NA),
verbose=TRUE,
writose=TRUE,
dirOut=dirOut
)



# ##############################################################################
###########    Compute a 'consensus' map from several map files ############
###########      Does not require segregation data, only maps ############
#
# The objective is to produce an output map as compatible as possible with the
# different input maps (see more details in the reference User Manual)
library(SeSAM)
consMap <- consensusMap(
SeSAM_consensusTest_map1(),
SeSAM_consensusTest_map2(),
SeSAM_consensusTest_map3()
)
# A graphical comparison of input and output maps is generated in:
# consensusMap/consensus_yyyy-mm-dd_hh-mm-ss_positions_graph.pdf


# #########################################################################
###########    Compute nb of crossovers to detect outliers ############
###########    and filter out individuals with too many COs ############

library(SeSAM)
loadExample("testData")

# Computes nbCO and returns it as an attribute of the object mrkInfo
#  attributes(mrkInfo)
mrkInfo <- computeCOPerIndiv(
mrkInfo=mrkInfo_after_buildFramework,
dirOut="SeSAM_example_testData",
verbose=TRUE, writose=TRUE, graphose=TRUE
)
nbCO <- getCOPerIndiv(mrkInfo)

# Filter outlier individuals having more than N COs
# (individuals potentially obtained by contamination)
# First compute a genetic map in automatic mode
loadExample("testData")
SeSAM_par(nb_rep=2)
```

```r
mrkInfo <- autoMap(
mapName="testData",
dirOut="SeSAM_example_testData",
segDataFile=segDataFileRaw,
phyMapFile=phyMapFile,
graphose=FALSE,
stopStep="framework"
)
# Then use the framework map file generated by autoMap()
genMapFile <- "SeSAM_example_testData/testData_framework_filtered_genMap.txt"

# Filters individuals with too many COs in outputted mrkinfo, based on
# the framework map information contained in the inputted mrkInfo
res <- filterIndivByNbCO(
mrkInfo, maxNbCO=5, typePop="BC",
popName=NULL, verbose=FALSE, writose=TRUE, graphose=FALSE,
logFile=NULL, dirOut=getwd(),
segData_format=c("raw", "loc", "gen"),
naChar=SeSAM_par("naChar"), nb_cpu=SeSAM_par("nb_cpu")
)
filteredMrkInfo <- res$mrkInfo
removedIndivs <- res$removed
# if writose=TRUE, the function will write a segData file without the
# individuals removed by the filtering on CO number

# idem but instead of taking a mrkInfo data frame, it
# reads the segData and map files from their file names
res <- filterSegDataFileByCO(
popName="testData", maxNbCO=5, dirIn=NULL, typePop="BC",
segDataFile=segDataFileRaw, genMapFile=genMapFile,
segData_format_in=NULL, segData_sep="",
nb_cpu=SeSAM_par("nb_cpu"),
genMap_colId=1L, genMap_colChr=2L,
genMap_colPos=3L, genMap_header=FALSE, genMap_sep="",
genMap_naChars='-',
verbose=FALSE, writose=TRUE, graphose=FALSE,
logFile=NULL, dirOut=getwd(),
segData_format_out=c("raw", "loc", "gen")
)
filteredMrkInfo <- res$mrkInfo
removedIndivs <- res$removed
# if writose=TRUE, the function will write a segData file without the
# individuals removed by the filtering on CO number



# ###########################################################################
###########     Create de novo linkage groups if no previous ############
###########         physical or genetic map is available ############
```

```r
library(SeSAM)
loadExample("testData")
spellSession <- SPELL_cast(segData=mrkInfo_after_loadData, dirOut="SeSAM_
    example_testData/spell_session")

putLGs <- putativeLGs(
mrkInfo=mrkInfo_after_loadData,
spellSession=spellSession,
dirOut="SeSAM_example_testData/Putative_LGs",
verbose=TRUE, writose=TRUE, graphose=TRUE
)



#######################################################
########### Functions for graphical output ############
#
# First functions to describe data
library(SeSAM)
loadExample("testData")

mappingCategoriesAreaPlot(mrkInfo_after_loadData, filename="aaaa.pdf")

mrkDistoHist(mrkInfo_after_loadData, filename="aaaa.pdf")

mrkMissingDataHist(mrkInfo_after_loadData, filename="aaaa.pdf")  # Error:
    private function

indMissingDataHist(mrkInfo_after_loadData, filename="aaaa.pdf")  # Error:
    private function

# Now compute the framework map to output a genMap that we
# will use to test graphical output functions
SeSAM_par(nb_rep=2) # 2 scaffold replicates (2 seeds) per chromosome.
mrkInfo <- autoMap(
mapName="testData",
dirOut="SeSAM_example_testData",
segDataFile=segDataFileRaw,
phyMapFile=phyMapFile,
stopStep="framework",
graphose=FALSE
)

# Plot the numbers of markers assigned to each chrom, or non assigned
assignmentBarplot(mrkInfo, filename="aaaa.pdf")   # Error: private
    function

# Reading the framework map data frame generated by autoMap()
genMap <- readGenMap("SeSAM_example_testData/testData_framework_filtered_
    genMap.txt")
phyMap <- readPhyMap(phyMapFile)

# Plot genetic positions vs physical positions
mareyMap(
```

```
phyMap=phyMap,
genMap=genMap,
mapName="testData",
filename="aaaa.pdf"
)

# Plot 2-point LOD matrix as a heat map
spellSession <- SPELL_cast(segData=mrkInfo_after_loadData,dirOut="SeSAM_example_testData/spell_session")
lod2ptHeatMap(
spellSession,
map_DF=genMap,  # we have chosen above genMap to be the framework map
mapName="testData",
mrkLab = NULL,
segType = NULL,
mrkHighlight = NULL,
labHighlight = NULL
)

# Plot 2-point distance matrix as a heat map
dist2ptHeatMap(
spellSession,
map_DF=genMap,  # we have chosen above genMap to be the framework map
mappingFunction = SeSAM_par("mappingFunction"),
typePop = "BC",
mapName="testData",
mrkLab = NULL,
segType = NULL,
mrkHighlight = NULL,
labHighlight = NULL
)




########################################################################
###########     Functions for "manual" 2-point analysis     ############

library(SeSAM)
loadExample("testData")
spellSession <- SPELL_cast( segData=mrkInfo_after_loadData, dirOut="SeSAM_example_testData/spell_session" )

# First compute the framework map to output a genMap
SeSAM_par(nb_rep=2) # 2 scaffold replicates (2 seeds) per chromosome.
mrkInfo <- autoMap(
mapName="testData",
dirOut="SeSAM_example_testData",
segDataFile=segDataFileRaw,
phyMapFile=phyMapFile,
stopStep="framework",
graphose=FALSE
)
genMap <- readGenMap("SeSAM_example_testData/testData_framework_filtered_
```

```r
    genMap.txt")
mrkSet <- genMap$mrkName

# Computes the 2-point LOD matrix of the ordered framework markers
lod2ptMatrix <- SPELL_Lod2pt(spellSession, mrkSet, mrkSet2=NULL, batchThr=200L)
head(lod2ptMatrix)

# Computes the 2-point Distance matrix of the ordered framework markers
dist2ptMatrix <- dist2ptMatrix <- SPELL_Lod2pt(spellSession, mrkSet, mrkSet2=NULL, batchThr=200L)
head(dist2ptMatrix) # in centiMorgans

# E-M multipoint computation of distances and likelihoods from an ordered set of markers
sem <- SPELL_SEM(
spellSession, mrkOrder=mrkSet,
mappingFunction=SeSAM_par("mappingFunction"),
typePop=NULL, correctRates=TRUE, correctDists=TRUE
)
head(sem$r)      # recombinaiton fractions
head(sem$dist)   # genetic distances in cM
sem$logLik       # log-likelihood of the map

# Determines the best interval in the framework map where the
# marker "mrkPlace" maps to (similar to mapmaker's "try" command ;)
mrkPlace <- genMap$mrkName[5]    # marker to be placed
mrkFrame <- genMap$mrkName[-5]   # framework map
tryRes <- SPELL_Try(spellSession, mrkFrame, mrkPlace, minMultipointLod=NULL)
head(tryRes, 15)   # tryRes$lod indicates



# #############################################################################

########### Functions to save and restore a SpellMapTools session ############

library(SeSAM)
loadExample("testData")
spellSession <- SPELL_cast( segData=mrkInfo_after_loadData, dirOut="SeSAM_example_testData/spell_session" )
SPELL_saveSessionInfo( spellSession=spellSession, filename="SeSAM_example_testData/spell_session/sessionInfo.RData")
q()  # the session is now saved, you can exit R

R  # next R session where you want to re-use the previous SpellMapTools cache
library(SeSAM)
loadExample("testData")
spellSession <- SPELL_recallFromInfoSave( filename="SeSAM_example_testData/spell_session/sessionInfo.RData" )
```

```
#  you can now proceed with further mapping analyses.
#  for instance 2pt linkage analyses, scaffold construction, etc.
```

References

de Givry, S., Bouchez, M., Chabrier, P., Milan, D., & Schiex, T. (2004). Carhta Gene: multipopulation integrated genetic and radiation hybrid mapping. *Bioinformatics*, *21*(8), 1703–1704.
URL https://doi.org/10.1093/bioinformatics/bti222

Haldane, J. (1919). The combination of linkage values and the calculation of distances between the loci of linked factors. *J Genet*, *8*(29), 299–309.

Haldane, J., & Waddington, C. (1931). Inbreeding and linkage. *Genetics*, *16*(4), 357.

Jasson, S., & Leroux, D. (2017). Spell-qtl, a new tool for qtl analysis on modern datasets. *PAG XXV - Plant and Animal Genome Conference, Jan 2017, San Diego, United States*.

Kosambi, D. (1944). The estimation of map distances from recombination values. *Ann. Eugen.*, *12*, 172–175.

Lander, E. S., Green, P., Abrahamson, J., Barlow, A., Daly, M. J., Lincoln, S. E., & Newburg, L. (1987). Mapmaker: an interactive computer package for constructing primary genetic linkage maps of experimental and natural populations. *Genomics*, *1*(2), 174–181.

Stam, P. (1993). Construction of integrated genetic linkage maps by means of a new computer package: Join map. *The plant journal*, *3*(5), 739–744.

Winkler, C. R., Jensen, N. M., Cooper, M., Podlich, D. W., & Smith, O. S. (2003). On the determination of recombination rates in intermated recombinant inbred populations. *Genetics*, *164*(2), 741–745.
